# Immunity Debt for Seasonal Influenza After the COVID‐19 Pandemic and as a Result of Nonpharmaceutical Interventions: An Ecological Analysis and Cohort Study

**DOI:** 10.1002/advs.202410513

**Published:** 2025-04-05

**Authors:** Li Chen, Yuchen Guo, Kim López‐Güell, Jun Ma, Yanhui Dong, Junqing Xie, Daniel Prieto Alhambra

**Affiliations:** ^1^ Centre for Statistics in Medicine and NIHR Biomedical Research Centre Oxford NDORMS University of Oxford Oxford OX3 7HE UK; ^2^ Institute of Child and Adolescent Health School of Public Health Peking University Beijing 100083 China; ^3^ Department of Medical Informatics Erasmus University Medical Center Rotterdam 3015 GD the Netherlands

**Keywords:** COVID‐19, immunity debt, influenza, nonpharmaceutical interventions

## Abstract

Non‐pharmaceutical interventions (NPIs) during the COVID‐19 pandemic significantly reduced influenza transmission. This study explores the hypothesis of “immunity debt” which suggests increased vulnerability to influenza following reduced exposure during the pandemic. World Health Organization aggregated data on influenza from 116 countries and its association with NPI intensity as measured by the COVID‐19 Stringency Index is analyzed. Where individual‐level data available (France, the United Kingdom, Spain, Italy, Belgium, and Romania), the analyses of influenza monthly rates in six European countries (France, the United Kingdom, Spain, Italy, Belgium, and Romania) are replicated. The results indicate globally a 46.3% (95% CI: 15.79–70.78%) reduction in influenza cases during COVID‐19 restrictions in the winter season, followed by a 131.7% (95% CI: 34.95–255.78%) increase in the first postrelaxation winter and a 161.2% (95% CI: 31.88–382.16%) increase in the summer as compared to the predicted level based on historical influenza epidemic trends. In addition, a positive association between the Stringency Index and post‐relaxation influenza surge is observed globally (R^2^ = 0.14–0.17) and replicated regionally. The findings support the population immunity debt hypothesis for influenza and call for proactive preparations against its consequences in future pandemics.

## Graphical abstract

This study provides support for the immunity debt hypothesis, revealing a 131.72% increase in influenza rates during winter and a 161.23% rise in summer, 1 year following the global easing of COVID‐19 restrictions. This study offers empirical evidence of immunity debt on a global level, highlighting the importance of incorporating this concept into the formulation of long‐term pandemic response strategies.

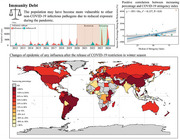

## 1 Introduction

A variety of nonpharmaceutical interventions (NPIs), including social distancing, mask‐wearing, shelter‐in‐place, travel restrictions, and school closure,^[^ ^1^, ^2^ ^]^ were implemented to reduce the spread of SARS‐Cov‐2 virus during the COVID‐19 pandemic.^[^ ^3^ ^]^ As a side effect of these NPIs, the transmission of many other infectious pathogens was also curbed,^[^ ^4^, ^5^, ^6^, ^7^, ^8^, ^9^, ^10^, ^11^ ^]^ with a substantial decrease in incidences observed for illnesses, such as influenza, respiratory syncytial virus, and Mycobacterium tuberculosis.^[^ ^7^, ^8^, ^12^, ^13^, ^14^, ^15^, ^16^ ^]^ However, a resurgence of these common respiratory illnesses was noted after the relaxation of NPIs, particularly during the first winter. For example, in China, as of December 2023, an early and unusually high cluster of undiagnosed pneumonia was recorded in children in northern China.^[^ ^17^, ^18^ ^]^ A similar increase in respiratory infections was also observed in the United Kingdom and the United States.^[^ ^18^, ^19^ ^]^ This rebound has led researchers to propose the hypothesis of immunity debt.

The hypothesis of immunity debt suggests that the population may have become more vulnerable to other non‐COVID‐19 infectious pathogens due to reduced exposure during the pandemic. As a result, a waning of immunity against these microorganisms could occur.^[^ ^3^, ^18^, ^20^ ^]^ While several studies have attempted to quantify the immunity debt, the hypothesis remains unconfirmed due to limitations, such as a focus only on children under five, a lack of objective measurements for COVID‐19 restrictions’ stringency, and an inconsistent definition for the status of immunity debt.^[^ ^19^, ^20^ ^]^

Given the fact that the extent of NPIs varies significantly across countries and regions, it provides an unprecedented natural experiment to study this unexplained phenomenon. Thus, based on global influenza virological surveillance data^[^ ^21^ ^]^ and the COVID‐19 Stringency Index,^[^ ^22^ ^]^ this study aimed to 1) delineate the global and continental epidemiology of influenza during and after the periods of COVID‐19 restriction, and to 2) test the hypothesis of immunity debt at the population level. Uniquely, we leveraged readily available primary care electronic health records from six European countries to replicate the ecological analyses to demonstrate the robustness of our findings.

## 2 Result

Between 2012 and 2024, 4581080 influenza cases were reported spanning 116 countries across six continents. The number of cases for all countries is presented in Table S1 (Supporting Information).

### 2.1 COVID‐19 Restriction Period and Relaxation Period for 116 Countries

**Figure 1:**
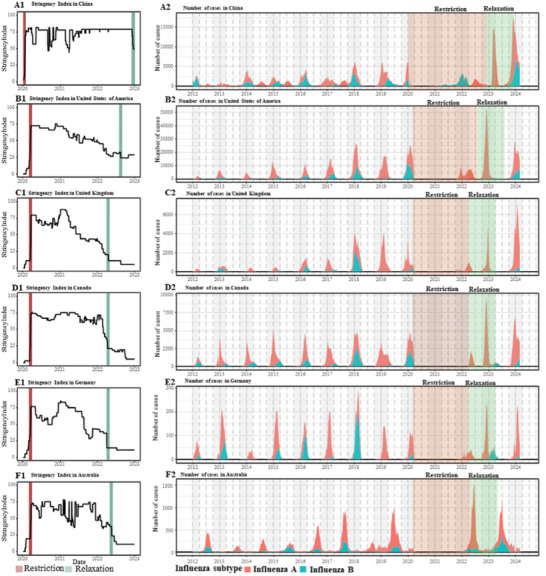
Fluctuation of stringency index and influenza cases during relevant COVID‐19 restriction periods across six illustrative countries. A1‐F1 presents the COVID‐19 stringency index in six selected countries (China, the United States of America, the United Kingdom, Canada, Germany, and Australia); The red bar indicates the start date, and the blue bar indicates the final release of COVID‐19 restrictions. The gap between the red and blue bars is defined as the restriction period. A2‐F2 presents the number of total influenza cases of six representative countries (China, the United States of America, the United Kingdom, Canada, Germany, and Australia). The window with a red background represents the defined restriction period, while the window with a blue background represents the one‐year follow‐up period after the final release of COVID‐19 restrictions.

**Figure** **1** presents the initial month of COVID‐19 restrictions period and relaxation period, and the number of influenza cases in six illustrative countries: China, the United States of America, the United Kingdom, Canada, Germany, and Australia. The initial month of the COVID‐19 restriction period was temporally proximate across countries; however, the initial month of the relaxation period varied. China was the latest among these countries, initiating its relaxation period in December 2022. The initial month of COVID‐19 restriction period and relaxation period for 116 countries is presented in Figures S2–S12 (Supporting Information). The mean, median, and maximum stringency indices during the COVID‐19 restriction period are summarized in Table S2 (Supporting Information). Additionally, a global map depicting these indices is presented in Figure S13 (Supporting Information). Throughout the COVID‐19 restriction period, countries implemented various stringent measures. Notably, the stringency index in the African region was lower compared to other regions, indicating a relative leniency in the measures adopted there.

Influenza demonstrates distinct seasonal patterns that vary by country. For instance, the peak influenza seasons in China occur in the winter season, whereas in Australia, they typically manifest in the summer season. The peak influenza seasons of countries in the same continent are similar. The imposition of COVID‐19 restrictions has altered these seasonal trends, resulting in the attenuation or complete elimination of the typical flu peak in some countries. During the COVID‐19 restriction period, the observed levels of influenza activity were relatively lower than those recorded between 2012 and 2019 (Figures S14–S24, Supporting Information).

The seasonal influenza characteristics returned to the historical seasonal patterns of influenza after the lifting of COVID‐19 restrictions. During the 1‐year relaxation period (one year after the lifting of restrictions), the majority of countries displayed seasonal influenza characteristics consistent with historical observations. Concurrently, during the first epidemic season of this relaxation period, most countries reported influenza activity levels that exceeded those recorded in the corresponding historical periods. In six representative countries, the first epidemic season saw higher than historical levels of influenza activity in China, the United States, and Canada. Conversely, some countries, such as Germany and the United Kingdom, showed influenza activity levels that were comparable to their historical norms.

### 2.2 Model Selection

The evaluation of five distinct time series models is summarized in **Table** **1**, including a range of metrics such as mean square error (MSE), normalized MSE, RMSE, and RMSE/Mean. Given the observed variability in the weekly number of reports, both normalized MSE and RMSE/Mean were used as the primary metrics to assess the performance of each model. Among these, the historical model, which uses weekly averages of historical data, recorded an MSE of 413663—the highest recorded among the models. Despite this, it demonstrated the lowest Normalized MSE of 1.1 and an RMSE/Mean of 1.6 in the validation dataset (2018–2019), indicating superior performance concerning normalization and ratio metrics. Although predicated on weekly averages of historical data, the historical model has shown enhanced applicability across a broader array of nations compared to its counterparts. Consequently, we used the historical model as the primary modeling approach for the subsequent analyses.

**Table 1:** The performance of model selection.

Model	MSE	Normalized MSE	RMSE	RMSE/ Mean
ARIMA	363 893	1.2	163.1	1.8
Negative binomial generalized linear model	197 310	1.4	128.8	1.7
Historical model	413 663	1.1	152.5	1.6
Prophet	327 361	1.3	141.8	1.6
Quasi‐poisson generalized linear model	149 147	1.5	113.6	1.7

Based on the historical model, the counterfactual predictions for the periods of COVID‐19 restrictions period and relaxation period were utilized to quantify the reduction and increase in the influenza activity levels attributed to COVID‐19 restrictions.

### 2.3 Increase During Relaxation Period

In an analysis of influenza case numbers across 116 countries during two specific quarters following relaxation periods—Quarter 14 (winter seasons) and Quarter 23 (summer seasons)—we observed a marked increase in reported cases.

**Figure 2:**
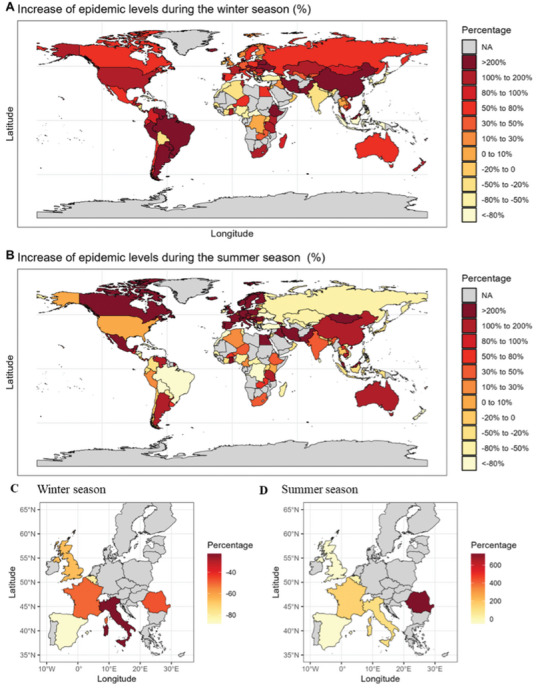
Changes of an epidemic of any influenza after the release of COVID‐19 restriction, stratified by winter seasons and summer seasons. Panels A and B are derived from FluNet data, while Panels C and D utilize patient‐level data from the THIN databases.

In the winter season of the relaxation period, 50 out of 116 countries reported an uptick in influenza cases, with a global weighted increase of 131.72% (95% CI: 34.95–255.78%). Notably, South America experienced the most significant surge at 715.29% (95% CI: 336.79–1247.49%), driven largely by high increases in Argentina, Venezuela, and Uruguay. Additionally, significant rises were also observed in populous nations, with China and the United States reporting increases of 222.80% (95% CI: 80.56–378.38%) and 157.48% (95% CI: 37.06–303.23%), respectively (**Table** **2**). As illustrated in **Figure** **2**A, except for Africa—which experienced a modest average increase of 51.94% (95% CI: −6.34 to 130.00%) in influenza cases—countries from other continents displayed significantly higher rises during the winter season. Specifically, 40 countries reported significant increases exceeding 100%, including the United States of America (157.48%, 95%CI: 37.06–303.23%), and 19 countries recorded significant increases over 200%, including China (222.80%, 95%CI: 80.56 to 378.3%). In the replication of patient‐level data, no increase was observed during the winter season (Figure 2C).

**Table 2:** The immunity debt across continents and the top five countries of each continent.

Winter seasons			Summer seasons		
	Percentage of changes	95% CI		Percentage of changes	95% CI
Global	131.72	34.95–255.78	Global	161.23	31.88–382.16
Africa	51.94	−6.34 to 130.00	Africa	30.54	−17.82 to 105.02
Ethiopia	301.20	179.88–453.85	Ghana	370.64	121.02–698.36
United Republic of Tanzania	179.07	65.12–333.73	Egypt	264.49	183.68–375.15
Kenya	156.41	76.85–250.12	Mauritius	190.53	46.94–407.00
Ghana	137.64	59.03–259.63	United Republic of Tanzania	187.87	94.87–328.87
South Africa	131.56	18.02–292.81	Zambia	76.91	45.06–122.51
Asia	95.88	17.85–190.25	Asia	143.50	31.61–307.97
Malaysia	1929.91	1461.90–2466.53	Bahrain	8060.00	5503.37–11 471.60
Bahrain	650.56	295.63–1116.66	Malaysia	1863.64	1478.68–3018.84
Iran (Islamic Republic of)	525.71	227.80–999.20	Pakistan	1178.11	694.53–1985.54
Pakistan	424.69	235.40–675.44	Iran (Islamic Republic of)	717.23	349.31–1229.10
China	222.80	80.56–378.38	Mongolia	460.53	87.87–1005.89
Europe	146.63	41.33–281.24	Europe	772.56	200.35–1982.53
Ukraine	887.70	448.94–1392.53	Portugal	6887.25	3137.83–13 399.13
Czechia	782.39	367.27–1301.23	Albania	6071.43	922.42–31 846.34
Denmark	369.24	199.09–591.95	Netherlands (Kingdom of the)	3147.03	1039.68–6219.69
Portugal	352.80	197.40–528.99	Switzerland	2038.87	246.86–5970.02
Belarus	319.21	167.93–530.80	Luxembourg	1598.63	373.56–4517.41
North America	113.00	14.38–234.79	North America	62.12	22.36–114.76
Panama	171.23	−32.65 to 484.19	Mexico	227.47	174.00–299.38
United States of America	157.48	37.06–303.23	Canada	207.72	58.02–399.99
Honduras	147.10	−46.66 to 514.75	Guatemala	93.97	40.15–161.38
Guatemala	114.76	48.36–199.70	Panama	51.07	−18.57 to 149.62
Canada	67.16	−16.02 to 162.87	Costa Rica	7.76	−32.17 to 56.03
Oceania	107.09	38.31–334.96	Oceania	88.57	32.70–222.96
Fiji	1174.69	553.46–2123.54	Australia	125.78	62.20–279.91
New Zealand	106.99	−23.30 to 1128.26	Fiji	87.16	−61.05 to 409.87
Australia	68.94	32.03–114.71	New Zealand	−100.00	−100.00 to ‐100.00
South America	715.29	336.79–1247.49	South America	−27.62	−58.53 to 21.30
Argentina	4253.23	2732.94–6213.25	Venezuela (Bolivarian Republic of)	116.72	−9.30 to 350.51
Venezuela (Bolivarian Republic of)	1299.64	511.43–2406.35	Argentina	109.21	35.99–207.18
Uruguay	719.21	313.52–1389.86	Uruguay	54.33	−6.48 to 132.30
Paraguay	315.87	174.85–495.23	Peru	25.78	−44.60 to 137.66
Peru	292.28	185.89–419.15	Chile	−1.81	−50.49 to 56.61

the winter season was defined as the first and fourth quarters (quarter 14), while the summer season corresponded to the second and third quarters (quarter 23).

In the summer season, 54 countries reported an increase in influenza cases, and global weighted increases of 161.23% (95% CI: 31.88–382.16%), with notable rises across all regions except South America and Africa, with Asia and Europe particularly affected (Table 2). The magnitude increase in the number of cases in some countries in the summer season is lower than that in the winter season, mainly in Africa (30.54 95%CI: −17.82 to 105.02) and South America (−27.62, 95%CI: −.53 to 21.30) (Figure 2B). 35 countries reported significantly increases exceeding 100%, including China (171.70%, 95%CI: 13.64–378.10%), and 28 countries recorded significantly increases over 200%, including the United Kingdom (241.89%, 95%CI: 69.29–504.86%).In the replication of patient‐level data, an increase in the number of influenza cases was also observed in Belgium, France, Italy, and Romania in the summer season. Romania exhibited the highest increase (725.53%) (Figure 2D).

For influenza A, the number of cases decreased in 80 of the 116 countries during the winter season of the relaxation period, with a global weighted increase of 168.71% (95% CI: 43.84 to 336.52%). During the summer season of restriction period, 47 countries reported a significant increase, with a global increase of 245.91% (95% CI: 55.88–577.39%). In South America, the substantial increases occurred primarily in the summer season, with an increase of 907.56% (95% CI: 354.68–1758.81%) (Table S6 and Figure S28, Supporting Information). For influenza B, the global weighted significant increases in number of cases in winter season were observed (85.58%, 95% CI: 3.93–196.05%), mainly in South America (403.62%, 95% CI: 136.65–769.00%) (Table S7 and Figure S29, Supporting Information).

### 2.4 Association Between Stringency Index and Increases During Relaxation Period

**Figure 3:**
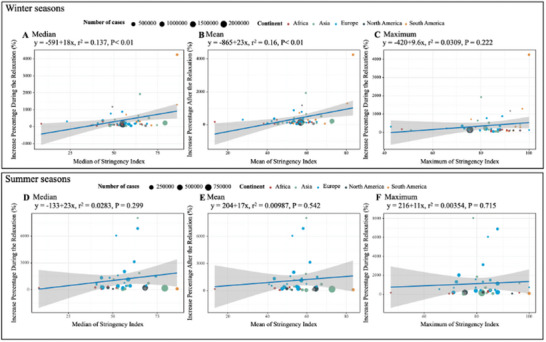
Relationship between stringency of COVID‐19 restrictions and changes of influenza epidemic level after the release of COVID‐19 restrictions across countries.

**Figure** **3** illustrates the association between the increase in total influenza activity level and the stringency index. During winter seasons of the relaxation period, the increased percentage of case numbers showed a positive association with the median, mean, and maximum stringency index, with significant associations observed for the median and mean values (*p* < 0.01 for both). Although a positive association was also observed in summer seasons, the correlations with the median, mean, and maximum values did not achieve statistical significance.

In the analysis presented in Figure S33 (Supporting Information), a statistically significant positive correlation was identified between the increase in influenza A cases and both the median and mean stringency indices during winter seasons (*p* < 0.01 for both). Influenza A, being the predominant subtype, primarily drove the observed positive association between increased influenza activity and stringency index measures within this period. While a similar positive trend was noted in summer seasons, it did not reach statistical significance. Furthermore, although influenza B demonstrated a positive correlation with stringency indices, this association lacked statistical significance, as shown in Figure S34 (Supporting Information). The results of the association between the Stringency Index and reduction during the restriction period were provided in the supplementary text.

### 2.5 Replication Using Individual Patient‐Level Data

In patient‐level data, we used a consistent process to calculate the reduction in daily influenza cases during the restriction period and the increase during the relaxation period. The reduction and increases in the Patient‐level data were shown in Figures S35 and S36 (Supporting Information). We observed a positive association between the stringency index and the increase in influenza cases during the relaxation period (Figure S38, Supporting Information).

For chronic diseases used as negative control outcomes (chronic obstructive pulmonary disease (COPD) and hypertension), we did not observe increases in the patient‐level data (Tables S8 and S9, Supporting Information). While we observed a reduction in chronic diseases during the restriction period, there was no positive association between the stringency index and this reduction (Figures S39 and S40, Supporting Information).

## 3 Discussion

We presented the first study to evaluate the hypothesis of immunity debt for seasonal influenza as a result of COVID‐19 restrictions at a global level and using both aggregated surveillance as well as individual‐level electronic health records data. Our study found that the levels of influenza transmission after lifting COVID‐19 restrictions were globally associated with the intensity of pandemic‐related NPIs, supporting the hypothesis of immunity debt. During the winter seasons, a mean global reduction of 46.30% in seasonal influenza was observed, with three in four countries reporting significant reductions compared to historical data. After lifting restrictions, influenza transmission bounced back with a global average increase of 131.72%, and almost 70% of countries reported increased transmission levels compared to pre‐pandemic levels. Additionally, a further average increase of 161.23% in summer influenza cases was reported, with almost half of the countries reporting significant increases one year after relaxing COVID‐19 NPIs.

Importantly, the level of COVID‐19 NPI restrictions as measured by the Oxford stringency index^[^ ^22^ ^]^ appeared associated with the observed increase in seasonal influenza post‐pandemic. Similarly, countries implementing more stringent NPIs exhibited greater increases in influenza cases in the year following the relaxation of these measures. Our data therefore supported the *immunity deb*t hypothesis. The concept of “immunity debt” posited that prolonged and more stringent NPIs could lead to reduced exposure to pathogens, consequently diminishing herd immunity.^[^ ^19^, ^20^ ^]^ Specifically, our findings suggested that more intense NPIs, quantified through the COVID‐19 stringency index, had been linked to greater immunity debt.

Our findings highlight the importance of adopting a stepwise approach to relaxing the lasting restrictions that were put in place to control the spread of novel infections. Such measures were undoubtedly essential for managing the pandemic emergency, their potential long‐term impact on the natural immunological development within populations should be given greater consideration in future public health policies. It is crucial to have alleviation strategies in preparation for any similar scenarios in the future. For instance, once effective vaccines become available, implementing a phased relaxation of NPIs based on the proportion of the vaccinated population could help minimize the risk of weakened immunity to historically existing infectious illnesses and simultaneously minimize hospitalizations and mortality from emerging pathogens.

Our research expanded the scope of existing literature by providing a global perspective on the impact of COVID‐19 restrictions, unlike previous studies which primarily focused on individual countries.^[^ ^19^, ^20^ ^]^ By utilizing the stringency index to determine the COVID‐19 restriction and relaxation periods for each country, we established a methodological foundation for quantifying immunity debt across various nations. Furthermore, the variation in the intensity of the stringency index provided a unique opportunity to enable an evaluation of the potential relationship between COVID‐19‐related NPIs and immunity debt. Distinct from prior research that predominantly targeted children under five,^[^ ^19^, ^20^ ^]^ our study included data across all age groups. This broader approach not only supports the hypothesis but also acknowledges the emphasis of previous research on respiratory infections in young children, who are at a critical stage of immune system development. Low‐level exposure during this formative period could result in a pronounced immunity debt. However, our findings indicated that this phenomenon is not limited to the pediatric population but could also manifested across other demographic groups, suggesting broader implications for public health strategies post‐pandemic.

Although the findings of this study lend support to the immune debt hypothesis, we contend that immune debt alone does not account for the observed increase in influenza activity following the relaxation of restrictions completely. Influenza activity was multifactorial, and while immune debt may have contributed to this increase, other factors must also be considered. These included the reduced exposure to the virus during periods of stringent restrictions and the subsequent surge in social and communal activities post‐relaxation, which likely contributed to the increased transmission of influenza.^[^ ^23^, ^24^ ^]^ Thus, the dynamics of influenza spread are influenced by a complex interplay of behavioral and immunological factors. However, it is critical to clarify that the concept of immunity debt did not suggest that NPIs should not be implemented. NPIs serve as essential community mitigation strategies, particularly during the initial phases of outbreaks involving novel pathogens, when vaccines are unavailable and population immunity is minimal or absent.^[^ ^25^ ^]^ To mitigate the unintended consequences of immunity debt associated with restrictions, future pandemic response strategies should incorporate several measures, including vaccinating susceptible populations, timing the relaxation of restrictions judiciously, and avoiding the relaxation of NPIs during peak influenza seasons. Such strategies aimed to prevent the concurrent burden of influenza and emerging viruses, which could significantly strain healthcare systems.

The dynamics of influenza incidence during the COVID‐19 pandemic and the subsequent relaxation period display significant fluctuations, closely aligned with the typical epidemic seasons of influenza across different continents. During the restriction period, numerous studies document a notable reduction in influenza cases across various countries.^[^ ^6^, ^7^, ^8^, ^9^, ^11^ ^]^ This reduction can be attributed to the NPIs implemented to mitigate the spread of COVID‐19, which inadvertently limited exposure to other pathogens as well.^[^ ^5^, ^8^, ^12^, ^14^, ^16^ ^]^ This suggests that NPIs are effective not only against COVID‐19 but also in controlling a wide array of infectious diseases. However, following the relaxation of these restrictions, a resurgence in respiratory infections, particularly respiratory syncytial virus and influenza, has been observed. This increase has been extensively reported in prior research and highlights a rebound effect where the incidence of respiratory infectious diseases increased post‐relaxation. However, in addition to the increases observed during the peak influenza seasons (winter seasons),^[^ ^26^ ^]^ notable increases have also been seen in summer seasons, an earlier flu season with historically lower pandemic levels, which can partly explain the significant increases noted, particularly for influenza A. This indicates that the surge in cases was not limited to the influenza season alone but may also have influenced the seasonal characteristics and the timing following the relaxation of restriction. This broader impact suggests that the dynamics of influenza transmission extend beyond traditional seasonal patterns, potentially altering the epidemiological landscape in the post‐pandemic period.

A major strength of this study lies in its robust methodology, which includes the precise determination of the COVID‐19 restriction and relaxation periods for each country using global data. Additionally, the study utilizes influenza data collected from the United Nations influenza surveillance system, covering individual countries worldwide. This approach enables a comprehensive assessment of the association between influenza activity levels and COVID‐19 restrictions across different countries and regions, thereby testing the hypothesis of immunity debt. The utilization of global data provided by governments ensures that our findings were broadly generalizable, and reinforces the validity of the immunity debt hypothesis across diverse geopolitical contexts.

We outlined several limitations. The influenza data sourced from the Global Influenza Programme represents only a fraction of potential influenza cases, as not all suspected cases are tested in laboratories to confirm the presence of influenza or to identify the specific strain. A limitation of our study is the potential impact of the SARS‐CoV‐2 pandemic on FluNet data. With public health resources redirected to managing COVID‐19, influenza surveillance sensitivity and completeness may have been compromised.^[^ ^27^ ^]^ The extent of laboratory testing varies significantly between countries and can fluctuate over time. Additionally, another limitation pertains to the variability in levels of laboratory testing; it was difficult to determine whether the observed increases in influenza cases during relaxation periods in some countries truly reflect a genuine rise in infections, or if they were the result of improved detection capabilities that followed enhanced surveillance measures during the COVID‐19 pandemic. Consequently, the increases noted 1 year after relaxation might be overestimated. However, in countries like the USA, UK, and China, which were considered to have high detection capabilities, an increase in influenza activity is still observed. Moreover, the absence of data specific to different age groups limits our ability to assess the impact of immunity debt across these demographics. For all countries, two periods (COVID‐19 restriction period and relaxation period) are applied to define the COVID‐19‐related NPIs, making it challenging for us to discern the relative effects of various interventions that are implemented at different stages of the pandemic response. Consequently, we can only provide a crude estimate of the overall impact of NPIs. Additionally, our data is presented at the national population level, which might obscure subnational variations in influenza activity. Previous studies have highlighted the strain‐specific variability in influenza resurgences observed in China and the United States in 2021,^[^ ^28^ ^]^ with novel variants potentially altering both the severity and timing of influenza surges. Such variations may influence the associations identified in our analysis. To further elucidate these dynamics, future research should incorporate strain‐specific data to examine how viral mutations interact with public health interventions and their subsequent impact on influenza resurgence.

While the COVID‐19 Stringency Index from OxCGRT has some limitations, including inconsistencies and reporting gaps, particularly in representing the full range of policies during the 2021–2023 period,^[^ ^22^ ^]^ it remains a valuable tool for comparing government responses across countries. The effectiveness of NPIs is influenced by socioeconomic factors and cultural practices, which varied widely across countries. These variables could affect the levels of influenza activity, and it is challenging to quantify and control for these factors within the scope of this study. However, the observed reduction in influenza activity during the COVID‐19 restriction period across various countries serves as compelling evidence of the effectiveness of NPIs. Finally, the study encounters some methodological limitations. Although the interrupted time‐series analysis accounted for key factors like seasonality and trends, it does not considerx other potential confounders such as climatic conditions, other public health initiatives, changes in healthcare access, or the introduction of influenza vaccinations. However, given the effect size estimated across various countries, we believe these omitted factors likely do not significantly alter our overall findings.

## 4 Conclusion

In summary, our findings support the hypothesis of immunity debt, with increases in influenza rates of 131.72% compared to historical levels in winter seasons and 161.23% in summer seasons, 1 year after the global relaxation of COVID‐19 restrictions. Recognizing the concept of immunity debt is a critical step toward enhancing strategies for responding to future pandemics over the long term. Our research provides empirical evidence of immunity debt and quantifies it on a global scale, underscoring the need to consider immunity debt in the development of future pandemic response strategies.

## 5 Experimental Section

### Study Design and Data Sources

Data was gathered on seasonal influenza from 179 countries or areas using FluNet, a global surveillance platform under the Global Influenza Programme. Launched in 1997, FluNet is a web‐based tool for tracking influenza viruses, including a number of influenza viruses detected by subtype. This crucial data, updated weekly and publicly available, helps monitor the global virus movement and understand epidemiological trends. The temporal scope of the investigation encompassed the years 2012 to 2024 for all countries under study. The choice of 2012 as the inception year was informed by the occurrence of the H1N1 pandemic in 2009. To mitigate the residual effects of the H1N1 pandemic on the present analysis, a 3‐year buffer period post‐pandemic was instituted, thereby establishing 2012 as the baseline year for this research. Within the scope of this research, an exclusion criterion was applied to a total of 59 countries based on the presence of at least 24 consecutive zero values in their datasets, the conclusion of their data series with an undetermined number of zero values, the absence of reported cases prior to the year 2020, or the lack of reported cases following 2020. Additionally, four countries or areas were excluded due to their COVID‐19 stringency index being recorded as zero. As a result of these criteria, 116 countries or areas were deemed eligible for inclusion in the study, as depicted in Figure S1 (Supporting Information). The 116 countries or areas were categorized into six geographical regions according to their continental locations: Africa, Asia, Europe, North America, Oceania, and South America.

The COVID‐19 stringency index data for 255 countries was obtained from the Oxford COVID‐19 Government Response Tracker (OxCGRT).^[^ ^22^ ^]^ This tracker aggregates information on various government policies enacted to combat COVID‐19 over the years 2020, 2021, and 2022. The stringency index is derived from a composite measure encompassing nine response metrics: school closures; workplace closures; cancellation of public events; restrictions on public gatherings; closures of public transport; stay‐at‐home requirements; public information campaigns; restrictions on internal movement; and international travel controls. This index was utilized to evaluate the implementation and easing of COVID‐19 restrictions across different countries. For the subset of 116 countries previously identified for inclusion in this study, specific periods of COVID‐19 restrictions and their subsequent relaxation were delineated using this comprehensive dataset.

### Definition of COVID‐19 Restriction Period and Release Period

In this research, the initiation and conclusion of COVID‐19 restriction and relaxation periods for each country were determined using the COVID‐19 Stringency Index. The onset of a restriction period was identified in the month marked by the greatest absolute monthly change in the daily Stringency Index, provided that the index's value at the month's end exceeded its value at the start. Conversely, the conclusion of a restriction period was indicated by a month showing substantial monthly fluctuation in the Stringency Index, followed by a consistent decrease in the index's values after relaxation measures were introduced. In certain instances, a slight increase in the index value was noted after what was initially considered the end of the restriction period. To precisely determine the end of the restriction period in such cases, a refined approach was adopted, hinging on the condition that the monthly variance of the daily Stringency Index did not exceed a threshold of five. This specific threshold was established based on methodological experimentation and was found to be effective in accurately identifying the end of the restriction period. However, for six countries, the relaxation of restrictions could not be definitively determined using this methodology; therefore, for these countries, the relaxation period was manually identified. The specifics of the restriction and relaxation periods for the 116 countries are detailed in the supplementary materials.

The relaxation of restrictions period was delineated as a 1‐year interval subsequent to the relaxation point, a duration selected to account for the diverse flu seasons observed in different countries. This approach is based on the hypothesis that immunity debt may manifest during the flu season, thus requiring a year‐long period of observation to comprehensively document the occurrence of immunity debt. For each country, the median, mean, and maximum values of the daily Stringency Index during the COVID‐19 restriction period were calculated.

The COVID‐19 restrictions period and relaxation period were identified for all 116 countries, as delineated by the COVID‐19 stringency index. The reduction in case numbers was analyzed during the COVID‐19 restriction period and the increase in number of cases during relaxation period across all 116 countries.

### Statistical Analysis—Model Selection

Influenza incidence data were compiled into weekly aggregates and visually represented according to the ISO week format. This study utilized five distinct time series models to analyze the weekly influenza incidence across 116 countries: an Autoregressive Integrated Moving Average (ARIMA), a Negative Binomial Generalized Linear Model (NB GLM), a historical model, a Prophet, and Quasi‐Poisson Generalized Linear Model (QP GLM). The historical model estimated counterfactual predictions for the periods of COVID‐19 restrictions and relaxation by employing the average weekly values from the historical period of 2012–2019. Conversely, the counterfactual predictions for the remaining models during the restriction and relaxation periods were derived from the models’ forecasts. The choice of models was determined based on the duration of the COVID‐19 restriction and relaxation intervals, necessitating the utilization of a 2‐year timeframe (2018‐2019) for validation objectives, attributable to the fact that these intervals spanned a minimum duration of two years. To address the variability in weekly influenza case numbers among the countries selected, the evaluation of model performance integrated two key indicators: Normalized Mean Squared Error (NMSE) and the ratio of Root Mean Squared Error to Mean (RMSE/Mean). In all examined models, harmonic components, specifically sine and cosine terms, were incorporated to capture annual seasonality through the application of Fourier transformations. Furthermore, the variable of time was integrated into the models to accommodate for the assessment of long‐term trends.

### The Reduction and Immunity Debt

The reduction in influenza case numbers during the COVID‐19 restriction period was calculated by subtracting the observed number of cases within this period from the predicted value obtained through counterfactual inference, followed by dividing the difference by the counterfactual prediction. This percentage change in influenza case numbers is interpreted as the reduction attributable to COVID‐19 restrictions. Recognizing that influenza displays pronounced seasonal patterns and acknowledging the variability of flu seasons across different countries, the reduction was reported for winter seasons and summer seasons. This methodology aligns with findings from prior research and supports our hypothesis regarding the seasonal nature of influenza. The winter season was designated as the first and fourth quarters, while the summer season corresponded to the second and third quarters.

Within the current hypothesis, the observed increase in influenza cases during the reduction period is conceptualized as stemming from two main factors. First, a portion of this surge can be attributed to alterations in behavioral patterns, including enhanced interpersonal interactions and a reversion to the social distancing norms observed prior to the COVID‐19 pandemic. Second, the concept of immunity debt plays a significant role. To quantify the increase attributable to changes in behavior patterns, modeling techniques were employed under the assumption that lifting behavioral restrictions would revert social behaviors to their pre‐pandemic state, thereby restoring influenza epidemics to their historical baselines. Thus, modeling was utilized to estimate the theoretical level of influenza activity in the absence of the pandemic.

The reduction and immunity debt were quantified and presented as a percentage within a 95% confidence interval (95% CI), which may be interpreted as the percentage change in the number of influenza cases over a specified period. The confidence interval pertaining to the reduction and immunity debt within the historical model was determined through the application of Nonparametric Bootstrap Confidence Intervals, executed iteratively a thousand times.

### The Association Between Reduction and Immunity Debt and Stringency Index

The relationship between reduction and immunity debt and the stringency index was evaluated utilizing a linear model. For the analysis of reduction, countries that demonstrated a significant decrease during the COVID‐19 restriction period were selected. Subsequently, a linear model was employed to examine the correlation between the reduction and various measures of the stringency index (median, mean, and maximum) throughout the COVID‐19 restriction period, with separate analyses conducted for winter seasons (quarter 14) and summer seasons (quarter 23). Regarding immunity debt, countries that demonstrated significant immunity debt were selected to explore the association between the stringency index (encompassing median, mean, and maximum values) during the COVID‐19 restriction period and the observed immunity debt.

### Global Reduction and Immunity Debt

To ascertain the worldwide magnitude of reduction and immunity debt associated with COVID‐19 restrictions, the population figures of the 116 selected countries were employed as weighting factors. This methodology facilitated the aggregation of global data concerning the reduction and immunity debt attributable to COVID‐19 restriction measures.

Subgroup analysis was conducted, with stratification based on the subtype of influenza (influenza A and influenza B).

### Replication of the Association

A two‐step validation process was employed to verify the association between the reduction/increase in hospital visits and the stringency index. This validation utilized patient‐level data from The Health Improvement Network (THIN), a large European database network of anonymized Electronic Health Records, encompassing seven countries: France, the United Kingdom, Spain, Italy, Belgium, and Romania. Data from Spain was not included in this study. In the second step of validation, the presence of immunity debt was assessed in other chronic diseases (hypertension) and other respiratory diseases (chronic obstructive pulmonary disease (COPD)). Additionally, the association was examined between the stringency index and the reduction/increase in hospital visits for these diseases. Considering that the patient‐level data spans from 2018 to 2023, and the FluNet data spans from 2008 to 2024, the FluNet data was selected corresponding to the same time range and countries as the patient‐level data to test the association. A linear model was employed to test the direction of the association (positive or negative) and the correlation coefficient to evaluate the strength of the relationship.

All analytical procedures were conducted utilizing the R software, version 4.3.1. The analyses employed several packages within this environment, namely MASS, forecast, prophet, boots, natural earth, and ggplot2, to facilitate the comprehensive examination and modeling of the data.

## Conflict of Interest

The authors declare no conflict of interest.

## Supporting information



Supporting Information

## Data Availability

The data that support the findings of this study are openly available in Global Influenza Programme at https://www.who.int/tools/flunet, reference number 0.
