# Supplementary material for: Immunity Debt for Seasonal Influenza After the COVID‐19 Pandemic and as a Result of Nonpharmaceutical Interventions: An Ecological Analysis and Cohort Study
Source: Adv Sci (Weinh). 2025 Apr 5;12(20):2410513. doi: 10.1002/advs.202410513 (PMC12120713; doi:10.1002/advs.202410513)
Supplement: Supplementary file 1 — Supporting Information [file ADVS-12-2410513-s001.docx]

**Immunity debt for seasonal influenza after the COVID-19 pandemic and as a result of non-pharmaceutical interventions: an ecological analysis and cohort study**

*Li Chen ^1,2^; Yuchen Guo ^1^; Kim López-Güell ^1^; Jun Ma ^2^; Yanhui Dong ^2*^; Junqing Xie ^1#^; and* ***Daniel Prieto Alhambra ^1,3^****^#^*

1: Centre for Statistics in Medicine and NIHR Biomedical Research Centre Oxford, NDORMS, University of Oxford, Oxford, UK

2: Institute of Child and Adolescent Health, School of Public Health, Peking University

3: Department of Medical Informatics, Erasmus University Medical Center, Rotterdam, the Netherlands

***Correspondence:**

**Yanhui Dong,** Institute of Child and Adolescent Health, School of Public Health, Peking University; Email: dongyanhui@bjmu.edu.cn;

# Junqing Xie and **Daniel Prieto Alhambra**, Joint senior author.

**Keywords:** Immunity debt, influenza, COVID-19, nonpharmaceutical interventions

**supplementary text**

The results of reduction percentage during the restriction period

**Reduction during COVID-19 restriction period**

Overall, among the 116 countries analyzed, 87 showed a significant reduction in influenza cases during winter seasons, whereas 101 countries reported a decrease during Quarter 23. In winter seasons, there was a notable weighted decrease in influenza cases by 46.3% (95% CI: -70.78% to -15.79%) relative to the counterfactual predictions for the period coinciding with COVID-19 restrictions. This weighted reduction diminished to 16.63% (95% CI: -53.83% to 45.27%) by summer seasons. Notably, the largest decline during winter seasons was observed in Europe, with a decrease of 71.68% (95% CI: -89.13% to -48.41%) (**Table S3 and Figure S25**). Conversely, in South America, there was an increase in the number of cases during the same quarter. In summer seasons, the number of cases in South America significantly decreased, aligning with the typical influenza season in the region, which spans from April to November. This indicates that the reductions predominantly occurred during the influenza season in each country, suggesting a temporal correlation between the seasonality of influenza and the observed impacts of COVID-19 restrictions on influenza transmission.

For influenza A, the number of cases decreased in 80 of the 116 countries during the winter seasons of COVID-19 restrictions period, with a global reduction of 46.76% (95%CI: -74.56% to -9.62%). During the summer seasons of restriction period, 97 countries reported a decrease, with a global reduction of 8.80% (95%CI: -52.41% to 57.25%). In South America, the substantial decrease occurred primarily in the summer seasons, with a reduction of 92.91% (95%CI: -96.70% to -86.91%) (**Table S4 and Figure S26**). For influenza tB, while the global reduction was not pronounced, significant decreases were observed in specific regions, including South America, North America, and Europe (**Table S5 and Figure S27**).

**Association between stringency index and reduction during COVID-19 restriction period**

The country-specific reduction in influenza cases demonstrated a positive linear relationship with the median, mean, and maximum stringency index during winter seasons, with significance observed only for the mean value (p = 0.0267). In summer seasons, a positive association with both median and mean stringency indices was also noted (**Figure S30**). Conversely, for the maximum value, a weak negative relationship was observed, though it did not reach statistical significance. Furthermore, the relationship between the reduction in influenza subtypes and both mean and median stringency indices showed a positive association in both winter seasons and summer seasons (**Figure S31**). However, no significant association was observed between the stringency index and the reduction in specific influenza subtypes. In summer seasons, the relationship between the stringency index and overall reduction turned negative (**Figure S32**).

**Replication of association**

In The Health Improvement Network (THIN) data (patient-level), we used a consistent process to calculate the reduction in daily influenza cases during the restriction period and the increase during the relaxation period. We observed a positive association between the stringency index and the reduction in daily influenza cases (**Figure S38**).

Table S1. The number of influenza cases and the dates of restrictions and relaxations for 116 countries.

| Continent | country name | influenza | influenza A | influenza B | Restriction | Relaxation |
| --- | --- | --- | --- | --- | --- | --- |
| Africa | Burkina Faso | 754 | 546 | 208 | 2020/3/1 | 2021/8/1 |
|  | Central African Republic | 809 | 506 | 303 | 2020/3/1 | 2022/6/1 |
|  | Côte d'Ivoire | 2868 | 1866 | 1002 | 2020/3/1 | 2022/4/1 |
|  | Cameroon | 3353 | 2344 | 1009 | 2020/3/1 | 2022/8/1 |
|  | Democratic Republic of the Congo | 1202 | 799 | 403 | 2020/3/1 | 2022/9/1 |
|  | Algeria | 2059 | 1538 | 521 | 2020/3/1 | 2022/9/1 |
|  | Egypt | 15445 | 10676 | 4769 | 2020/3/1 | 2022/12/1 |
|  | Ethiopia | 2050 | 1381 | 669 | 2020/3/1 | 2022/10/1 |
|  | Ghana | 5046 | 3609 | 1437 | 2020/3/1 | 2022/4/1 |
|  | Kenya | 2306 | 1692 | 614 | 2020/3/1 | 2022/10/1 |
|  | Morocco | 2484 | 1770 | 714 | 2020/3/1 | 2021/11/1 |
|  | Madagascar | 4594 | 2545 | 2049 | 2020/3/1 | 2022/5/1 |
|  | Mauritius | 1154 | 1004 | 150 | 2020/3/1 | 2022/4/1 |
|  | Niger | 850 | 575 | 275 | 2020/3/1 | 2022/1/1 |
|  | Nigeria | 884 | 578 | 306 | 2020/3/1 | 2022/4/1 |
|  | Rwanda | 483 | 398 | 85 | 2020/3/1 | 2022/9/1 |
|  | Senegal | 5131 | 3563 | 1568 | 2020/3/1 | 2021/3/1 |
|  | Togo | 2172 | 1474 | 698 | 2020/3/1 | 2022/10/1 |
|  | Tunisia | 2455 | 1975 | 480 | 2020/3/1 | 2022/2/1 |
|  | United Republic of Tanzania | 2225 | 1578 | 647 | 2020/3/1 | 2022/6/1 |
|  | Uganda | 2952 | 2163 | 789 | 2020/3/1 | 2022/5/1 |
|  | South Africa | 9736 | 7211 | 2525 | 2020/3/1 | 2022/6/1 |
|  | Zambia | 1938 | 1156 | 782 | 2020/3/1 | 2022/1/1 |
| Asia | Bangladesh | 7881 | 5242 | 2639 | 2020/3/1 | 2022/2/1 |
|  | Bahrain | 1614 | 1261 | 353 | 2020/3/1 | 2022/2/1 |
|  | Bhutan | 3002 | 2018 | 984 | 2020/3/1 | 2022/5/1 |
|  | China | 571774 | 351954 | 219820 | 2020/1/1 | 2022/12/1 |
|  | China, Hong Kong SAR | 150380 | 114182 | 36198 | 2020/2/1 | 2022/12/1 |
|  | Indonesia | 8834 | 5531 | 3303 | 2020/4/1 | 2022/12/1 |
|  | India | 32580 | 26608 | 5972 | 2020/3/1 | 2022/4/1 |
|  | Iran (Islamic Republic of) | 21714 | 18965 | 2749 | 2020/3/1 | 2022/6/1 |
|  | Iraq | 2963 | 2497 | 466 | 2020/2/1 | 2022/12/1 |
|  | Israel | 12716 | 9494 | 3222 | 2020/3/1 | 2022/2/1 |
|  | Jordan | 2794 | 2383 | 411 | 2020/3/1 | 2022/10/1 |
|  | Japan | 70007 | 51485 | 18522 | 2020/2/1 | 2022/6/1 |
|  | Kazakhstan | 6100 | 4643 | 1457 | 2020/3/1 | 2022/5/1 |
|  | Kyrgyzstan | 1556 | 965 | 591 | 2020/3/1 | 2022/9/1 |
|  | Cambodia | 2826 | 1945 | 881 | 2020/3/1 | 2022/12/1 |
|  | Republic of Korea | 16175 | 9885 | 6290 | 2020/4/1 | 2022/4/1 |
|  | Lao People's Democratic Republic | 4501 | 2846 | 1655 | 2020/3/1 | 2022/5/1 |
|  | Sri Lanka | 8485 | 6219 | 2266 | 2020/3/1 | 2022/3/1 |
|  | Mongolia | 5228 | 3852 | 1376 | 2020/1/1 | 2022/2/1 |
|  | Malaysia | 3828 | 2588 | 1240 | 2020/3/1 | 2022/5/1 |
|  | Nepal | 13220 | 9137 | 4083 | 2020/3/1 | 2022/2/1 |
|  | Oman | 11039 | 8324 | 2715 | 2020/3/1 | 2022/5/1 |
|  | Pakistan | 3616 | 3012 | 604 | 2020/3/1 | 2022/3/1 |
|  | Philippines | 3800 | 2150 | 1650 | 2020/3/1 | 2021/11/1 |
|  | Qatar | 44961 | 33423 | 11538 | 2020/3/1 | 2022/4/1 |
|  | Russian Federation | 161390 | 125940 | 35450 | 2020/3/1 | 2022/11/1 |
|  | Singapore | 8339 | 5640 | 2699 | 2020/4/1 | 2022/4/1 |
|  | Thailand | 7818 | 4847 | 2971 | 2020/3/1 | 2022/6/1 |
|  | Türkiye | 22485 | 17381 | 5104 | 2020/3/1 | 2022/3/1 |
|  | Uzbekistan | 498 | 316 | 182 | 2020/3/1 | 2022/4/1 |
|  | Viet Nam | 5172 | 3367 | 1805 | 2020/3/1 | 2022/4/1 |
| Europe | Albania | 2987 | 2368 | 619 | 2020/3/1 | 2022/5/1 |
|  | Austria | 26166 | 18356 | 7810 | 2020/3/1 | 2022/3/1 |
|  | Azerbaijan | 949 | 460 | 489 | 2020/3/1 | 2022/6/1 |
|  | Belgium | 4040 | 2976 | 1064 | 2020/3/1 | 2022/3/1 |
|  | Bulgaria | 3442 | 2562 | 880 | 2020/3/1 | 2022/3/1 |
|  | Belarus | 5462 | 4735 | 727 | 2020/4/1 | 2022/11/1 |
|  | Switzerland | 65117 | 47682 | 17435 | 2020/3/1 | 2022/2/1 |
|  | Czechia | 4149 | 2976 | 1173 | 2020/3/1 | 2022/3/1 |
|  | Germany | 12447 | 8691 | 3756 | 2020/3/1 | 2022/4/1 |
|  | Denmark | 54838 | 36866 | 17972 | 2020/3/1 | 2022/3/1 |
|  | Spain | 104776 | 80406 | 24370 | 2020/3/1 | 2022/8/1 |
|  | Estonia | 10182 | 7515 | 2667 | 2020/3/1 | 2022/3/1 |
|  | Finland | 1729 | 1388 | 341 | 2020/3/1 | 2022/2/1 |
|  | France | 143491 | 105685 | 37806 | 2020/3/1 | 2022/2/1 |
|  | United Kingdom | 170974 | 129898 | 41076 | 2020/3/1 | 2022/4/1 |
|  | Georgia | 1524 | 1163 | 361 | 2020/3/1 | 2022/3/1 |
|  | Greece | 7398 | 5756 | 1642 | 2020/3/1 | 2022/2/1 |
|  | Croatia | 17067 | 14217 | 2850 | 2020/3/1 | 2022/4/1 |
|  | Hungary | 5554 | 3987 | 1567 | 2020/3/1 | 2022/3/1 |
|  | Ireland | 21365 | 15295 | 6070 | 2020/3/1 | 2022/5/1 |
|  | Iceland | 3174 | 2360 | 814 | 2020/3/1 | 2022/2/1 |
|  | Italy | 32122 | 23091 | 9031 | 2020/2/1 | 2022/2/1 |
|  | Lithuania | 6796 | 5303 | 1493 | 2020/3/1 | 2022/2/1 |
|  | Luxembourg | 3921 | 2513 | 1408 | 2020/3/1 | 2022/3/1 |
|  | Latvia | 17320 | 12540 | 4780 | 2020/3/1 | 2022/3/1 |
|  | Republic of Moldova | 2131 | 1622 | 509 | 2020/3/1 | 2022/3/1 |
|  | Malta | 3640 | 2491 | 1149 | 2020/3/1 | 2022/4/1 |
|  | Netherlands (Kingdom of the) | 25921 | 21026 | 4895 | 2020/3/1 | 2022/1/1 |
|  | Norway | 149320 | 100442 | 48878 | 2020/3/1 | 2022/2/1 |
|  | Poland | 12750 | 9611 | 3139 | 2020/3/1 | 2022/2/1 |
|  | Portugal | 22722 | 17083 | 5639 | 2020/3/1 | 2022/2/1 |
|  | Romania | 11058 | 7626 | 3432 | 2020/3/1 | 2022/3/1 |
|  | Serbia | 6270 | 4948 | 1322 | 2020/3/1 | 2022/4/1 |
|  | Slovakia | 2930 | 1772 | 1158 | 2020/3/1 | 2022/3/1 |
|  | Slovenia | 24621 | 17820 | 6801 | 2020/3/1 | 2022/2/1 |
|  | Sweden | 104753 | 77344 | 27409 | 2020/3/1 | 2022/2/1 |
|  | Ukraine | 9718 | 7475 | 2243 | 2020/3/1 | 2022/3/1 |
| North America | Canada | 359135 | 263213 | 95922 | 2020/3/1 | 2022/4/1 |
|  | Costa Rica | 3561 | 2777 | 784 | 2020/3/1 | 2022/1/1 |
|  | Cuba | 3594 | 2601 | 993 | 2020/3/1 | 2021/11/1 |
|  | Dominican Republic | 1822 | 1312 | 510 | 2020/3/1 | 2022/2/1 |
|  | Guatemala | 2123 | 1670 | 453 | 2020/3/1 | 2022/7/1 |
|  | Honduras | 1534 | 1083 | 451 | 2020/3/1 | 2022/1/1 |
|  | Jamaica | 1193 | 911 | 282 | 2020/3/1 | 2022/3/1 |
|  | Mexico | 55292 | 44734 | 10558 | 2020/3/1 | 2022/8/1 |
|  | Nicaragua | 6574 | 4317 | 2257 | 2020/4/1 | 2021/6/1 |
|  | Panama | 2337 | 1904 | 433 | 2020/3/1 | 2021/10/1 |
|  | El Salvador | 1782 | 1321 | 461 | 2020/3/1 | 2021/5/1 |
|  | United States of America | 1543247 | 1155659 | 387588 | 2020/3/1 | 2022/8/1 |
| Oceania | Australia | 51964 | 40778 | 11186 | 2020/3/1 | 2022/5/1 |
|  | Fiji | 936 | 628 | 308 | 2020/3/1 | 2022/11/1 |
|  | New Zealand | 15691 | 10568 | 5123 | 2020/3/1 | 2022/4/1 |
| South America | Argentina | 39734 | 32810 | 6924 | 2020/3/1 | 2022/1/1 |
|  | Bolivia (Plurinational State of) | 8885 | 6852 | 2033 | 2020/3/1 | 2021/11/1 |
|  | Brazil | 30981 | 24773 | 6208 | 2020/3/1 | 2022/1/1 |
|  | Chile | 26658 | 19569 | 7089 | 2020/3/1 | 2021/7/1 |
|  | Colombia | 7360 | 6190 | 1170 | 2020/3/1 | 2022/5/1 |
|  | Ecuador | 5114 | 4341 | 773 | 2020/3/1 | 2022/10/1 |
|  | Peru | 8450 | 7167 | 1283 | 2020/3/1 | 2022/10/1 |
|  | Paraguay | 8681 | 6557 | 2124 | 2020/3/1 | 2022/5/1 |
|  | Uruguay | 841 | 656 | 185 | 2020/3/1 | 2022/3/1 |
|  | Venezuela (Bolivarian Republic of) | 521 | 374 | 147 | 2020/3/1 | 2022/5/1 |

Table S2. The summary of stringency index during COVID-19 restriction period

| Continents | Number of countries | Mean of Stringency Index | Mean of Median Stringency Index | Mean of Maximum Stringency Index |
| --- | --- | --- | --- | --- |
| **Overall** | **116** | **55** | **55** | **84** |
| Africa | 23 | 44 | 46 | 81 |
| Asia | 31 | 58 | 58 | 85 |
| Europe | 37 | 53 | 53 | 82 |
| North America | 12 | 65 | 64 | 83 |
| Oceania | 3 | 46 | 55 | 88 |
| South America | 10 | 70 | 68 | 92 |


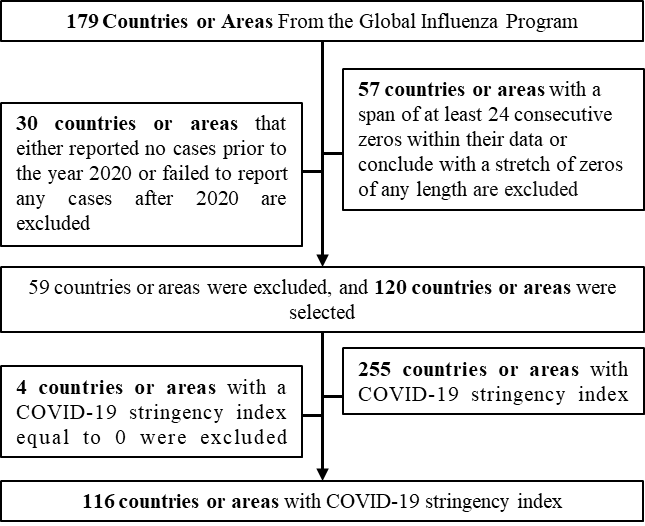


Figure S1. Flow of Country Selection


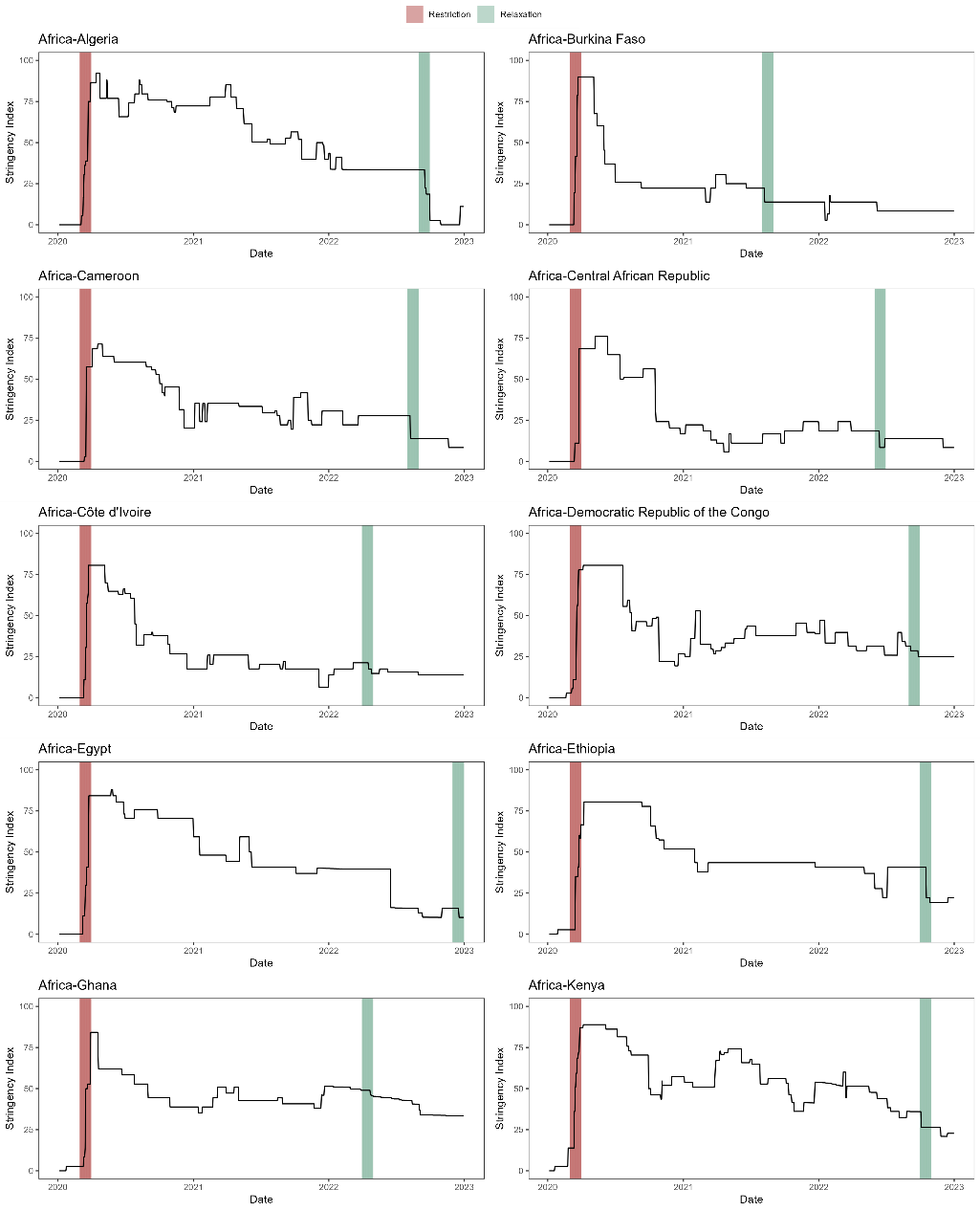


Figure S2. COVID-19 stringency index, initiation and conclusion of restrictions Algeria, Burkina Faso, Cameroon, Central African Republic, Côte d'Ivoire, Democratic Republic of the Congo, Egypt, Ethiopia, Ghana, and Kenya.


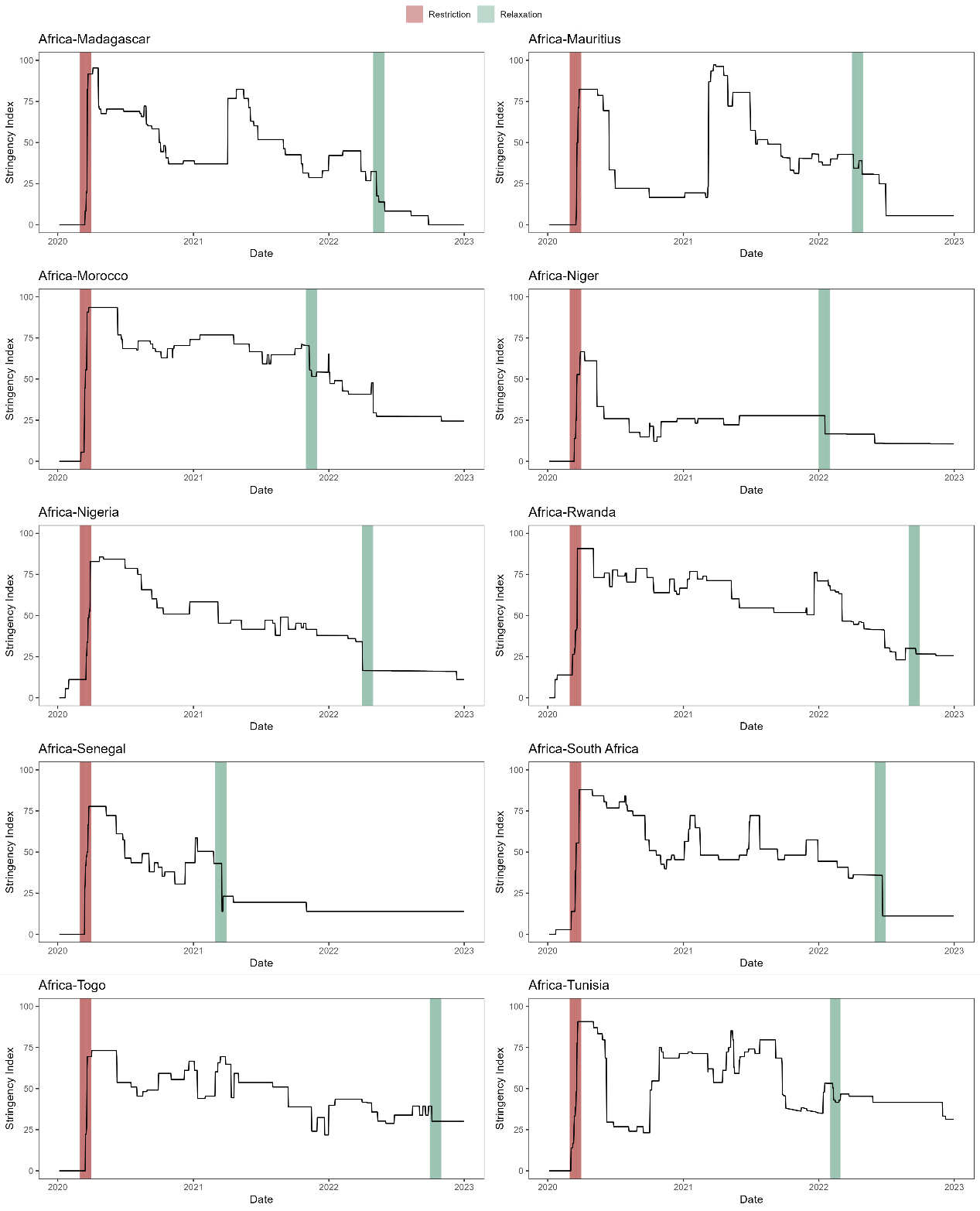


Figure S3. COVID-19 stringency index, initiation and conclusion of restrictions of Madagascar, Mauritius, Morocco ,Niger, Nigeria, Rwanda, Senegal, South Africa, Togo, and Tunisia.


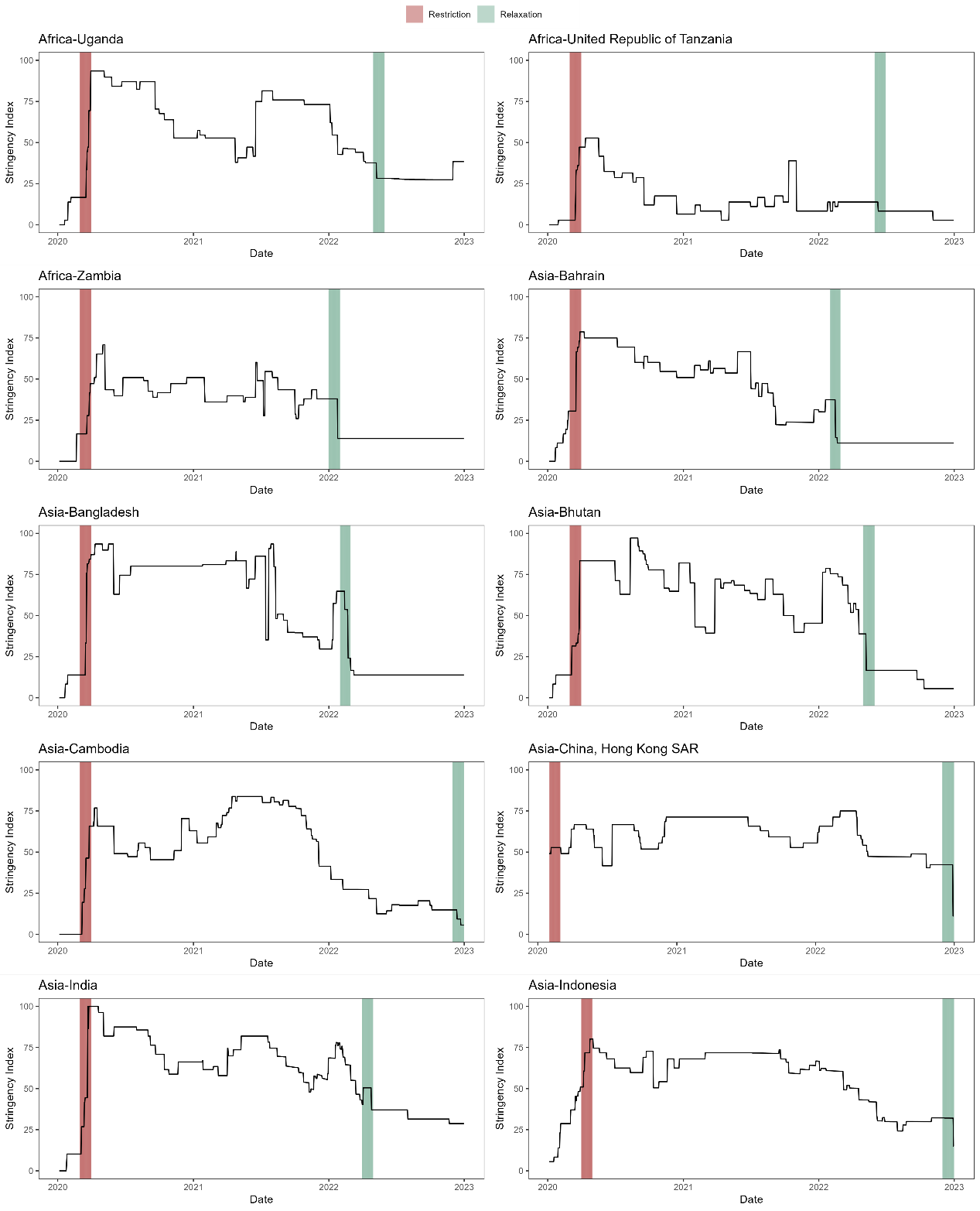


Figure S4. COVID-19 stringency index, initiation and conclusion of restrictions of Uganda, United Republic of Tanzania, Zambia, Bahrain, Bangladesh, Bhutan, Cambodia, China, Hong Kong SAR ,India, Indonesia.


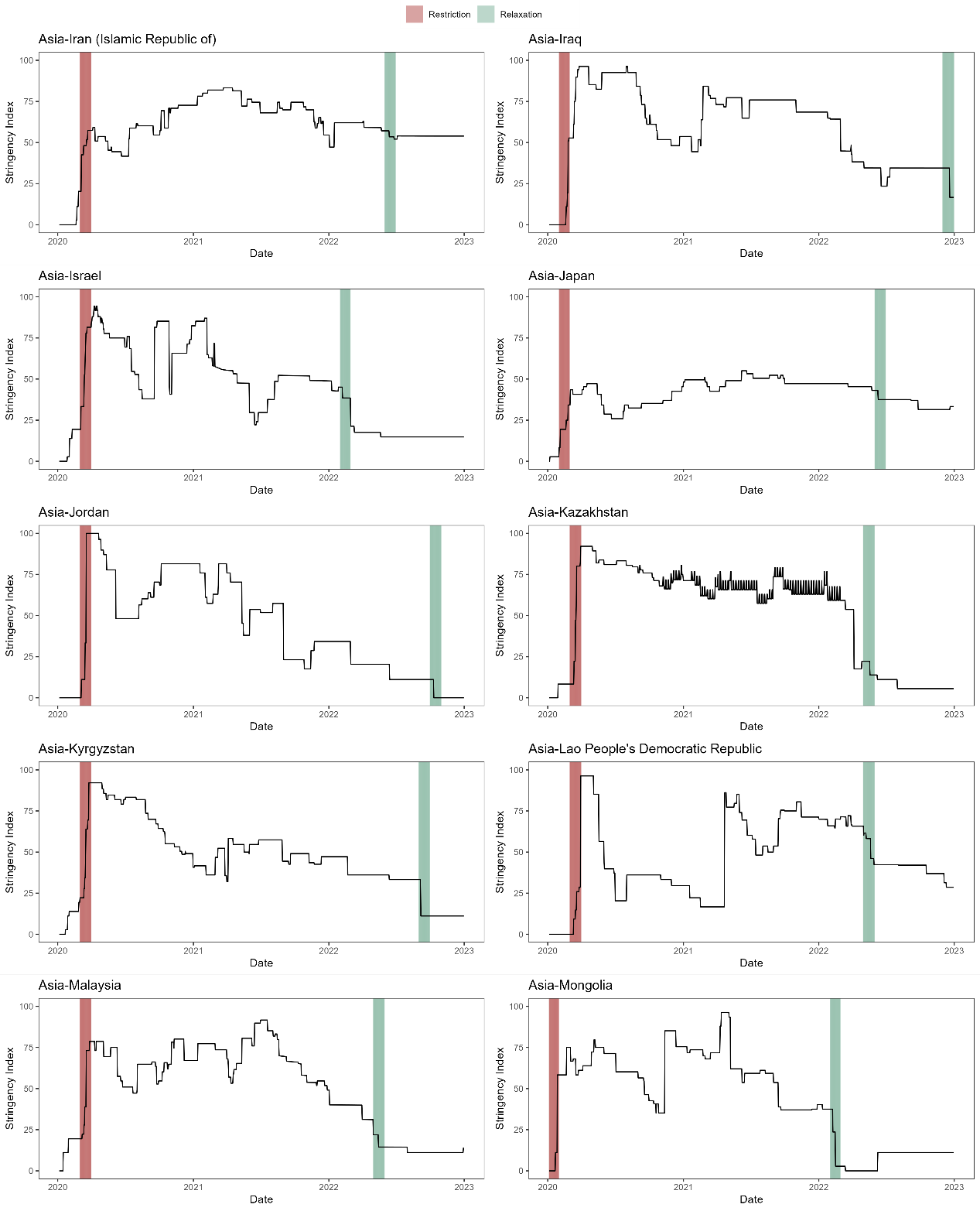


Figure S5. COVID-19 stringency index, initiation and conclusion of restrictions of Iran (Islamic Republic of), Iraq, Israel, Japan, Jordan, Kazakhstan, Kyrgyzstan, Lao People's Democratic Republic, Malaysia, and Mongolia.


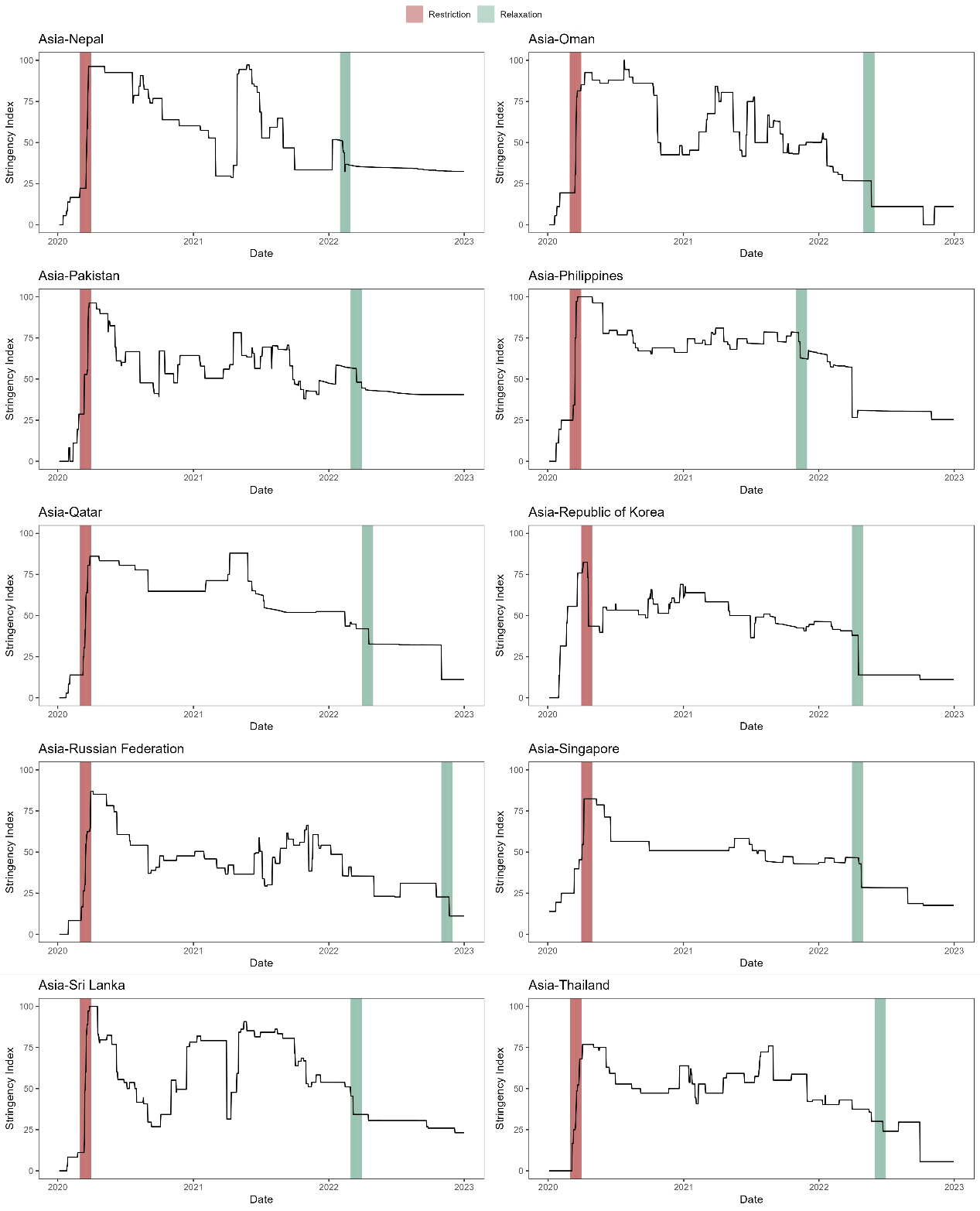


Figure S6. COVID-19 stringency index, initiation and conclusion of restrictions of Nepal, Oman, Pakistan, Philippines, Qatar, Republic of Korea, Russian Federation, Singapore, Sri Lanka, and Thailand.


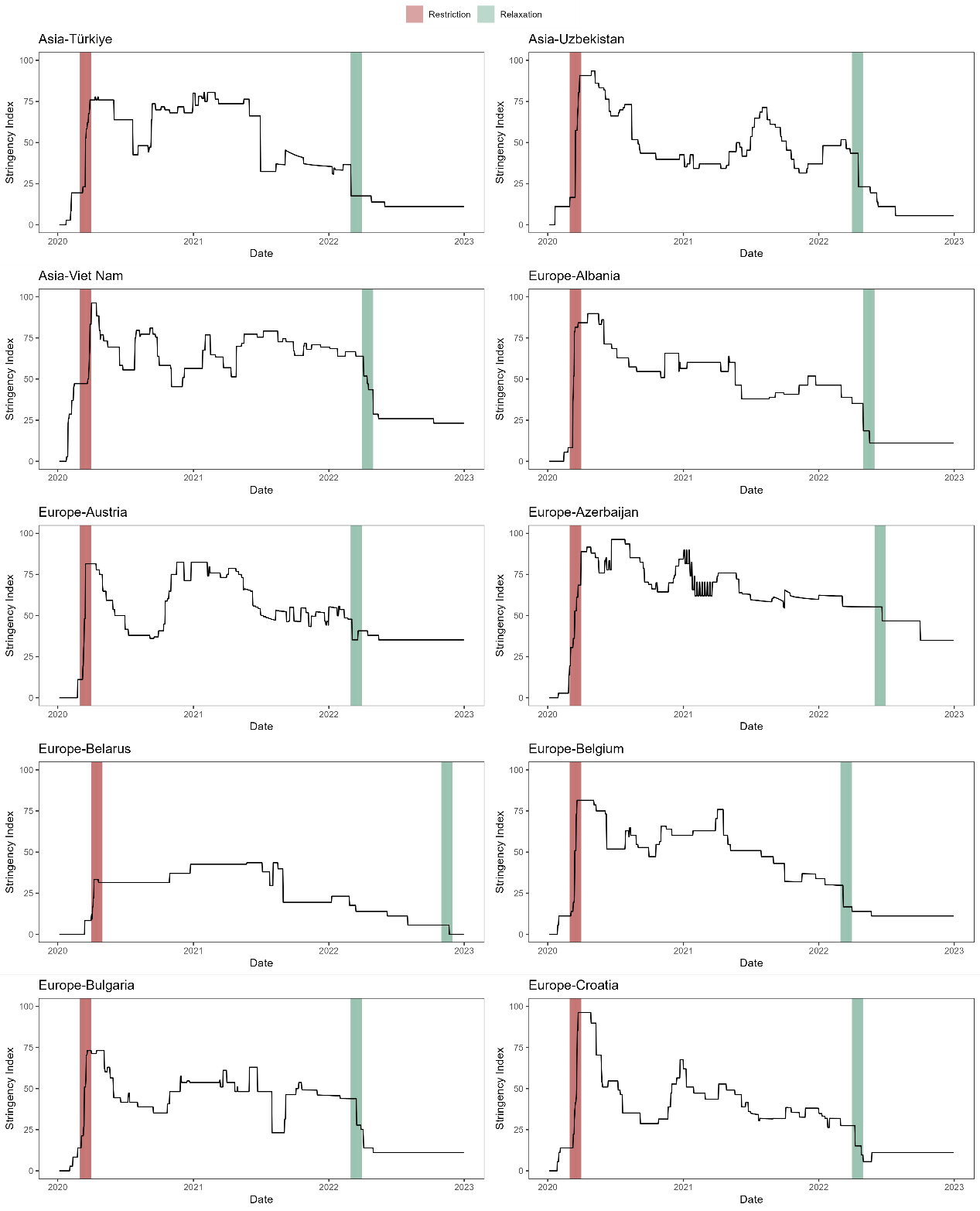


Figure S7. COVID-19 stringency index, initiation and conclusion of restrictions of Türkiye, Uzbekistan, Viet Nam, Albania, Austria, Azerbaijan, Belarus, Belgium, Bulgaria, and Croatia.


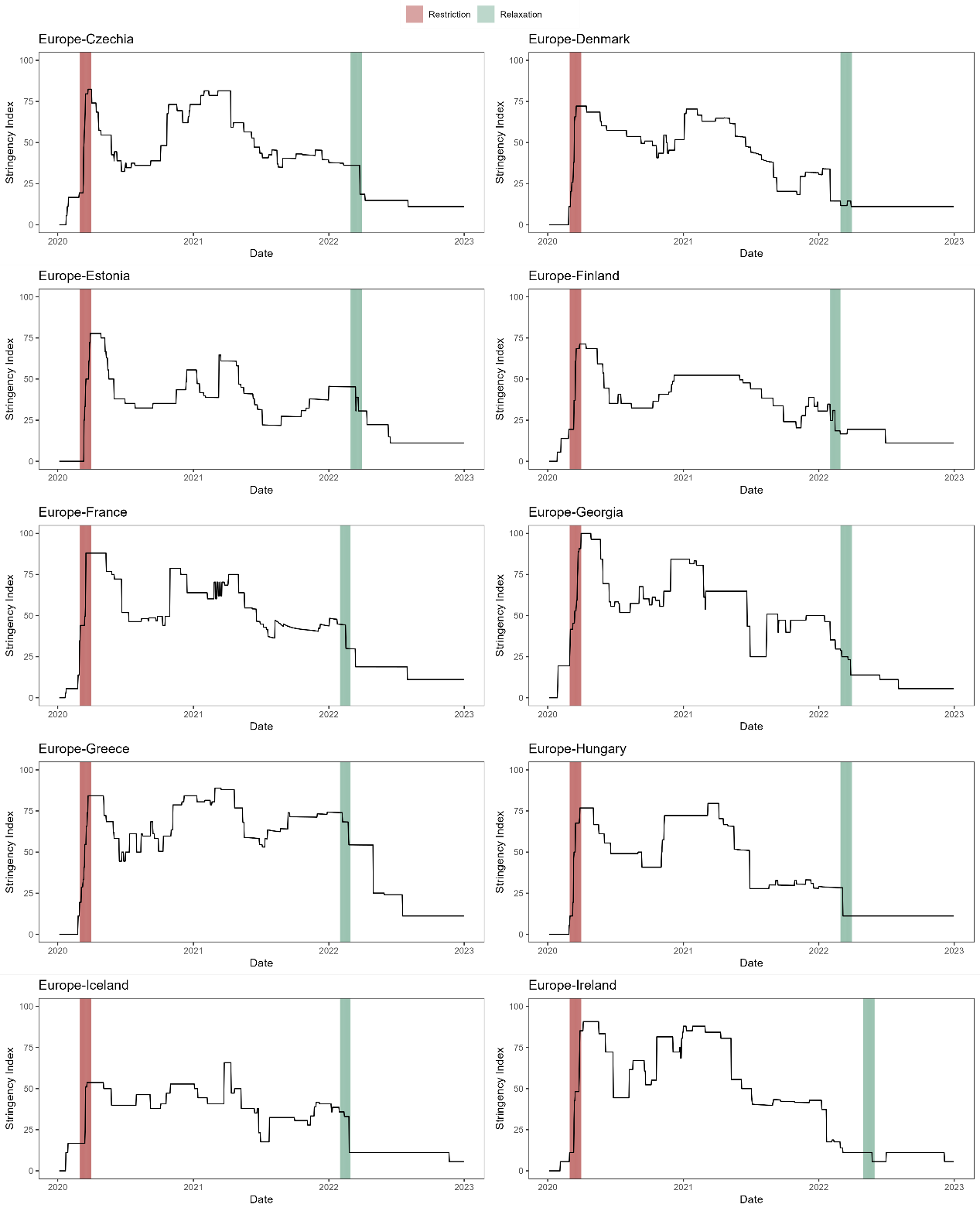


Figure S8. COVID-19 stringency index, initiation and conclusion of restrictions of Czechia, Denmark, Estonia, Finland, France, Georgia, Greece, Hungary, Iceland, and Ireland.


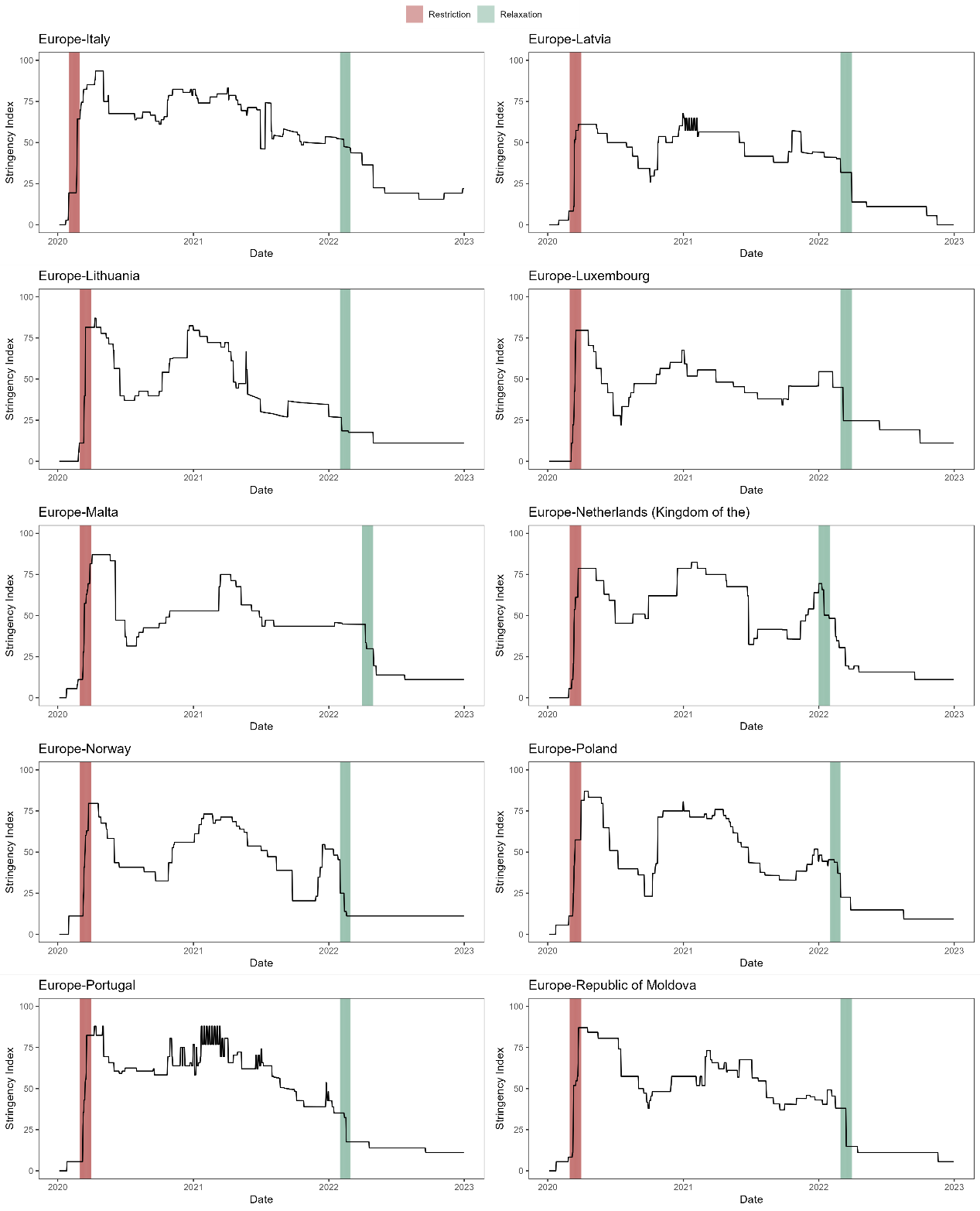


Figure S9. COVID-19 stringency index, initiation and conclusion of restrictions of Italy, Latvia, Lithuania, Luxembourg, Malta, Netherlands (Kingdom of the), Norway, Poland, Portugal, and Republic of Moldova.


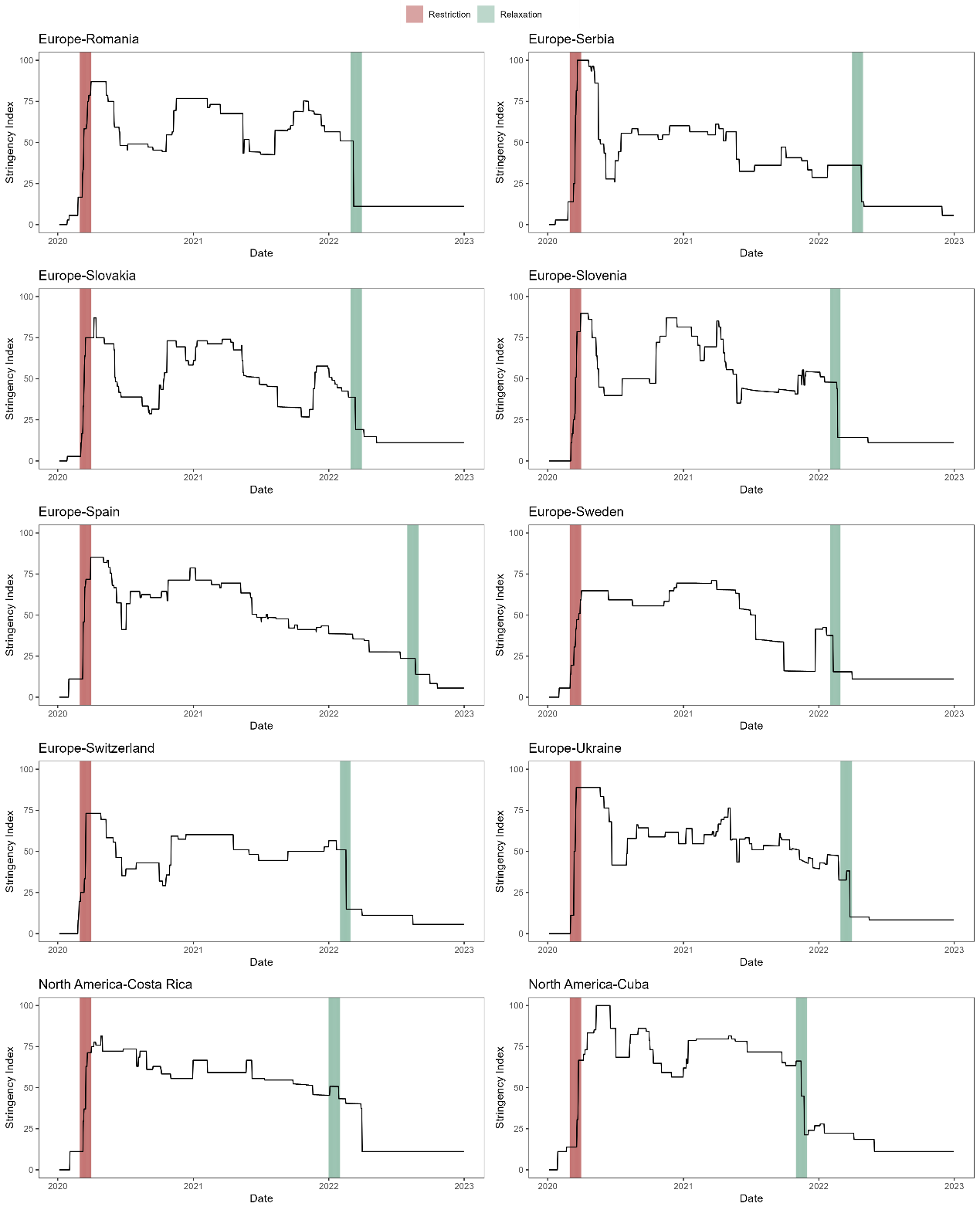


Figure S10. COVID-19 stringency index, initiation and conclusion of restrictions of Romania, Serbia, Slovakia, Slovenia, Spain, Sweden, Switzerland, Ukraine, Costa Rica, and Cuba.


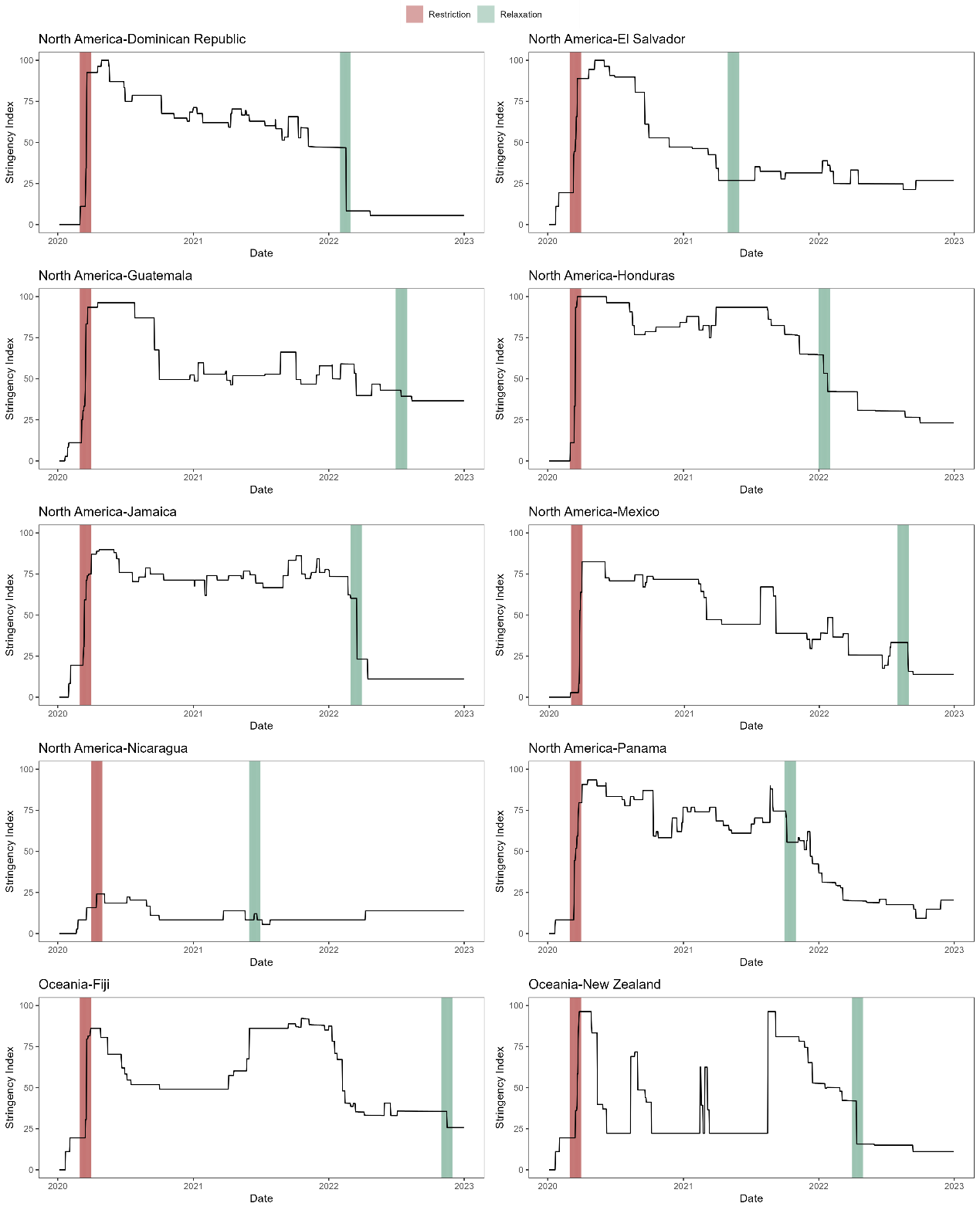


Figure S11. COVID-19 stringency index, initiation and conclusion of restrictions of Dominican Republic, El Salvador, Guatemala, Honduras, Jamaica, Mexico, Nicaragua, Panama, Fiji, and New Zealand.


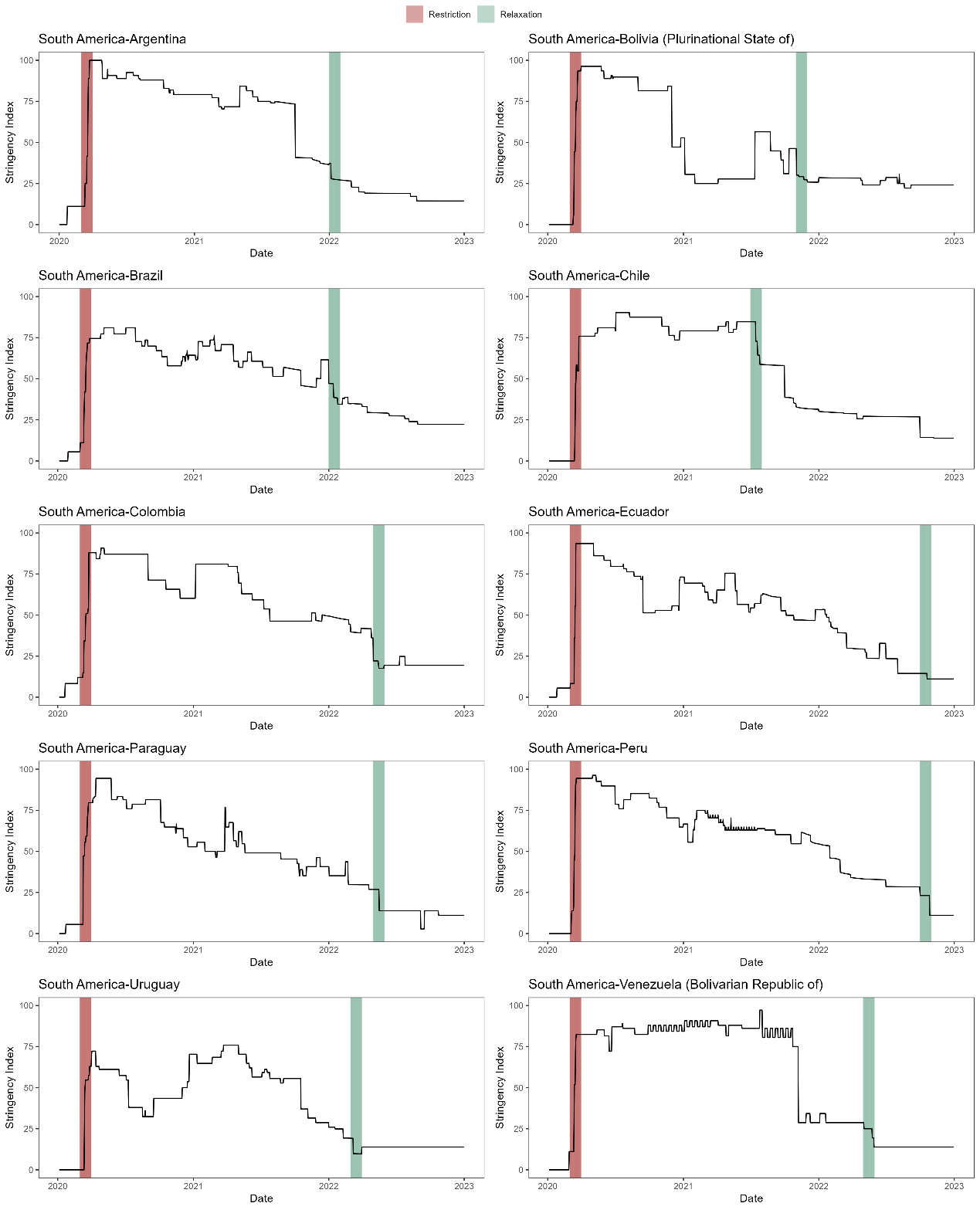


Figure S12. COVID-19 stringency index, initiation and conclusion of restrictions of Argentina, Bolivia (Plurinational State of), Brazil, Chile, Colombia, Ecuador, Paraguay, Peru, Uruguay, and Venezuela (Bolivarian Republic of).


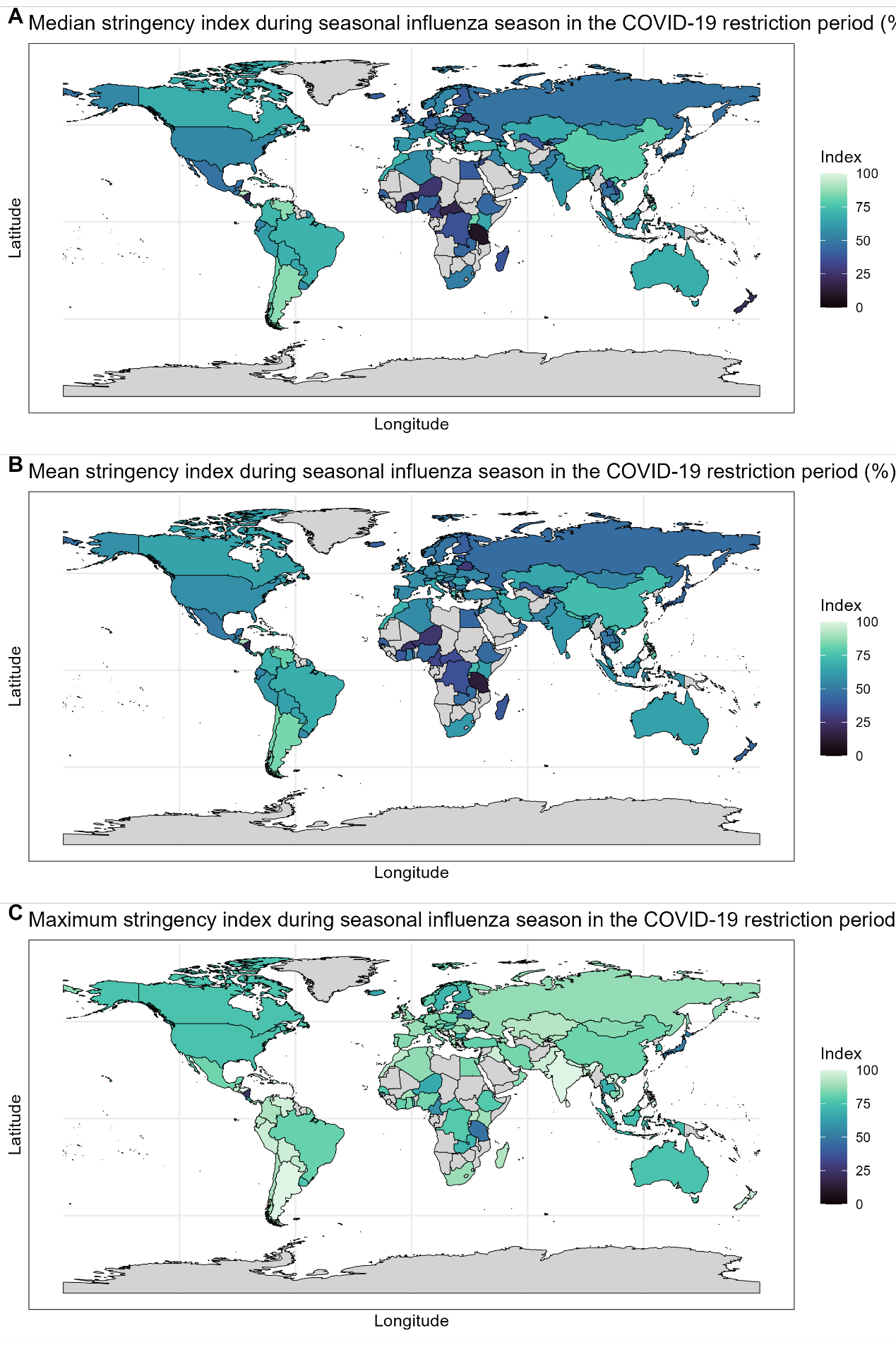


Figure S13. Median, mean, and maximum stringency index by countries.


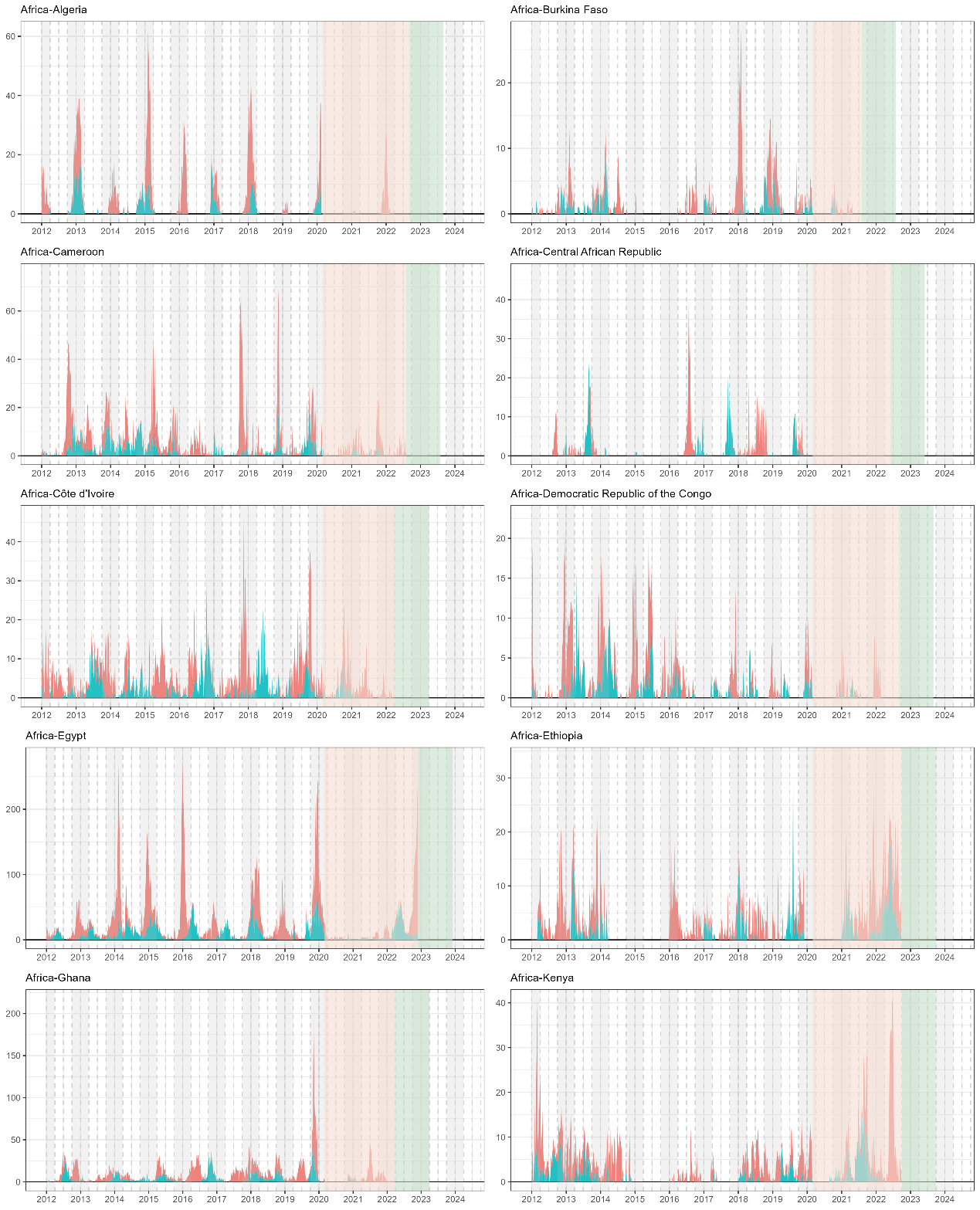


Figure S14. total influenza cases of Algeria, Burkina Faso, Cameroon, Central African Republic, Côte d'Ivoire, Democratic Republic of the Congo, Egypt, Ethiopia, Ghana, and Kenya.


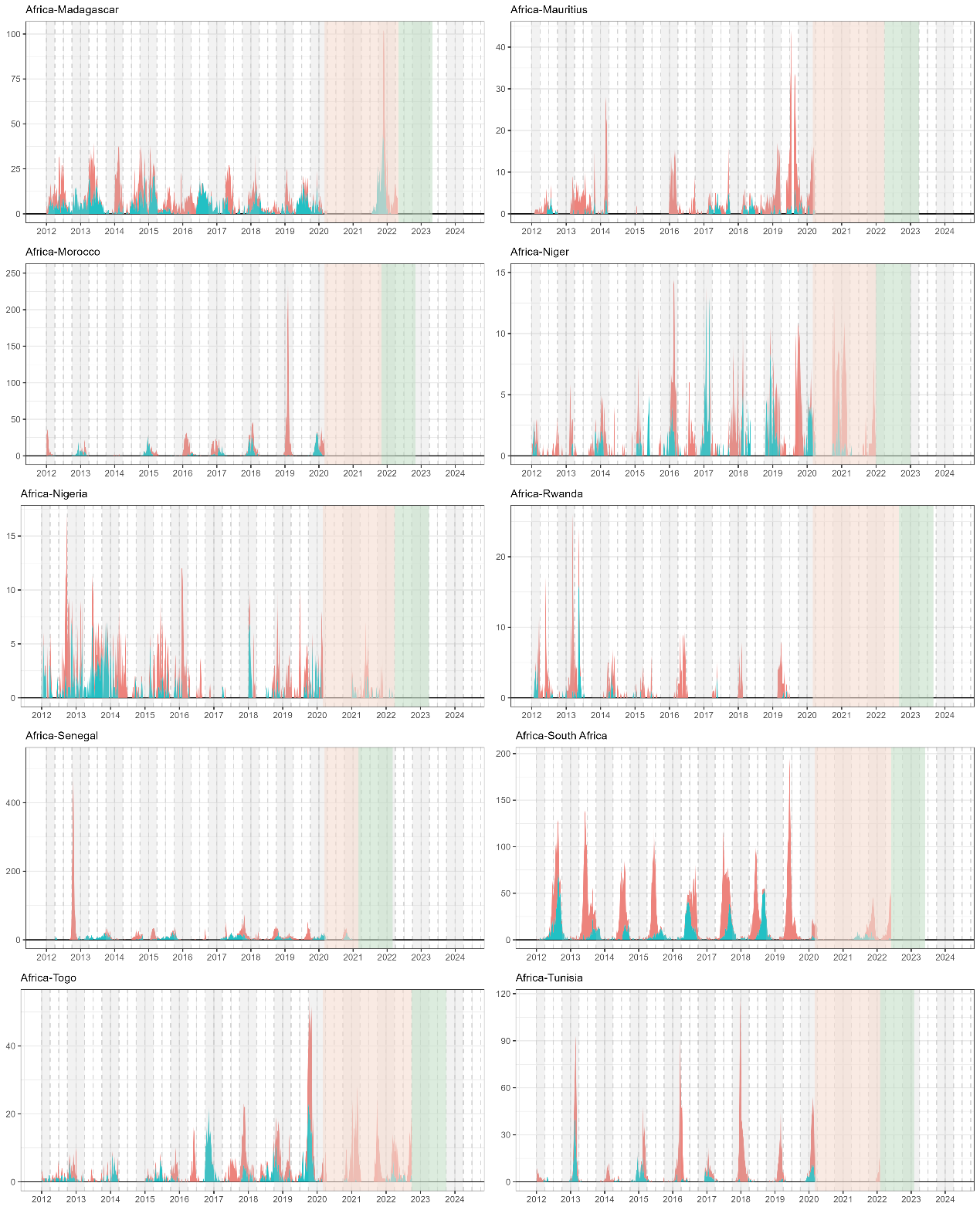


Figure S15. total influenza cases of Madagascar, Mauritius, Morocco ,Niger, Nigeria, Rwanda, Senegal, South Africa, Togo, and Tunisia.


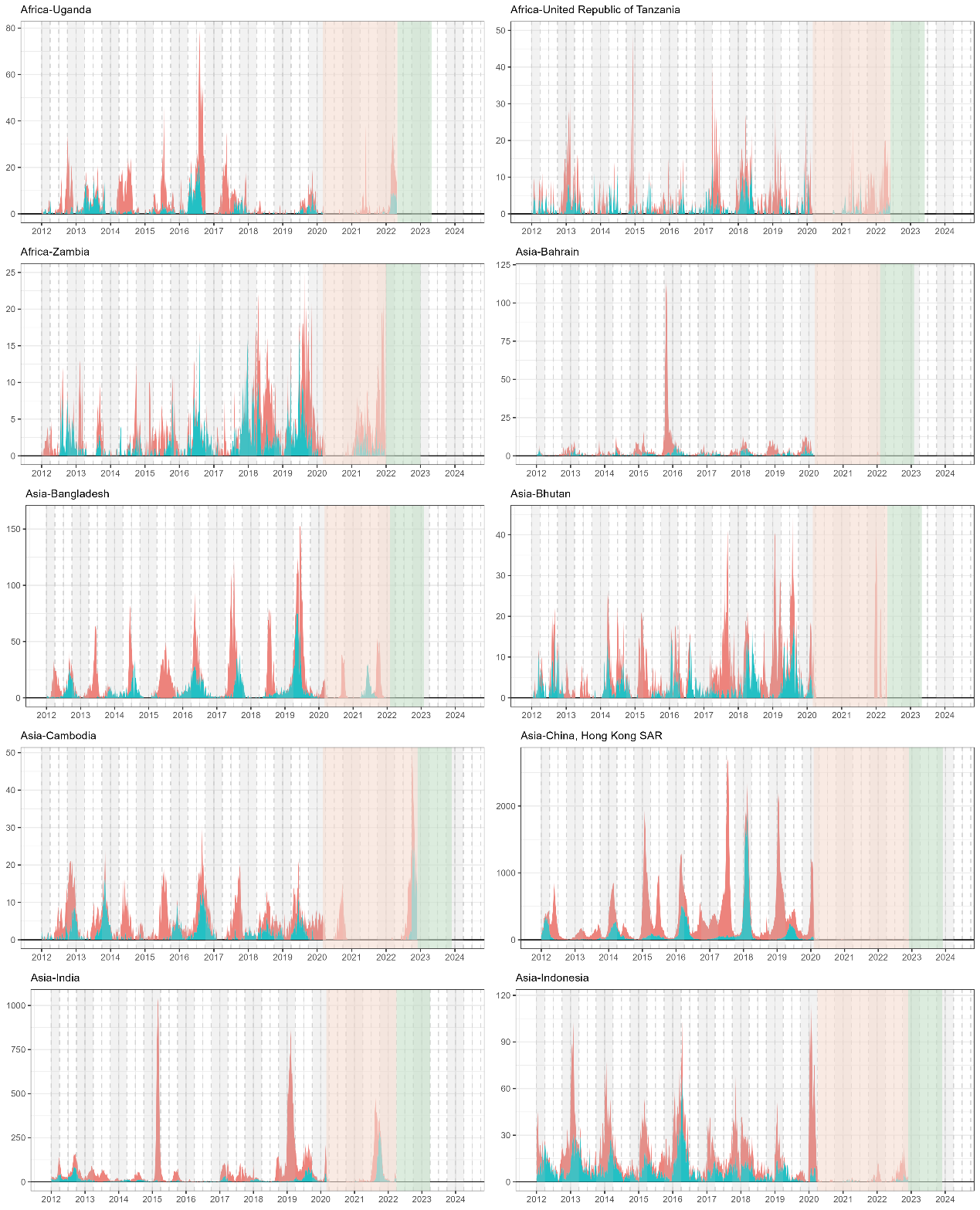


Figure S16. total influenza cases of restrictions of Uganda, United Republic of Tanzania, Zambia, Bahrain, Bangladesh, Bhutan, Cambodia, China, Hong Kong SAR ,India, Indonesia.


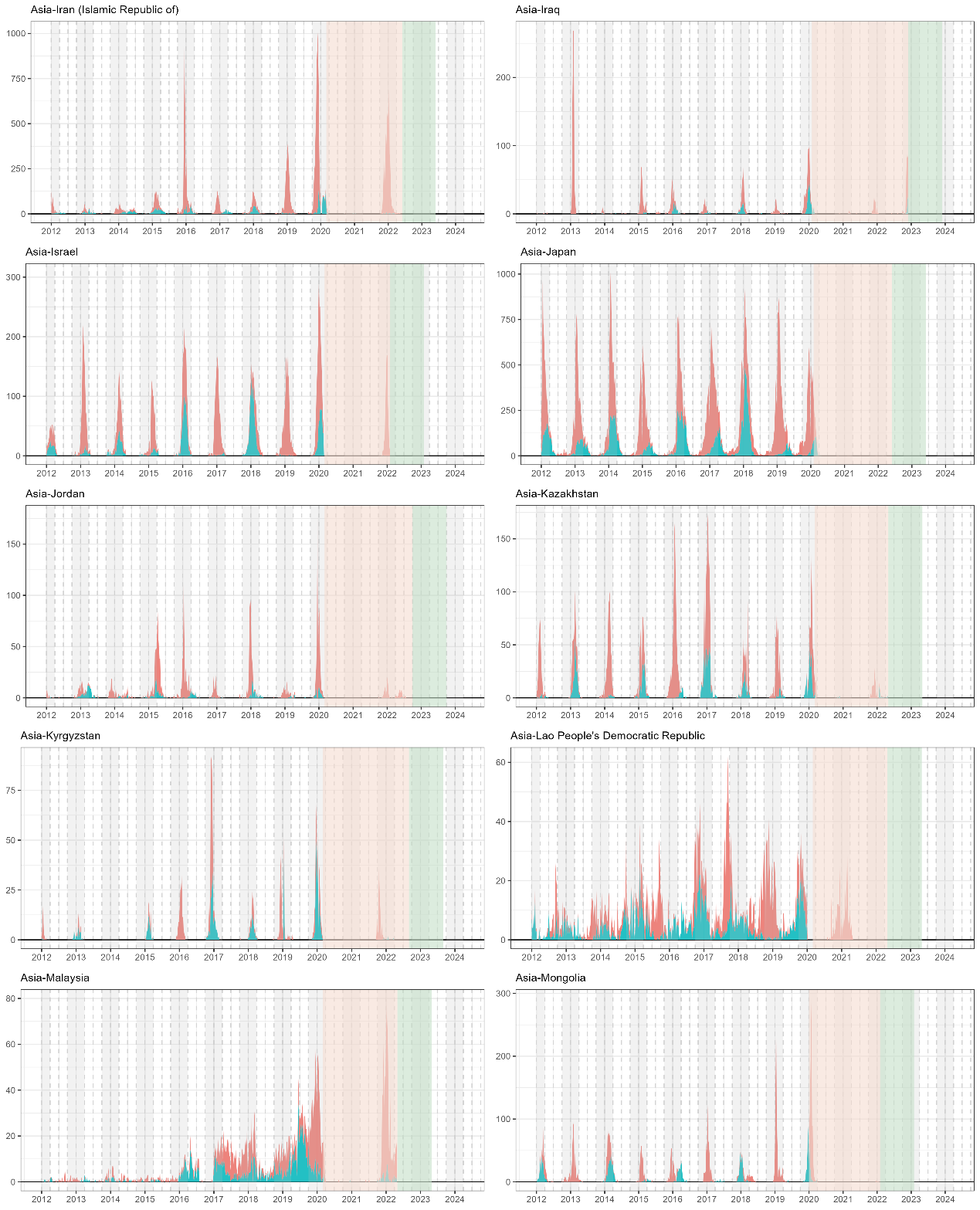


Figure S17. total influenza cases of Iran (Islamic Republic of), Iraq, Israel, Japan, Jordan, Kazakhstan, Kyrgyzstan, Lao People's Democratic Republic, Malaysia, and Mongolia.


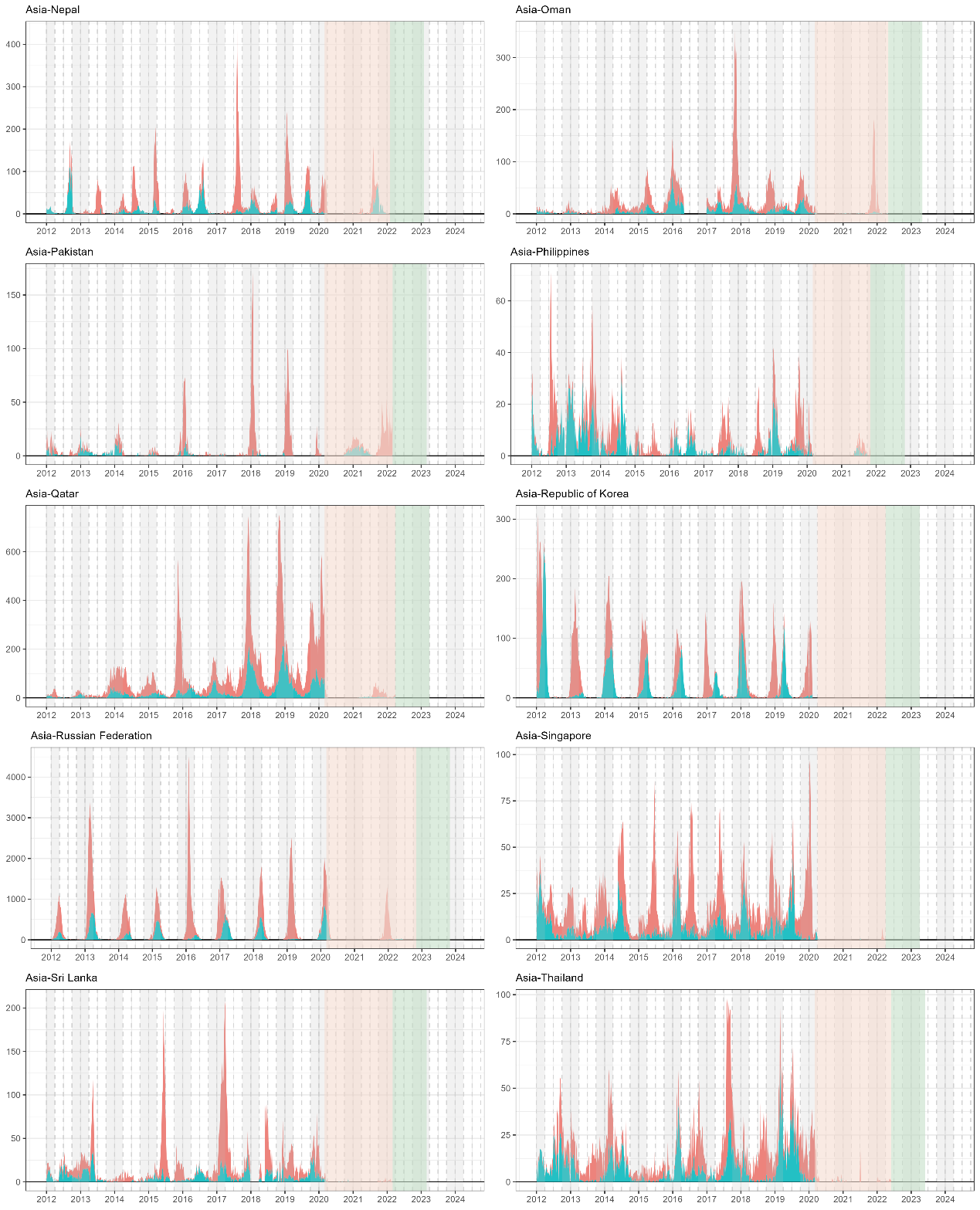


Figure S18. total influenza cases of Nepal, Oman, Pakistan, Philippines, Qatar, Republic of Korea, Russian Federation, Singapore, Sri Lanka, and Thailand.


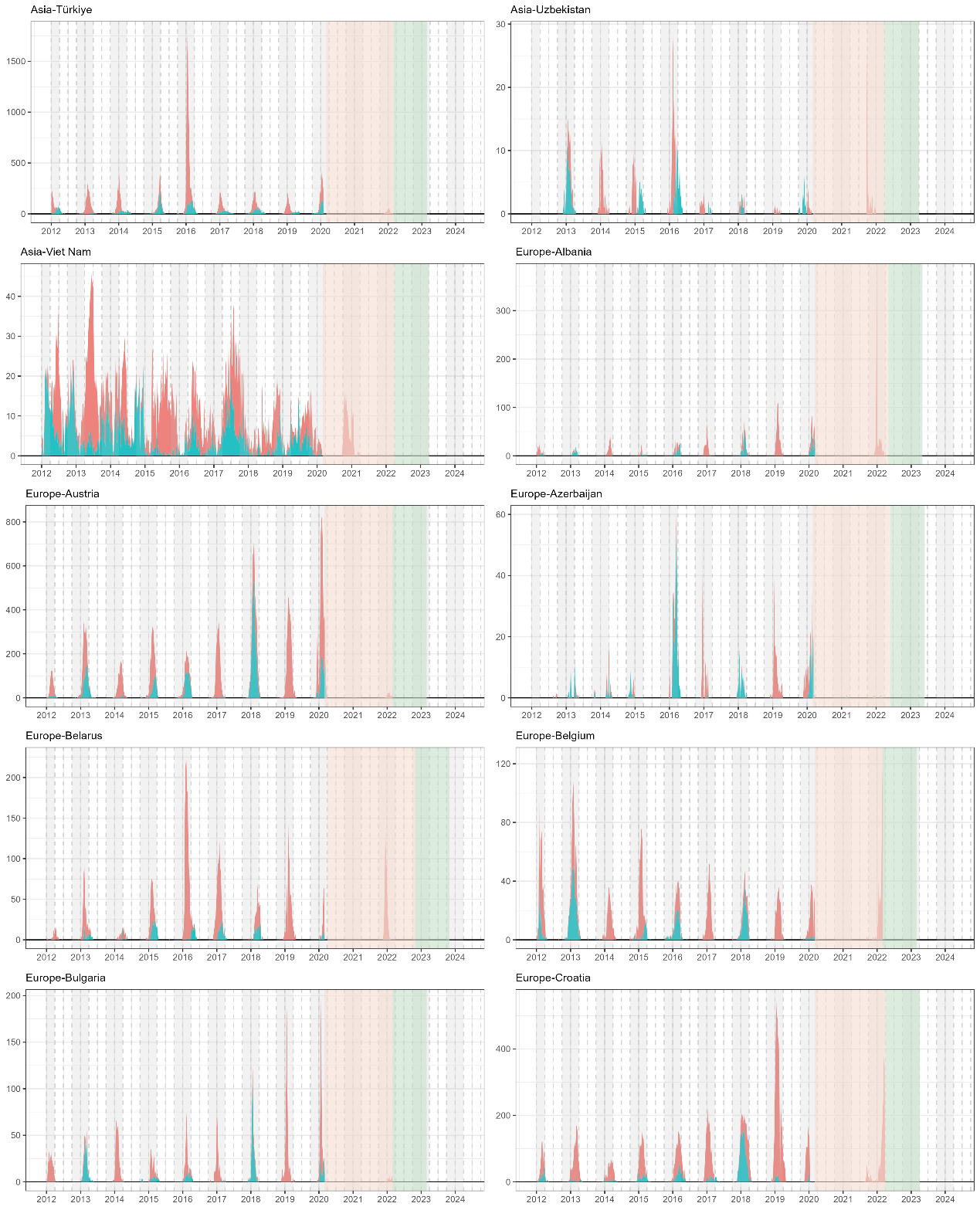


Figure S19. total influenza cases of Türkiye, Uzbekistan, Viet Nam, Albania, Austria, Azerbaijan, Belarus, Belgium, Bulgaria, and Croatia.


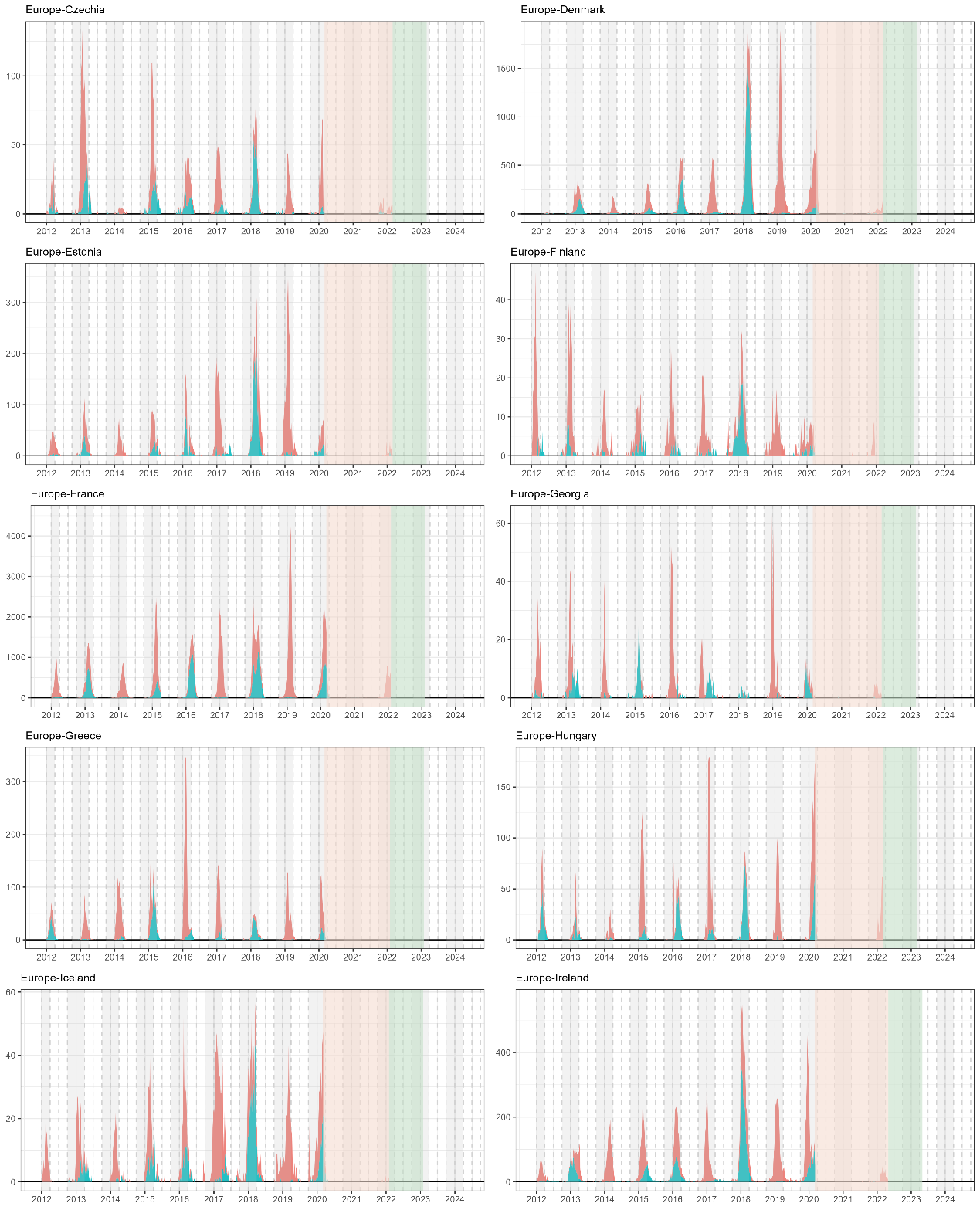


Figure S20. total influenza cases of Czechia, Denmark, Estonia, Finland, France, Georgia, Greece, Hungary, Iceland, and Ireland.


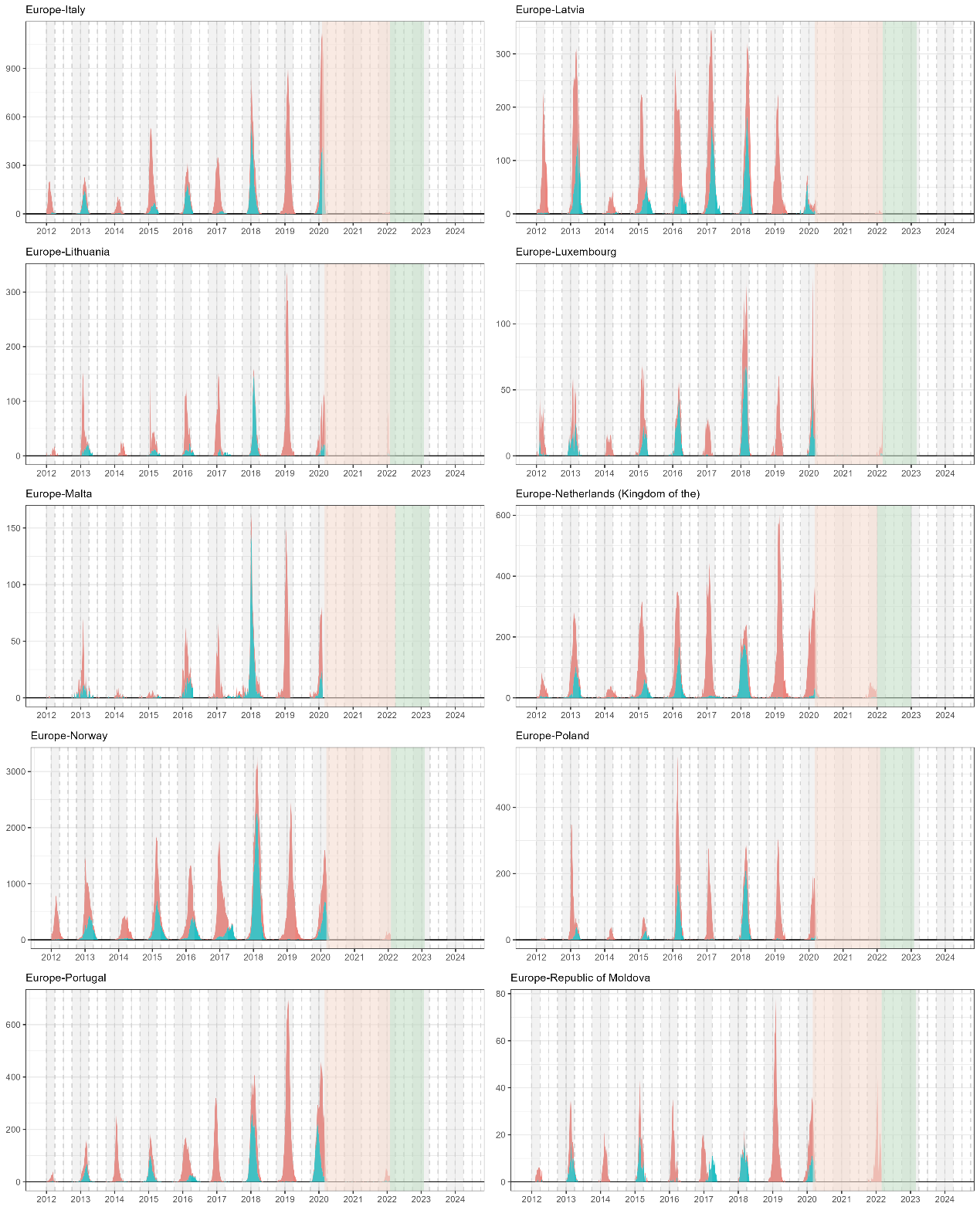


Figure S21. total influenza cases of Italy, Latvia, Lithuania, Luxembourg, Malta, Netherlands (Kingdom of the), Norway, Poland, Portugal, and Republic of Moldova.


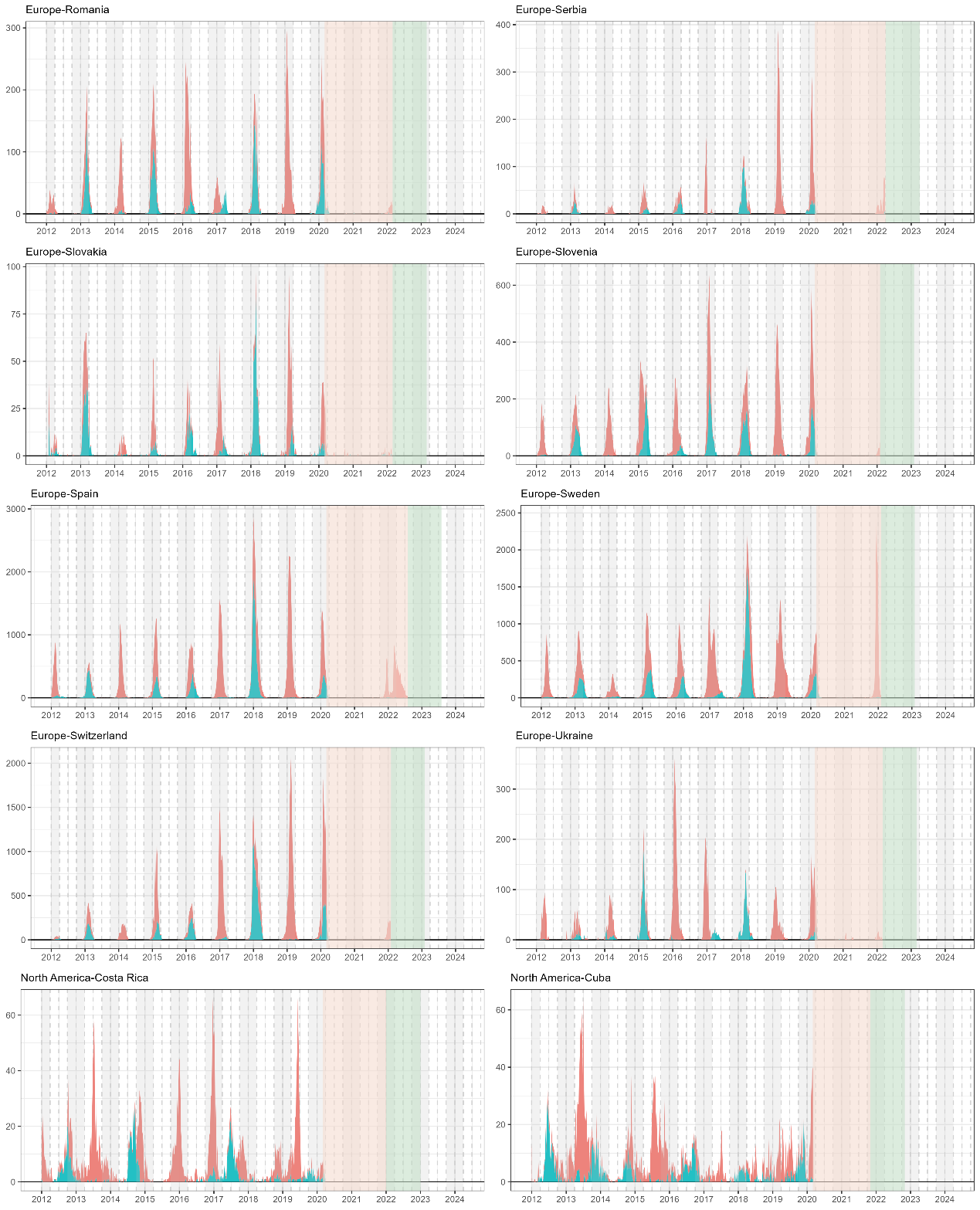


Figure S22. total influenza cases of Romania, Serbia, Slovakia, Slovenia, Spain, Sweden, Switzerland, Ukraine, Costa Rica, and Cuba.


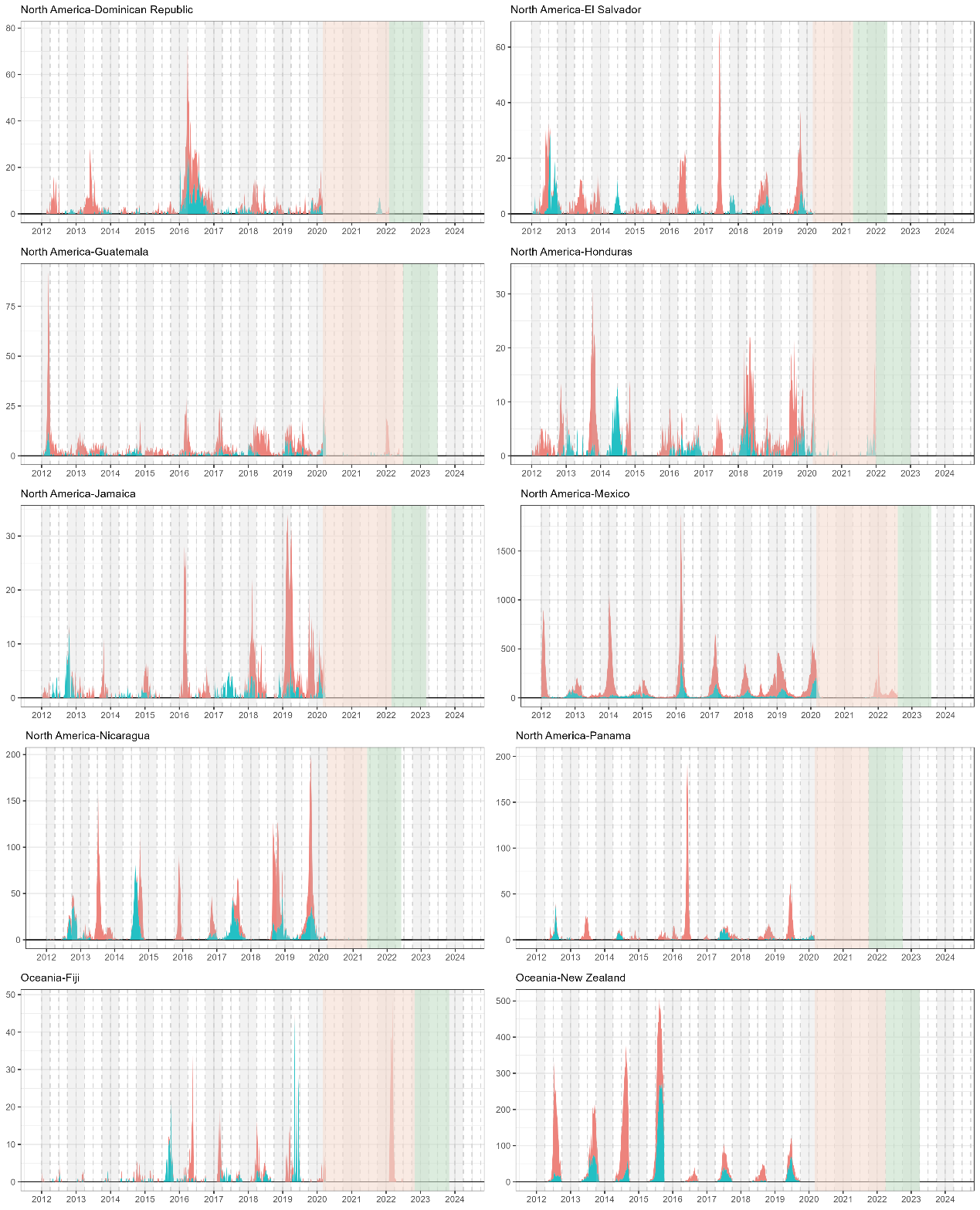


Figure S23. total influenza cases of Dominican Republic, El Salvador, Guatemala, Honduras, Jamaica, Mexico, Nicaragua, Panama, Fiji, and New Zealand.


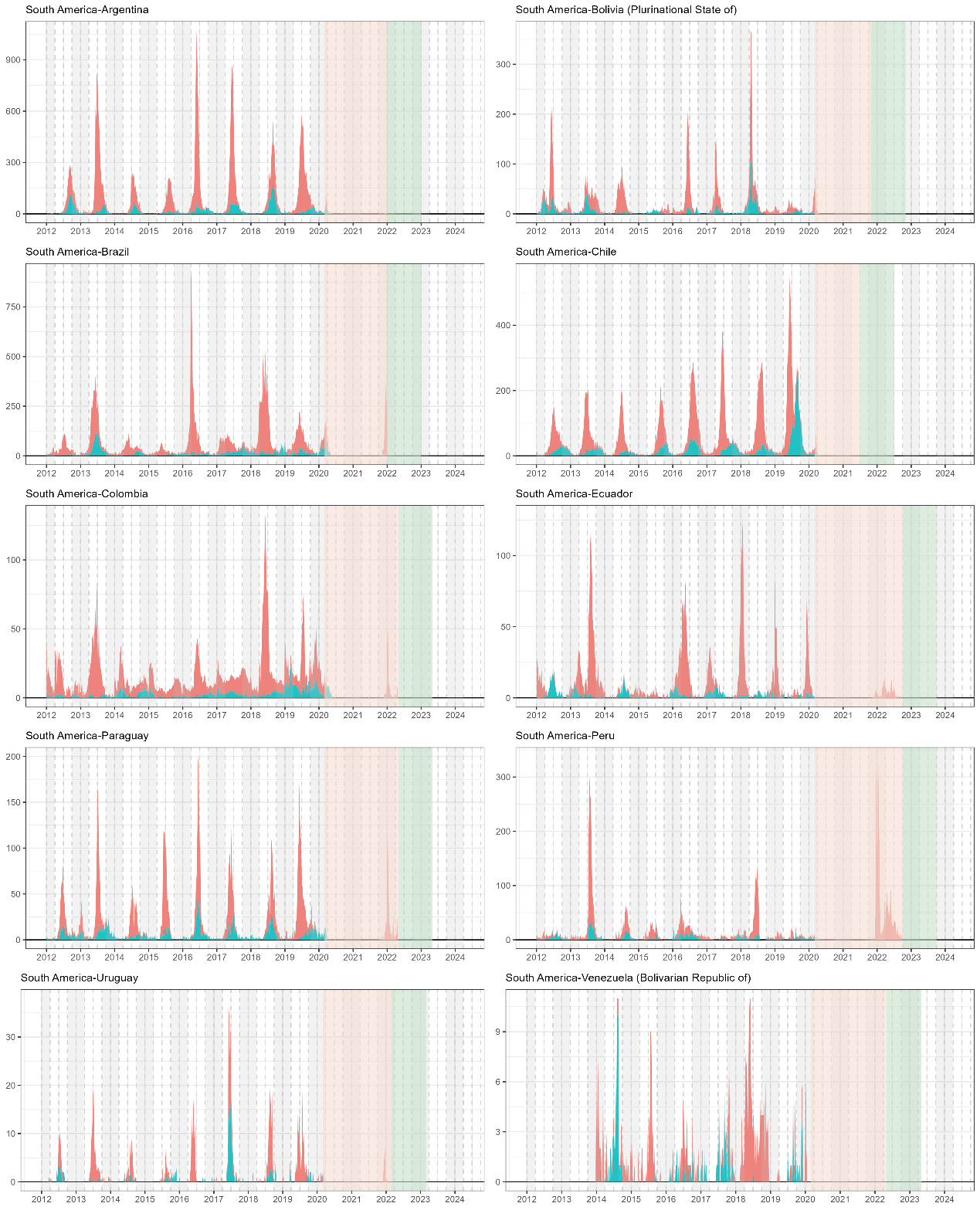


Figure S24. total influenza cases of Argentina, Bolivia (Plurinational State of), Brazil, Chile, Colombia, Ecuador, Paraguay, Peru, Uruguay, and Venezuela (Bolivarian Republic of).

Table S3. The reduction in total influenza activity levels during COVID-19 restriction period across continents and top 5 countries of each continent.

| Q14 | | |  | | Q23 | | | |
| --- | --- | --- | --- | --- | --- | --- | --- | --- |
| Continents or Top 5 countries | Reduction | 95%CI |  | Continents or Top 5 countries | | Reduction | 95%CI |  |
| **Overall** | **-46.30** | **-70.78 to -15.79** |  | **Overall** | | **-16.63** | **-53.83 to 45.27** |  |
| **Africa** | -25.95 | -56.61 to 11.01 |  | **Africa** | | -25.43 | -53.21 to 12.54 |  |
| Rwanda | -100.00 | -100.00 to -100.00 |  | Algeria | | -100.00 | -100.00 to -100.00 |  |
| Central African Republic | -100.00 | -100.00 to -100.00 |  | Morocco | | -100.00 | -100.00 to -100.00 |  |
| Morocco | -98.40 | -100.00 to -93.40 |  | Rwanda | | -100.00 | -100.00 to -100.00 |  |
| Tunisia | -84.60 | -96.71 to -67.24 |  | Tunisia | | -100.00 | -100.00 to -100.00 |  |
| Nigeria | -79.67 | -90.78 to -66.71 |  | Central African Republic | | -100.00 | -100.00 to -100.00 |  |
| **Asia** | -52.13 | -72.36 to -26.79 |  | **Asia** | | -4.89 | -47.99 to 68.30 |  |
| Republic of Korea | -100.00 | -100.00 to -100.00 |  | Israel | | -100.00 | -100.00 to -100.00 |  |
| Singapore | -98.67 | -99.80 to -96.90 |  | Republic of Korea | | -100.00 | -100.00 to -100.00 |  |
| Bahrain | -98.30 | -100.00 to -95.22 |  | Mongolia | | -100.00 | -100.00 to -100.00 |  |
| Philippines | -97.70 | -100.00 to -92.95 |  | Türkiye | | -100.00 | -100.00 to -100.00 |  |
| China, Hong Kong SAR | -97.60 | -99.15 to -94.96 |  | Bahrain | | -100.00 | -100.00 to -100.00 |  |
| **Europe** | -71.68 | -89.13 to -48.41 |  | **Europe** | | -53.75 | -77.78 to -5.25 |  |
| Malta | -100.00 | -100.00 to -100.00 |  | Austria | | -100.00 | -100.00 to -100.00 |  |
| Greece | -97.57 | -99.95 to -92.48 |  | Estonia | | -100.00 | -100.00 to -100.00 |  |
| Latvia | -96.97 | -99.06 to -94.41 |  | Greece | | -100.00 | -100.00 to -100.00 |  |
| Bulgaria | -94.25 | -98.06 to -89.07 |  | Republic of Moldova | | -100.00 | -100.00 to -100.00 |  |
| Slovenia | -91.77 | -97.76 to -82.46 |  | Hungary | | -100.00 | -100.00 to -100.00 |  |
| **North America** | -65.30 | -80.08 to -45.72 |  | **North America** | | 8.30 | -41.19 to 86.52 |  |
| El Salvador | -98.72 | -100.00 to -95.92 |  | Jamaica | | -100.00 | -100.00 to -100.00 |  |
| Nicaragua | -98.52 | -100.00 to -96.33 |  | El Salvador | | -100.00 | -100.00 to -100.00 |  |
| Costa Rica | -95.46 | -100.00 to -89.44 |  | Costa Rica | | -100.00 | -100.00 to -100.00 |  |
| Panama | -93.88 | -100.00 to -79.11 |  | Cuba | | -100.00 | -100.00 to -100.00 |  |
| Jamaica | -90.59 | -99.17 to -77.20 |  | Dominican Republic | | -99.61 | -100.00 to -98.61 |  |
| **Oceania** | -55.40 | -77.62 to -25.63 |  | **Oceania** | | -93.47 | -99.62 to -86.27 |  |
| New Zealand | -100.00 | -100.00 to -100.00 |  | New Zealand | | -100.00 | -100.00 to -100.00 |  |
| Australia | -55.76 | -76.23 to -29.69 |  | Fiji | | -98.17 | -100.00 to -95.49 |  |
| Fiji | 200.26 | 6.86 to 498.04 |  | Australia | | -92.01 | -99.53 to -83.23 |  |
| **South America** | 23.60 | -52.15 to 116.30 |  | **South America** | | -93.00 | -96.83 to -87.48 |  |
| Venezuela (Bolivarian Republic of) | -100.00 | -100.00 to -100.00 |  | Uruguay | | -100.00 | -100.00 to -100.00 |  |
| Ecuador | -88.51 | -94.49 to -81.35 |  | Venezuela (Bolivarian Republic of) | | -100.00 | -100.00 to -100.00 |  |
| Colombia | -72.99 | -89.40 to -50.54 |  | Bolivia (Plurinational State of) | | -99.83 | -100.00 to -99.55 |  |
| Chile | -71.97 | -95.58 to -39.82 |  | Chile | | -99.72 | -99.93 to -99.38 |  |
| Argentina | -54.61 | -89.22 to -6.80 |  | Argentina | | -99.17 | -99.64 to -98.39 |  |


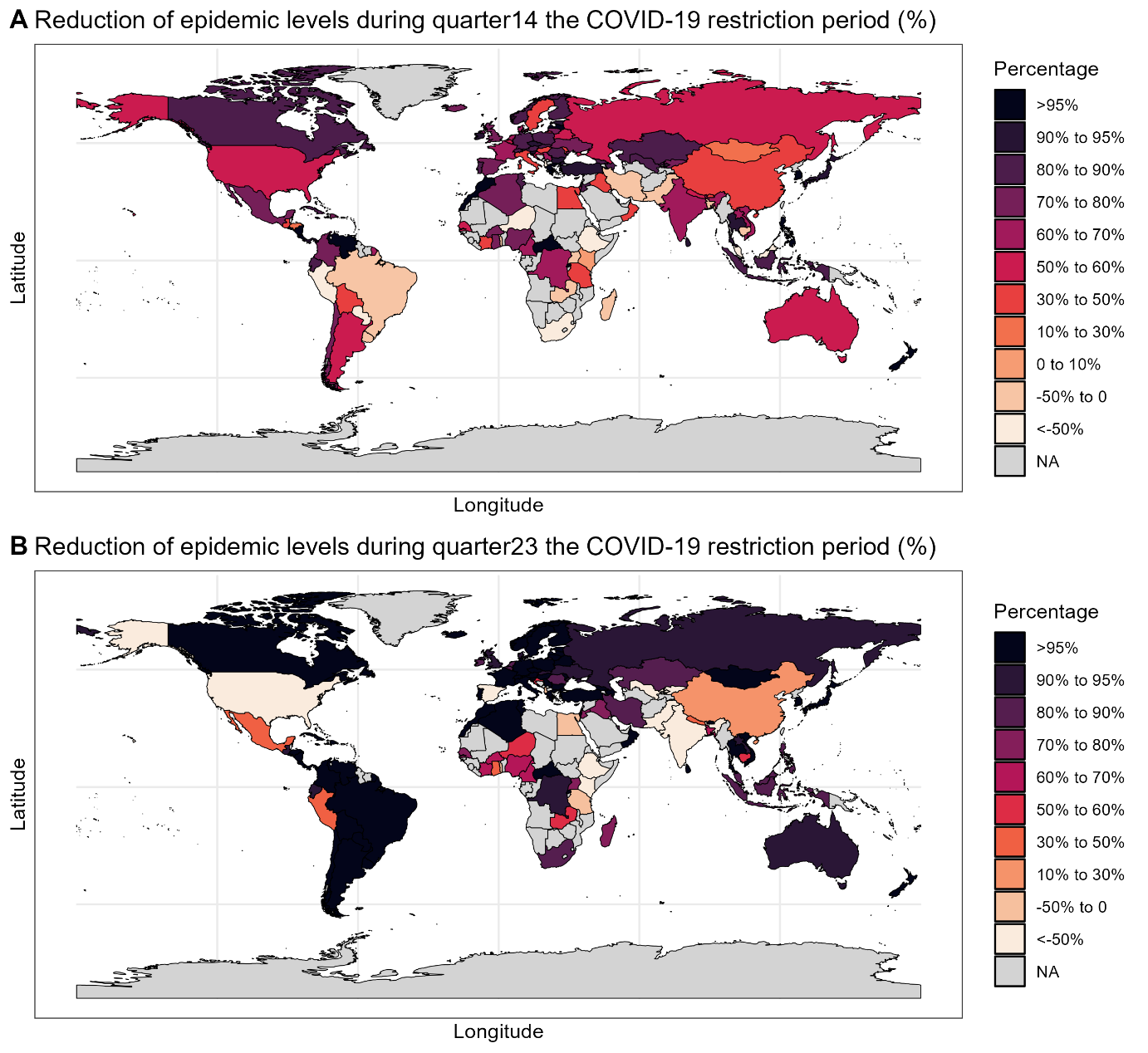


Figure S25. Reduction of epidemic levels during the COVID-19 restriction period.

Table S4. The reduction in influenza A activity levels during COVID-19 restriction period across continents and top 5 countries of each continent.

| Q14 | | |  | Q23 | | |
| --- | --- | --- | --- | --- | --- | --- |
| Continents or Top 5 countries | Reduction | 95%CI |  | Continents or Top 5 countries | Reduction | 95%CI |
| **Overall** | **-46.76** | **-74.56 to -9.62** |  | **Overall** | **-8.80** | **-52.41 to 57.25** |
| **Africa** | -3.77 | -47.73 to 53.96 |  | **Africa** | -26.76 | -56.38 to 14.94 |
| Rwanda | -100.00 | -100.00 to -100.00 |  | Algeria | -100.00 | -100.00 to -100.00 |
| Central African Republic | -100.00 | -100.00 to -100.00 |  | Rwanda | -100.00 | -100.00 to -100.00 |
| Morocco | -98.09 | -100.00 to -91.94 |  | Morocco | -100.00 | -100.00 to -100.00 |
| Tunisia | -82.29 | -95.80 to -62.56 |  | Tunisia | -100.00 | -100.00 to -100.00 |
| Senegal | -80.46 | -95.95 to -42.52 |  | Central African Republic | -100.00 | -100.00 to -100.00 |
| **Asia** | -63.34 | -82.27 to -37.88 |  | **Asia** | -8.88 | -53.65 to 55.22 |
| Republic of Korea | -100.00 | -100.00 to -100.00 |  | Israel | -100.00 | -100.00 to -100.00 |
| Bahrain | -98.32 | -100.00 to -95.20 |  | Republic of Korea | -100.00 | -100.00 to -100.00 |
| Singapore | -97.97 | -99.70 to -95.26 |  | Kazakhstan | -100.00 | -100.00 to -100.00 |
| China, Hong Kong SAR | -97.35 | -99.34 to -93.87 |  | Mongolia | -100.00 | -100.00 to -100.00 |
| Japan | -95.90 | -99.21 to -91.77 |  | Türkiye | -100.00 | -100.00 to -100.00 |
| **Europe** | -68.97 | -87.81 to -43.50 |  | **Europe** | -19.42 | -61.69 to 65.47 |
| Malta | -100.00 | -100.00 to -100.00 |  | Iceland | -100.00 | -100.00 to -100.00 |
| Greece | -98.71 | -100.00 to -95.82 |  | Austria | -100.00 | -100.00 to -100.00 |
| Latvia | -96.45 | -98.78 to -93.64 |  | Luxembourg | -100.00 | -100.00 to -100.00 |
| Azerbaijan | -96.41 | -100.00 to -90.64 |  | Estonia | -100.00 | -100.00 to -100.00 |
| Bulgaria | -94.68 | -98.26 to -89.56 |  | Malta | -100.00 | -100.00 to -100.00 |
| **North America** | -61.24 | -77.77 to -39.28 |  | **North America** | 105.90 | 10.76 to 263.40 |
| El Salvador | -100.00 | -100.00 to -100.00 |  | Jamaica | -100.00 | -100.00 to -100.00 |
| Nicaragua | -100.00 | -100.00 to -100.00 |  | El Salvador | -100.00 | -100.00 to -100.00 |
| Costa Rica | -96.55 | -100.00 to -90.16 |  | Costa Rica | -100.00 | -100.00 to -100.00 |
| Panama | -96.27 | -100.00 to -87.12 |  | Panama | -100.00 | -100.00 to -100.00 |
| Dominican Republic | -93.90 | -99.41 to -86.31 |  | Honduras | -100.00 | -100.00 to -100.00 |
| **Oceania** | -60.69 | -81.42 to -33.09 |  | **Oceania** | -94.63 | -99.73 to -88.58 |
| New Zealand | -100.00 | -100.00 to -100.00 |  | New Zealand | -100.00 | -100.00 to -100.00 |
| Australia | -65.89 | -81.83 to -46.68 |  | Fiji | -95.96 | -100.00 to -89.59 |
| Fiji | 301.56 | 32.40 to 715.93 |  | Australia | -93.53 | -99.66 to -86.30 |
| **South America** | 68.10 | -39.25 to 211.70 |  | **South America** | -92.91 | -96.70 to -86.91 |
| Venezuela (Bolivarian Republic of) | -100.00 | -100.00 to -100.00 |  | Uruguay | -100.00 | -100.00 to -100.00 |
| Ecuador | -87.30 | -94.20 to -79.01 |  | Venezuela (Bolivarian Republic of) | -100.00 | -100.00 to -100.00 |
| Colombia | -71.49 | -91.66 to -43.62 |  | Chile | -99.82 | -99.97 to -99.59 |
| Argentina | -40.48 | -85.39 to 24.04 |  | Bolivia (Plurinational State of) | -99.78 | -100.00 to -99.41 |
| Chile | -39.94 | -90.72 to 33.20 |  | Argentina | -99.31 | -99.76 to -98.51 |


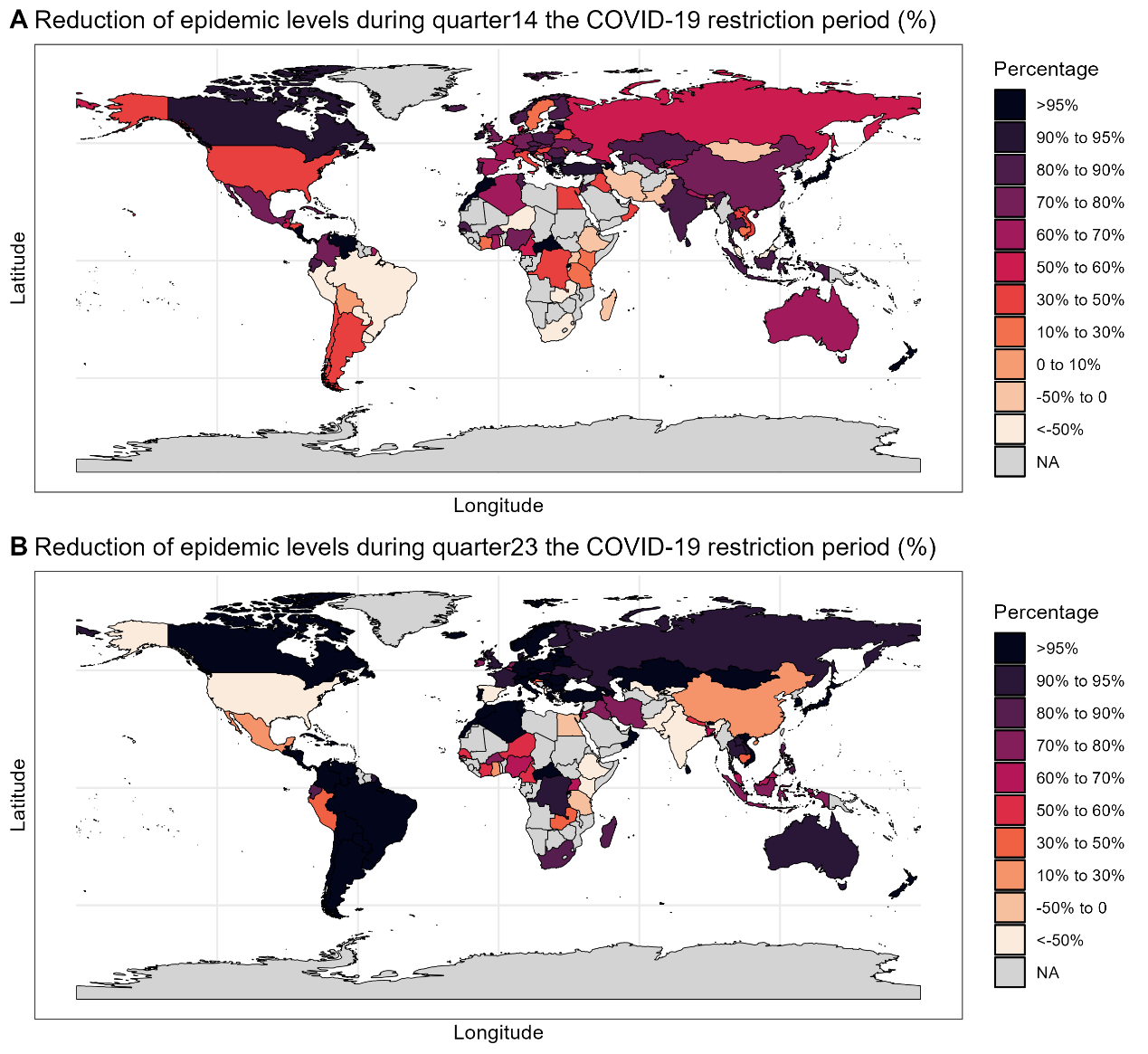


Figure S26. Reduction of influenza A epidemic levels during the COVID-19 restriction period.

Table S5. The reduction in influenza B activity levels during COVID-19 restriction period across continents and top 5 countries of each continent.

| Q14 | | |  | Q23 | | |
| --- | --- | --- | --- | --- | --- | --- |
| Continents or Top 5 countries | Reduction | 95%CI |  | Continents or Top 5 countries | Reduction | 95%CI |
| **Overall** | **0.53** | **-52.57 to 69.15** |  | **Overall** | **-28.06** | **-70.79 to 38.83** |
| **Africa** | -43.76 | -72.16 to -4.15 |  | **Africa** | -21.49 | -61.71 to 47.87 |
| Central African Republic | -100.00 | -100.00 to -100.00 |  | Algeria | -100.00 | -100.00 to -100.00 |
| Rwanda | -100.00 | -100.00 to -100.00 |  | Morocco | -100.00 | -100.00 to -100.00 |
| Morocco | -99.20 | -100.00 to -96.68 |  | Tunisia | -100.00 | -100.00 to -100.00 |
| Algeria | -97.72 | -100.00 to -93.82 |  | Central African Republic | -100.00 | -100.00 to -100.00 |
| Democratic Republic of the Congo | -95.50 | -100.00 to -88.32 |  | Rwanda | -100.00 | -100.00 to -100.00 |
| **Asia** | 37.94 | -32.99 to 128.49 |  | **Asia** | -5.86 | -63.32 to 82.20 |
| Republic of Korea | -100.00 | -100.00 to -100.00 |  | Republic of Korea | -100.00 | -100.00 to -100.00 |
| Jordan | -100.00 | -100.00 to -100.00 |  | Türkiye | -100.00 | -100.00 to -100.00 |
| Singapore | -100.00 | -100.00 to -100.00 |  | Israel | -100.00 | -100.00 to -100.00 |
| Uzbekistan | -100.00 | -100.00 to -100.00 |  | Iraq | -100.00 | -100.00 to -100.00 |
| Viet Nam | -100.00 | -100.00 to -100.00 |  | Bahrain | -100.00 | -100.00 to -100.00 |
| **Europe** | -80.07 | -95.70 to -55.72 |  | **Europe** | -96.82 | -99.28 to -90.02 |
| Croatia | -100.00 | -100.00 to -100.00 |  | Latvia | -100.00 | -100.00 to -100.00 |
| Malta | -100.00 | -100.00 to -100.00 |  | Republic of Moldova | -100.00 | -100.00 to -100.00 |
| Latvia | -98.53 | -100.00 to -96.11 |  | Czechia | -100.00 | -100.00 to -100.00 |
| Belarus | -97.74 | -100.00 to -94.22 |  | Belarus | -100.00 | -100.00 to -100.00 |
| Georgia | -97.14 | -100.00 to -91.72 |  | Hungary | -100.00 | -100.00 to -100.00 |
| **North America** | -76.37 | -93.68 to -53.10 |  | **North America** | -93.50 | -95.88 to -89.17 |
| Cuba | -98.77 | -100.00 to -95.16 |  | El Salvador | -100.00 | -100.00 to -100.00 |
| El Salvador | -96.34 | -100.00 to -88.07 |  | Costa Rica | -100.00 | -100.00 to -100.00 |
| Nicaragua | -95.12 | -100.00 to -87.87 |  | Cuba | -100.00 | -100.00 to -100.00 |
| Costa Rica | -87.76 | -100.00 to -66.29 |  | Jamaica | -100.00 | -100.00 to -100.00 |
| United States of America | -85.94 | -96.18 to -70.84 |  | Dominican Republic | -98.41 | -100.00 to -93.19 |
| **Oceania** | -27.35 | -70.06 to 32.72 |  | **Oceania** | -89.03 | -99.10 to -76.61 |
| New Zealand | -100.00 | -100.00 to -100.00 |  | New Zealand | -100.00 | -100.00 to -100.00 |
| Fiji | -94.82 | -100.00 to -78.74 |  | Fiji | -100.00 | -100.00 to -100.00 |
| Australia | -10.62 | -63.09 to 62.85 |  | Australia | -86.48 | -98.89 to -71.17 |
| **South America** | -63.12 | -92.34 to -28.55 |  | **South America** | -91.05 | -97.49 to -82.30 |
| Venezuela (Bolivarian Republic of) | -100.00 | -100.00 to -100.00 |  | Venezuela (Bolivarian Republic of) | -100.00 | -100.00 to -100.00 |
| Ecuador | -97.57 | -100.00 to -91.78 |  | Uruguay | -100.00 | -100.00 to -100.00 |
| Bolivia (Plurinational State of) | -94.62 | -100.00 to -82.71 |  | Bolivia (Plurinational State of) | -100.00 | -100.00 to -100.00 |
| Peru | -93.78 | -100.00 to -82.81 |  | Ecuador | -100.00 | -100.00 to -100.00 |
| Chile | -93.41 | -99.23 to -85.13 |  | Chile | -99.35 | -99.90 to -98.45 |


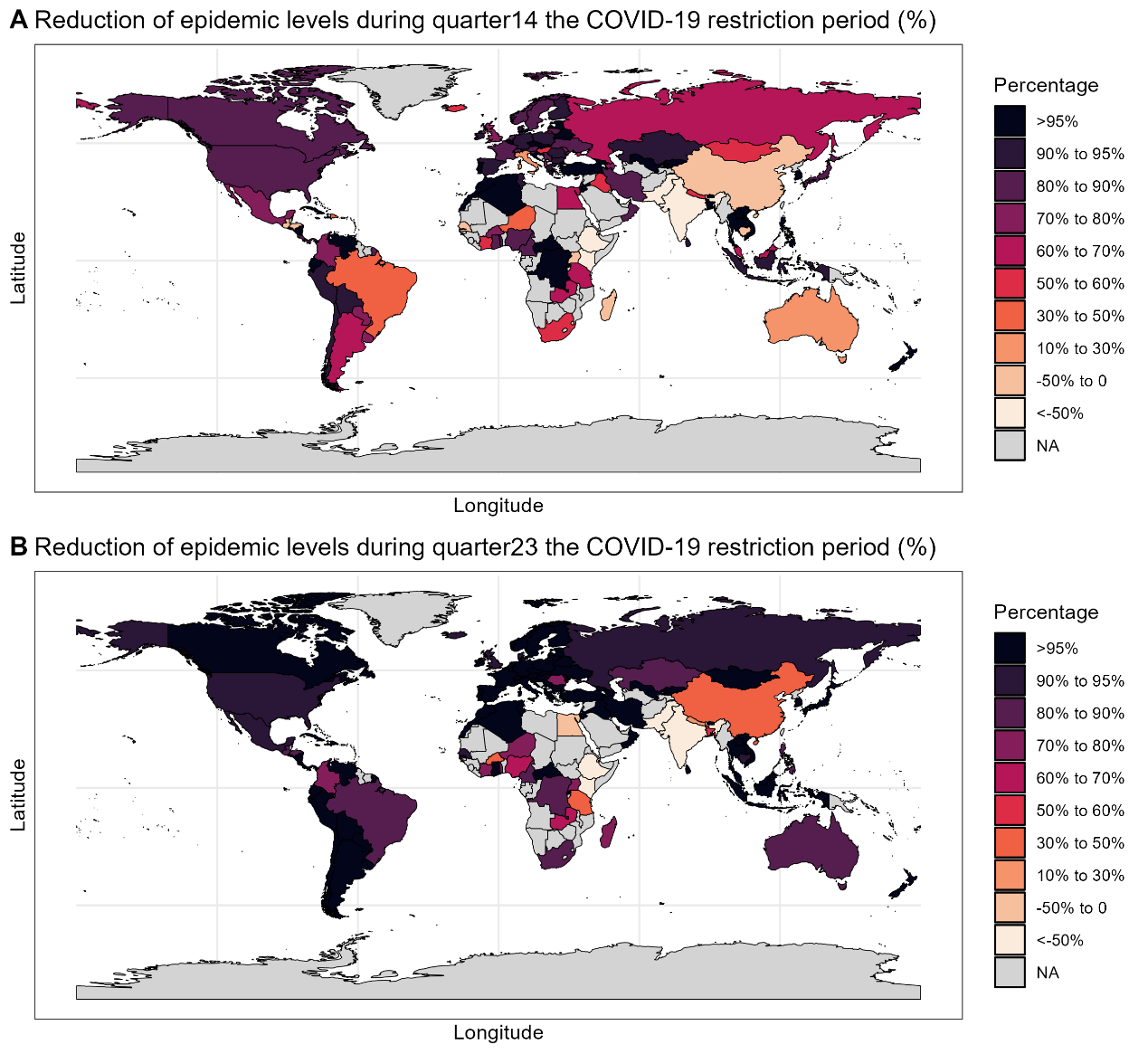


Figure S27. Reduction of influenza B epidemic levels during the COVID-19 restriction period.

Table S6. The increase in influenza A activity levels during COVID-19 restriction period across continents and top 5 countries of each continent.

| Q14 |  |  |  | Q23 |  |  |
| --- | --- | --- | --- | --- | --- | --- |
| Continents or Top 5 countries | Immunity debt Percentage | 95%CI |  | Continents or Top 5 countries | Immunity debt Percentage | 95%CI |
| **Overall** | **168.71** | **43.84 to 336.52** |  | **Overall** | **245.91** | **55.88 to 577.39** |
| **Africa** | 64.34 | -4.44 to 157.02 |  | **Africa** | 57.70 | -16.03 to 179.37 |
| Ethiopia | 379.55 | 207.54 to 593.68 |  | Ghana | 480.74 | 142.65 to 942.75 |
| South Africa | 257.14 | 106.34 to 483.23 |  | Egypt | 452.50 | 242.73 to 750.11 |
| United Republic of Tanzania | 210.68 | 78.65 to 387.59 |  | Mauritius | 248.63 | 75.79 to 529.21 |
| Madagascar | 167.34 | 33.08 to 321.11 |  | Togo | 187.04 | 75.93 to 356.98 |
| Kenya | 125.67 | 40.52 to 226.68 |  | United Republic of Tanzania | 181.72 | 67.28 to 351.31 |
| **Asia** | 120.09 | 24.33 to 239.21 |  | **Asia** | 191.04 | 46.36 to 395.37 |
| Bahrain | 592.01 | 211.22 to 1154.33 |  | Bahrain | 11053.49 | 7132.15 to 16178.93 |
| Iran (Islamic Republic of) | 526.93 | 186.08 to 1077.40 |  | Malaysia | 3780.70 | 2957.09 to 6010.34 |
| Pakistan | 374.69 | 174.73 to 641.62 |  | Mongolia | 973.42 | 240.20 to 2239.51 |
| China | 361.38 | 157.15 to 597.32 |  | Pakistan | 668.93 | 322.33 to 1197.51 |
| Malaysia | 360.63 | 241.94 to 519.39 |  | Iran (Islamic Republic of) | 638.26 | 256.10 to 1200.12 |
| **Europe** | 196.18 | 58.93 to 377.58 |  | **Europe** | 1359.56 | 380.61 to 3454.03 |
| Ukraine | 1143.61 | 566.60 to 1862.65 |  | Portugal | 11978.48 | 5075.98 to 28484.40 |
| Czechia | 931.84 | 400.87 to 1631.94 |  | Netherlands (Kingdom of the) | 4558.37 | 1454.06 to 9599.81 |
| Portugal | 490.07 | 275.66 to 724.50 |  | Czechia | 3952.63 | 1231.67 to 8540.96 |
| Denmark | 386.14 | 174.03 to 681.78 |  | Switzerland | 3373.12 | 440.12 to 10468.69 |
| Switzerland | 338.10 | 158.10 to 607.24 |  | Luxembourg | 2640.00 | 654.64 to 7233.48 |
| **North America** | 171.30 | 41.38 to 333.28 |  | **North America** | 82.79 | 14.57 to 190.09 |
| United States of America | 241.62 | 79.52 to 437.64 |  | Canada | 566.79 | 250.88 to 1093.09 |
| Panama | 230.72 | -19.45 to 626.59 |  | Mexico | 123.62 | 34.03 to 238.98 |
| Honduras | 180.04 | -68.38 to 669.30 |  | Guatemala | 88.78 | 36.36 to 155.52 |
| Canada | 109.49 | -1.36 to 238.24 |  | Panama | 84.86 | -1.85 to 232.69 |
| Dominican Republic | 105.94 | 25.11 to 212.74 |  | Costa Rica | 63.74 | -0.34 to 161.15 |
| **Oceania** | 81.62 | 21.00 to 324.51 |  | **Oceania** | 107.44 | 39.59 to 260.28 |
| Fiji | 621.39 | 128.02 to 1547.88 |  | Australia | 153.85 | 71.36 to 340.21 |
| New Zealand | 166.99 | -0.72 to 1365.90 |  | Fiji | -47.47 | -79.64 to 10.38 |
| Australia | 45.50 | 21.46 to 75.58 |  | New Zealand | -100.00 | -100.00 to -100.00 |
| **South America** | 907.56 | 354.68 to 1758.81 |  | **South America** | -15.88 | -54.37 to 47.00 |
| Argentina | 5108.46 | 2747.88 to 8472.62 |  | Venezuela (Bolivarian Republic of) | 202.68 | 18.70 to 557.27 |
| Venezuela (Bolivarian Republic of) | 1508.44 | 518.05 to 2899.69 |  | Argentina | 125.72 | 43.27 to 239.56 |
| Uruguay | 559.72 | 91.13 to 1463.37 |  | Uruguay | 91.53 | 17.22 to 191.36 |
| Brazil | 433.82 | 31.35 to 1148.03 |  | Peru | 39.98 | -47.79 to 179.32 |
| Paraguay | 333.09 | 129.66 to 625.88 |  | Chile | 24.74 | -37.54 to 99.13 |


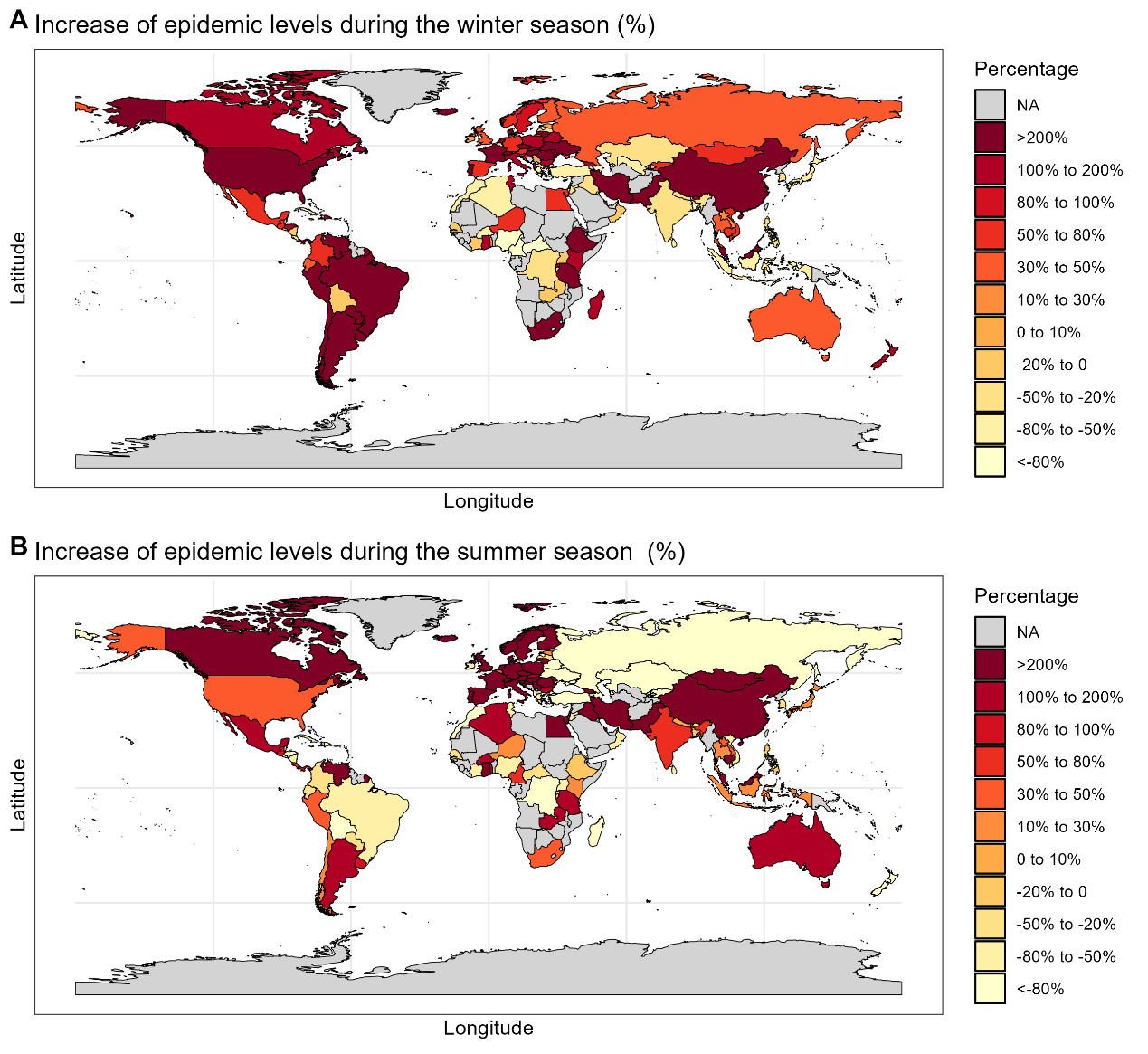


Figure S28. Increases of influenza A epidemic levels during the relaxation period.

Table S7. The increase in influenza B activity levels during COVID-19 restriction period across continents and top 5 countries of each continent.

| Q14 | | |  | Q23 | | |
| --- | --- | --- | --- | --- | --- | --- |
| Continents or Top 5 countries | Immunity debt Percentage | 95%CI |  | Continents or Top 5 countries | Immunity debt Percentage | 95%CI |
| **Overall** | **85.58** | **3.93 to 196.05** |  | **Overall** | **34.51** | **-35.90 to 164.62** |
| **Africa** | 31.83 | -35.45 to 131.12 |  | **Africa** | 2.08 | -42.59 to 81.00 |
| Kenya | 278.79 | 103.14 to 515.94 |  | United Republic of Tanzania | 196.99 | 85.19 to 369.11 |
| Tunisia | 167.65 | 5.15 to 419.60 |  | Egypt | 149.20 | 104.45 to 218.90 |
| Democratic Republic of the Congo | 163.16 | 29.28 to 353.77 |  | Ethiopia | 110.22 | 0.00 to 312.81 |
| Ghana | 161.35 | 19.96 to 344.75 |  | Niger | 77.78 | -39.93 to 438.40 |
| Ethiopia | 97.56 | 32.67 to 209.01 |  | Ghana | 52.53 | -40.98 to 184.10 |
| **Asia** | 95.59 | 17.47 to 197.37 |  | **Asia** | 44.98 | -30.93 to 175.92 |
| Malaysia | 5733.33 | 4222.16 to 7530.67 |  | Bahrain | 2806.12 | 1723.62 to 4344.24 |
| Bahrain | 900.66 | 488.46 to 1360.47 |  | Pakistan | 1972.73 | 923.78 to 3859.13 |
| Pakistan | 743.22 | 458.74 to 1150.20 |  | Iran (Islamic Republic of) | 774.07 | 295.35 to 1459.87 |
| Kazakhstan | 533.73 | 199.54 to 1006.25 |  | Malaysia | 143.79 | 56.85 to 347.90 |
| Iran (Islamic Republic of) | 518.18 | 355.63 to 760.81 |  | Lao People's Democratic Republic | 79.92 | 0.18 to 229.76 |
| **Europe** | 14.36 | -46.23 to 102.64 |  | **Europe** | 58.20 | -72.23 to 403.62 |
| Belarus | 1934.45 | 1018.12 to 3285.18 |  | Albania | 18414.29 | 0.00 to 70774.67 |
| Czechia | 368.24 | 89.83 to 751.49 |  | Luxembourg | 336.36 | 25.77 to 1268.88 |
| Denmark | 336.43 | 110.42 to 725.55 |  | Spain | 304.60 | 49.16 to 861.56 |
| Croatia | 199.72 | 45.35 to 423.87 |  | Netherlands (Kingdom of the) | 161.73 | 35.02 to 379.11 |
| Republic of Moldova | 116.28 | 5.07 to 277.13 |  | Serbia | 128.57 | -31.48 to 647.16 |
| **North America** | -64.06 | -77.64 to -47.37 |  | **North America** | 68.65 | 16.10 to 143.08 |
| Guatemala | 274.29 | 144.29 to 433.33 |  | Mexico | 441.11 | 275.22 to 674.33 |
| Honduras | 51.96 | -33.13 to 162.55 |  | Guatemala | 120.90 | 29.71 to 247.24 |
| Jamaica | 10.06 | -79.59 to 169.91 |  | United States of America | -28.31 | -50.17 to 3.14 |
| Mexico | -49.26 | -70.93 to -24.85 |  | Honduras | -85.78 | -100.00 to -57.30 |
| Canada | -75.10 | -87.26 to -59.31 |  | Canada | -95.05 | -96.65 to -92.55 |
| **Oceania** | 219.55 | 41.36 to 584.41 |  | **Oceania** | 8.82 | -15.49 to 78.92 |
| Fiji | 2786.47 | 785.33 to 7609.16 |  | Fiji | 199.16 | -72.18 to 911.69 |
| Australia | 173.38 | 38.85 to 349.11 |  | Australia | 23.45 | 3.19 to 84.40 |
| New Zealand | -11.86 | -80.89 to 503.98 |  | New Zealand | -100.00 | -100.00 to -100.00 |
| **South America** | 403.62 | 136.65 to 769.00 |  | **South America** | -68.29 | -90.98 to -35.62 |
| Argentina | 3383.82 | 1518.93 to 5742.10 |  | Argentina | 10.57 | -74.74 to 119.99 |
| Uruguay | 828.57 | 252.03 to 1967.33 |  | Colombia | -29.06 | -93.10 to 62.51 |
| Peru | 430.10 | 255.09 to 686.35 |  | Peru | -31.71 | -59.47 to 15.40 |
| Ecuador | 423.13 | 186.03 to 719.52 |  | Ecuador | -61.23 | -87.32 to -24.18 |
| Venezuela (Bolivarian Republic of) | 357.41 | 70.96 to 957.62 |  | Venezuela (Bolivarian Republic of) | -62.93 | -88.47 to -11.95 |


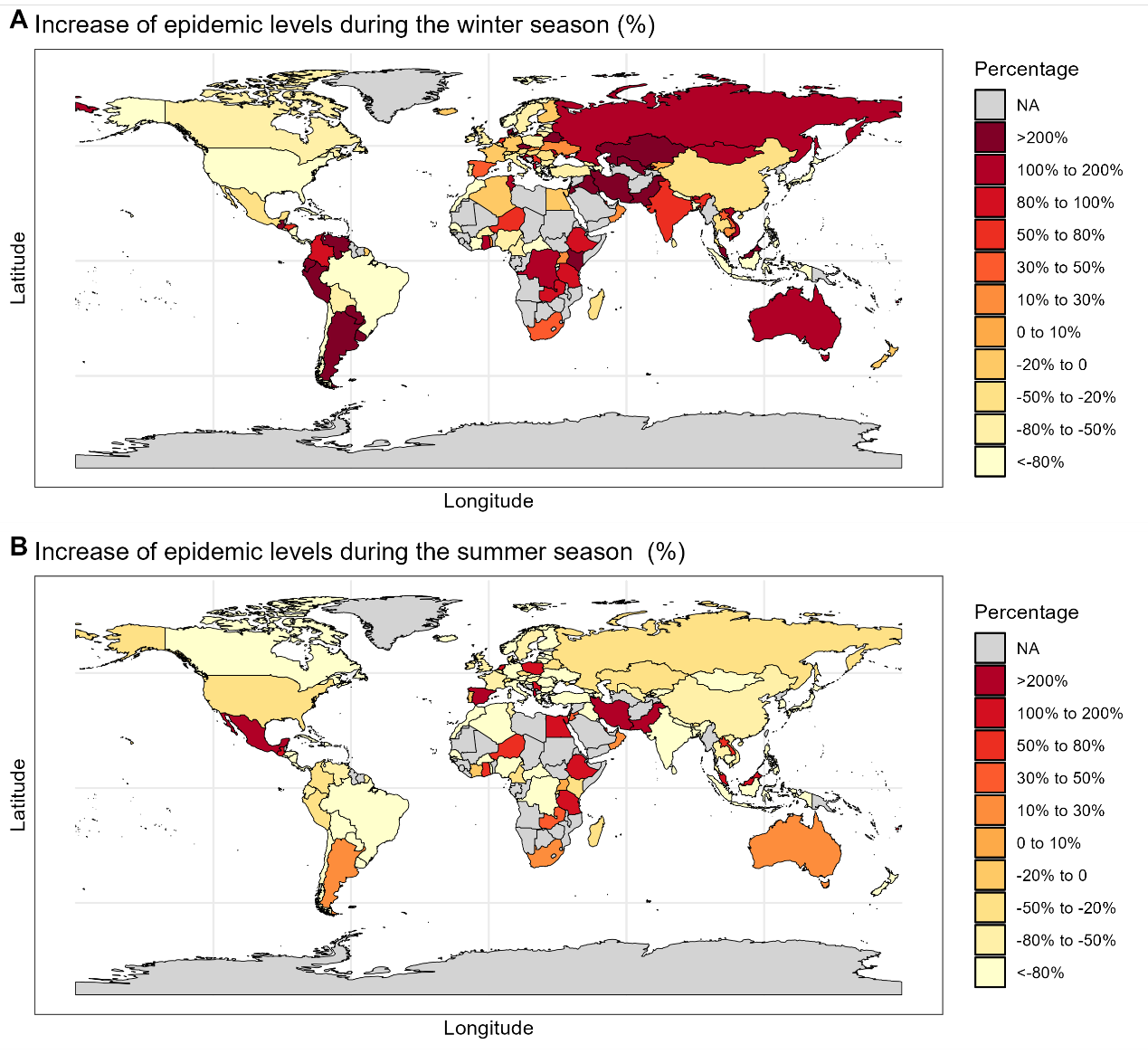


Figure S29. Increases of influenza B epidemic levels during the relaxation period.


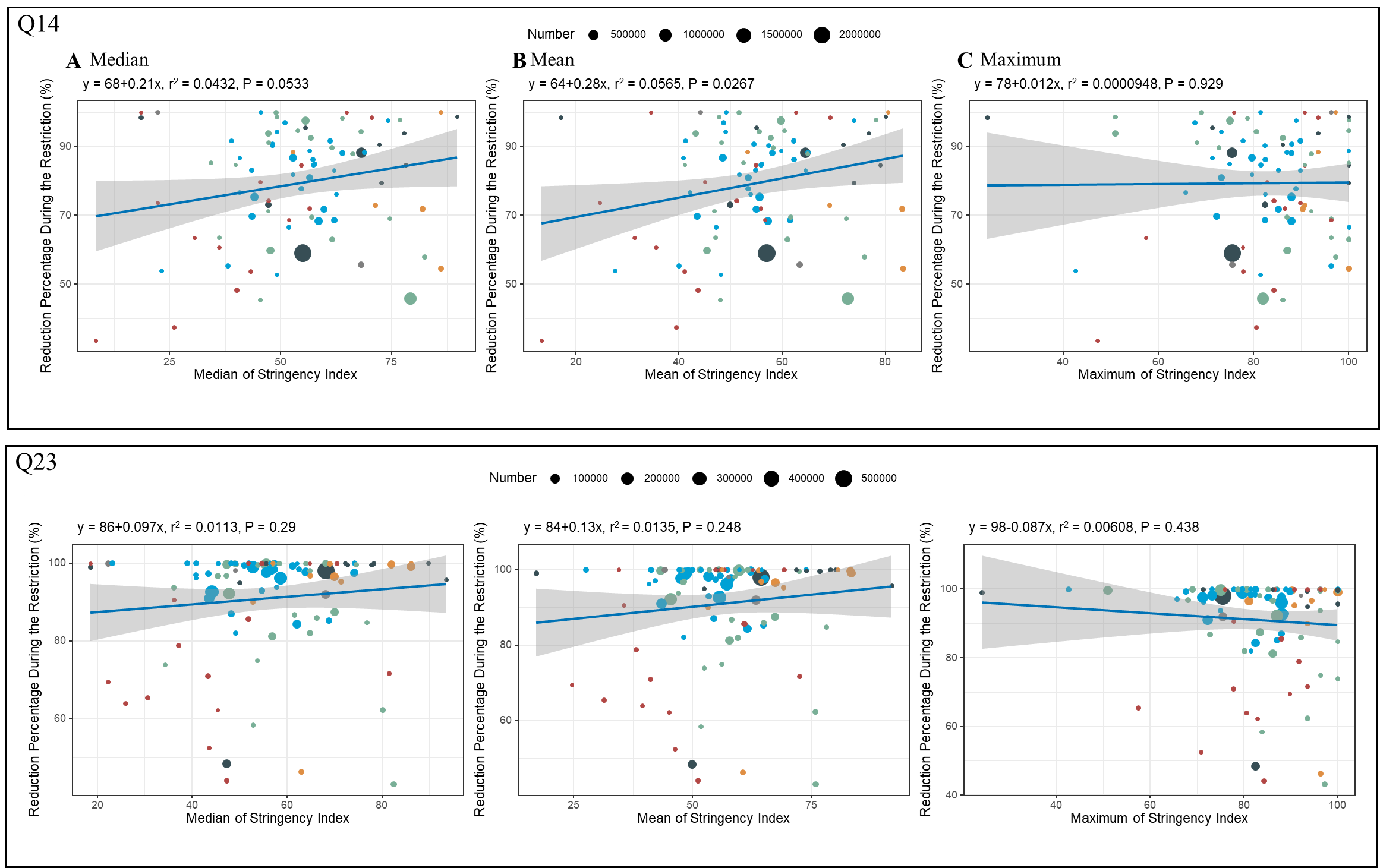


Figure S30. Relationship between country-specific reduction in number of total influenza cases and median, mean, and maximum stringency index during COVID-19 restriction period.


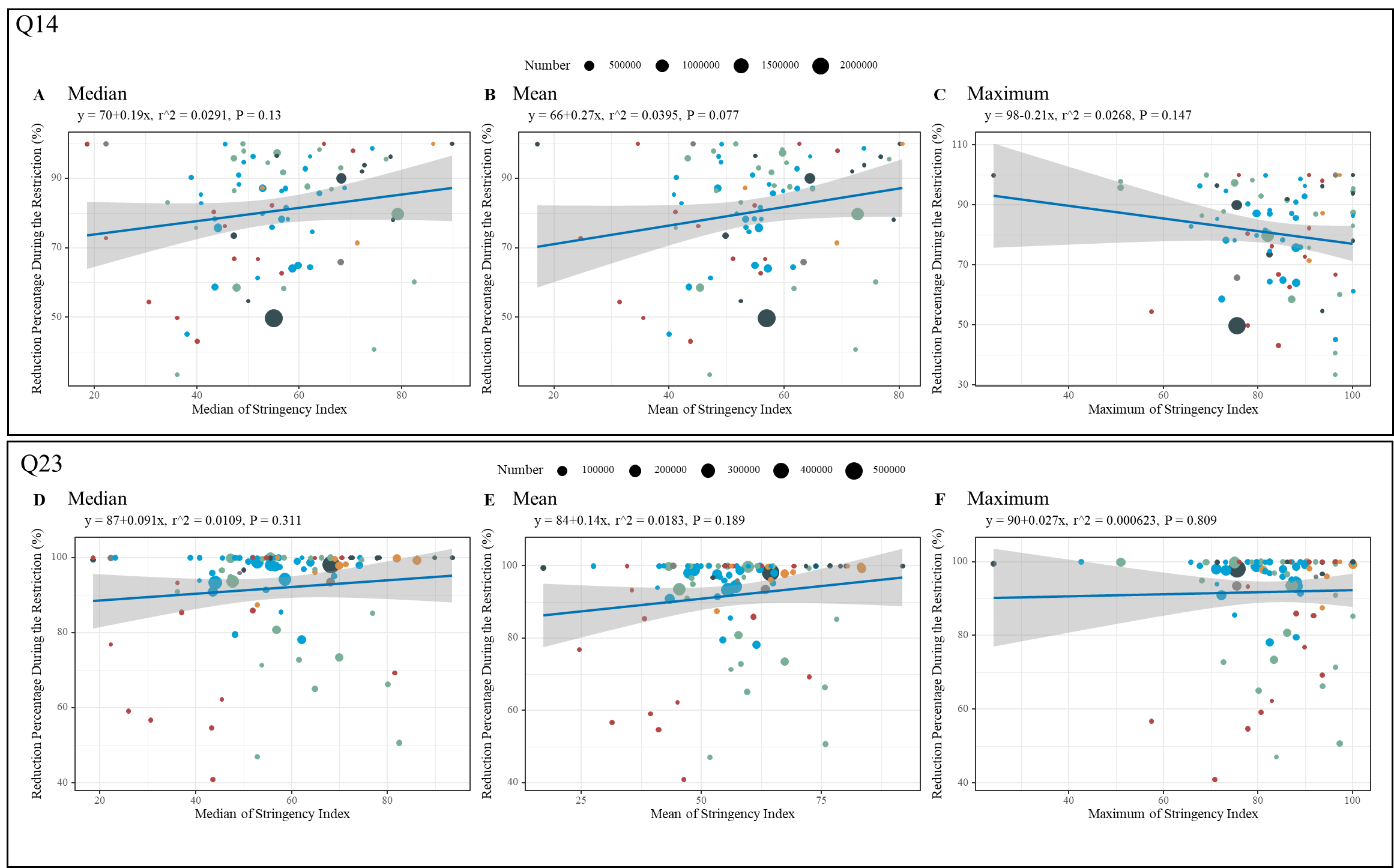


Figure S31. Relationship between country-specific reduction in number of influenza A cases and median, mean, and maximum stringency index during COVID-19 restriction period.


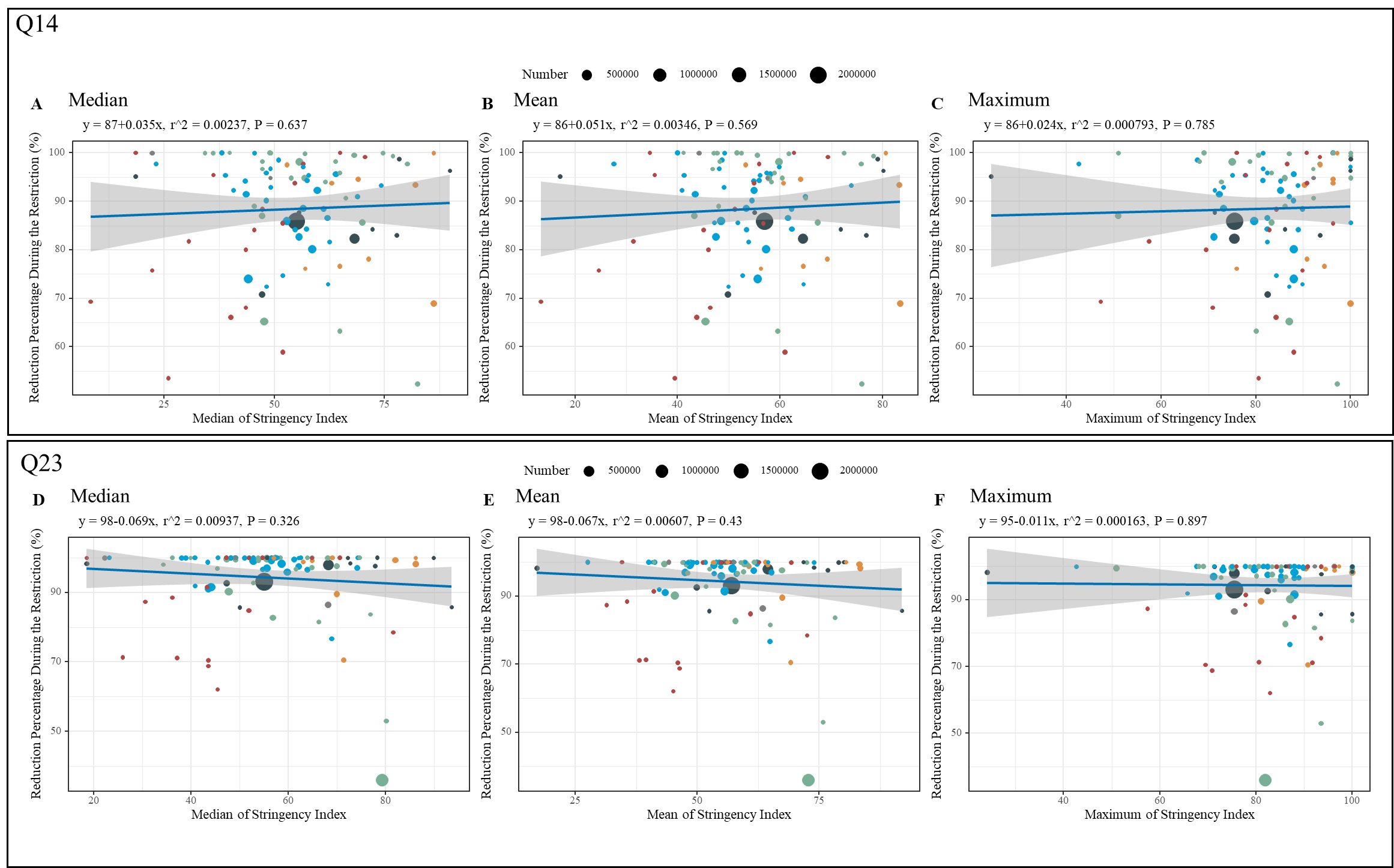


Figure S32. Relationship between country-specific reduction in number of influenza B cases and median, mean, and maximum stringency index during COVID-19 restriction period.


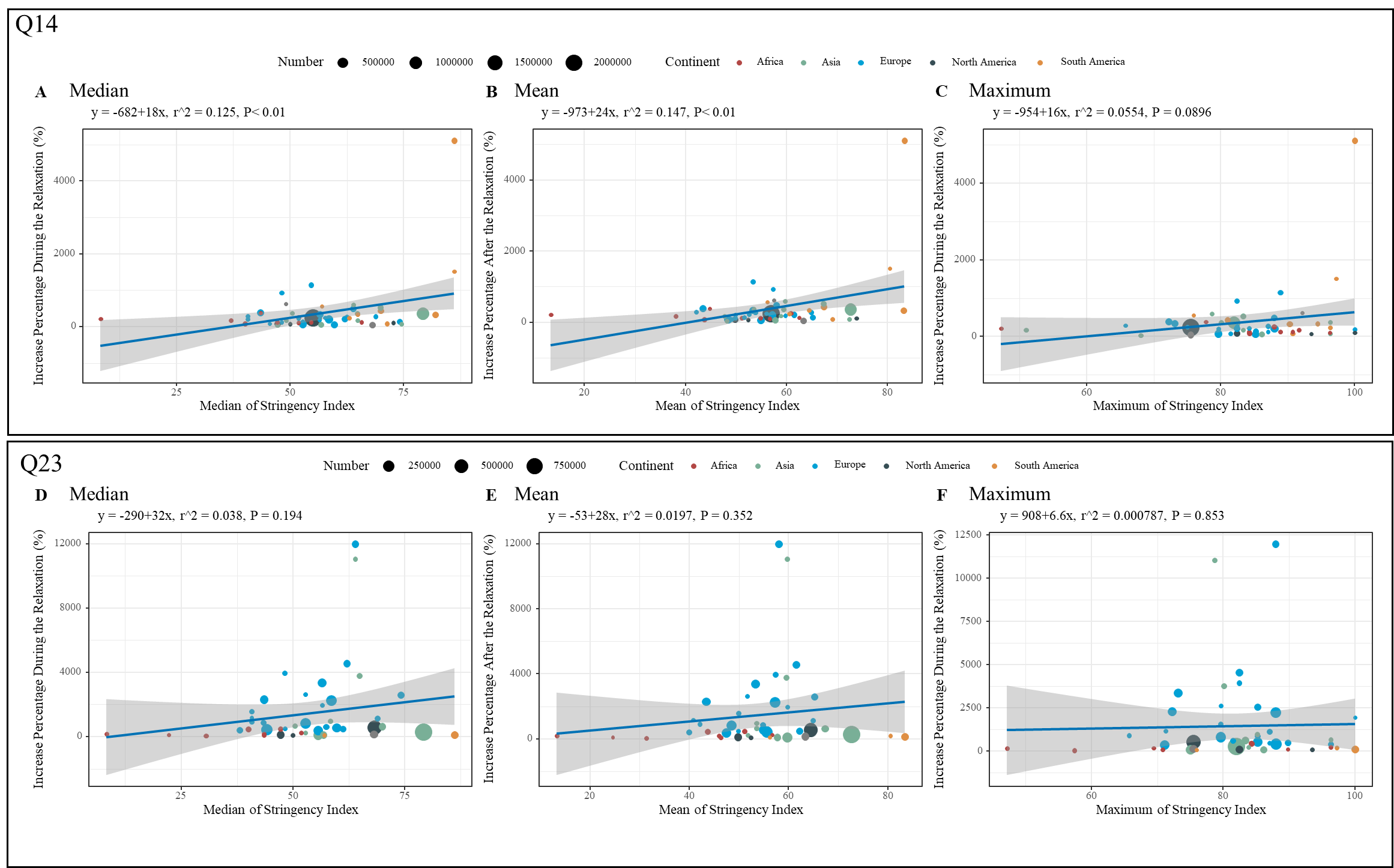


Figure S33. Relationship between country-specific increase in number of influenza A cases and median, mean, and maximum stringency index during relaxation period.


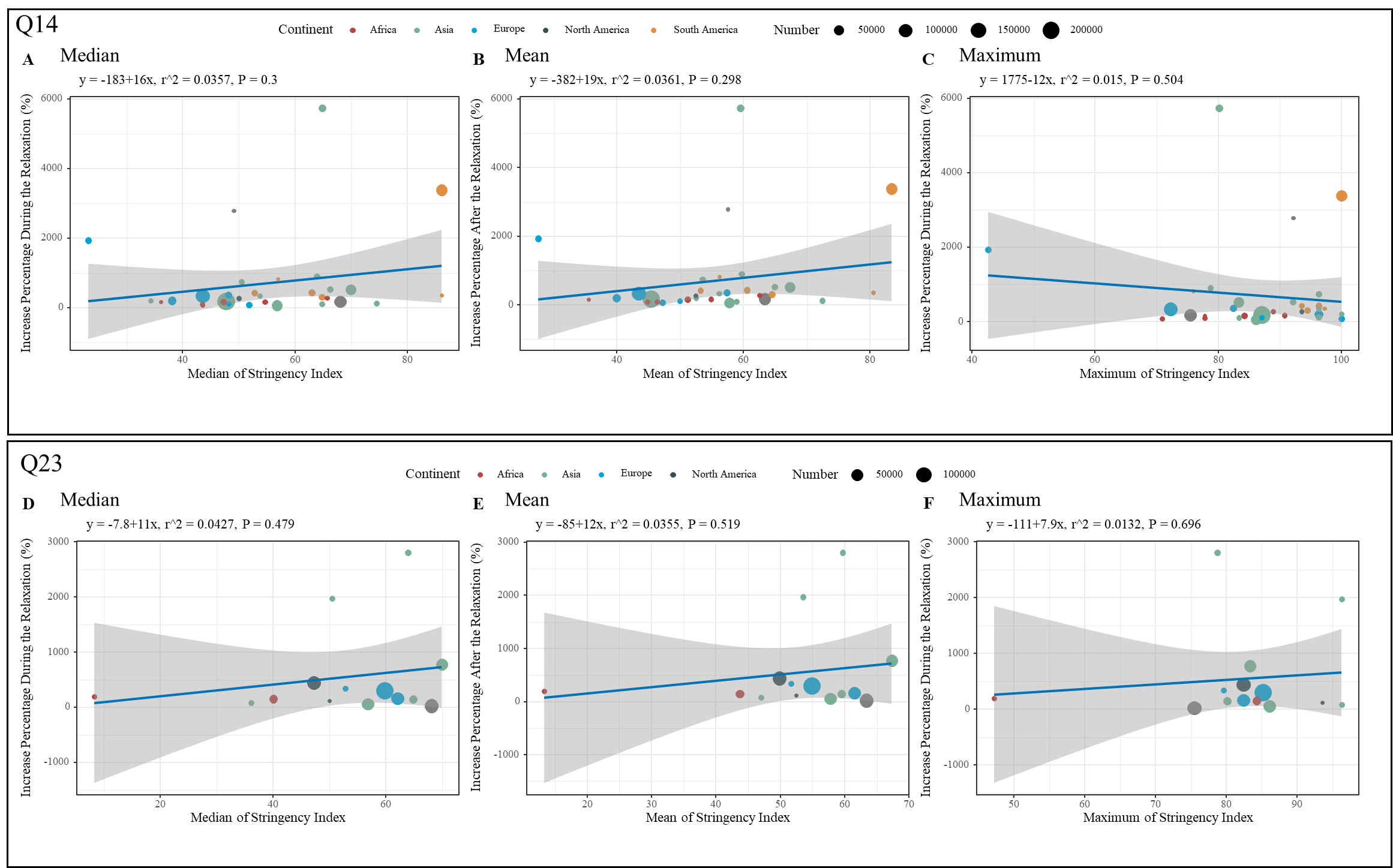


Figure S34. Relationship between country-specific increase in number of influenza B cases and median, mean, and maximum stringency index during relaxation period.

Table S8. The reduction during the restriction period in the Patient-level and CPRDGLOD data

| Country | Outcome | period | season | observed | prediction | reduction |
| --- | --- | --- | --- | --- | --- | --- |
| United Kingdom (CPRDGOLD) | influenza | Restriction | Winter season | 98.50 | 312.98 | -68.53 |
| United Kingdom (CPRDGOLD) | influenza | Restriction | Summer season | 20.54 | 42.54 | -51.72 |
| United Kingdom | influenza | Restriction | Winter season | 100.13 | 337.97 | -70.37 |
| United Kingdom | influenza | Restriction | Summer season | 36.88 | 58.42 | -36.87 |
| Belgium | influenza | Restriction | Winter season | 397.20 | 955.21 | -58.42 |
| Belgium | influenza | Restriction | Summer season | 74.27 | 96.27 | -22.85 |
| Spain | influenza | Restriction | Winter season | 441.84 | 2055.66 | -78.51 |
| Spain | influenza | Restriction | Summer season | 119.04 | 96.75 | 23.04 |
| France | influenza | Restriction | Winter season | 3109.37 | 7400.72 | -57.99 |
| France | influenza | Restriction | Summer season | 262.19 | 405.02 | -35.27 |
| Italy | influenza | Restriction | Winter season | 150.43 | 265.00 | -43.23 |
| Italy | influenza | Restriction | Summer season | 6.02 | 16.00 | -62.37 |
| Romania | influenza | Restriction | Winter season | 28.89 | 110.90 | -73.95 |
| Romania | influenza | Restriction | Summer season | 0.40 | 1.96 | -79.79 |
| United Kingdom (CPRDGOLD) | copd | Restriction | Winter season | 473.77 | 820.80 | -42.28 |
| United Kingdom (CPRDGOLD) | copd | Restriction | Summer season | 143.85 | 348.96 | -58.78 |
| United Kingdom | copd | Restriction | Winter season | 433.41 | 871.36 | -50.26 |
| United Kingdom | copd | Restriction | Summer season | 157.48 | 359.33 | -56.17 |
| Belgium | copd | Restriction | Winter season | 286.39 | 436.62 | -34.41 |
| Belgium | copd | Restriction | Summer season | 101.00 | 125.08 | -19.25 |
| Spain | copd | Restriction | Winter season | 230.70 | 295.71 | -21.99 |
| Spain | copd | Restriction | Summer season | 74.53 | 112.22 | -33.59 |
| France | copd | Restriction | Winter season | 473.23 | 589.01 | -19.66 |
| France | copd | Restriction | Summer season | 158.67 | 200.08 | -20.70 |
| Italy | copd | Restriction | Winter season | 310.32 | 78.67 | 294.45 |
| Italy | copd | Restriction | Summer season | 57.25 | 28.88 | 98.27 |
| Romania | copd | Restriction | Winter season | 286.32 | 363.79 | -21.29 |
| Romania | copd | Restriction | Summer season | 88.94 | 139.38 | -36.19 |
| United Kingdom (CPRDGOLD) | hypertension | Restriction | Winter season | 1956.14 | 2272.85 | -13.93 |
| United Kingdom (CPRDGOLD) | hypertension | Restriction | Summer season | 572.58 | 900.67 | -36.43 |
| United Kingdom | hypertension | Restriction | Winter season | 2013.04 | 2845.04 | -29.24 |
| United Kingdom | hypertension | Restriction | Summer season | 710.65 | 1160.15 | -38.74 |
| Belgium | hypertension | Restriction | Winter season | 588.43 | 643.77 | -8.60 |
| Belgium | hypertension | Restriction | Summer season | 252.46 | 221.58 | 13.93 |
| Spain | hypertension | Restriction | Winter season | 1310.89 | 1259.19 | 4.11 |
| Spain | hypertension | Restriction | Summer season | 405.56 | 477.93 | -15.14 |
| France | hypertension | Restriction | Winter season | 3933.96 | 4205.22 | -6.45 |
| France | hypertension | Restriction | Summer season | 1352.25 | 1433.42 | -5.66 |
| Italy | hypertension | Restriction | Winter season | 4141.50 | 1029.63 | 302.23 |
| Italy | hypertension | Restriction | Summer season | 1228.75 | 338.71 | 262.78 |
| Romania | hypertension | Restriction | Winter season | 1091.57 | 1157.01 | -5.66 |
| Romania | hypertension | Restriction | Summer season | 355.67 | 410.08 | -13.27 |
| United Kingdom (CPRDGOLD) | pneumonia | Restriction | Winter season | 653.25 | 858.65 | -23.92 |
| United Kingdom (CPRDGOLD) | pneumonia | Restriction | Summer season | 217.88 | 291.44 | -25.24 |
| United Kingdom | pneumonia | Restriction | Winter season | 525.96 | 898.05 | -41.43 |
| United Kingdom | pneumonia | Restriction | Summer season | 213.38 | 321.96 | -33.72 |
| Belgium | pneumonia | Restriction | Winter season | 130.57 | 229.48 | -43.10 |
| Belgium | pneumonia | Restriction | Summer season | 36.00 | 53.13 | -32.24 |
| Spain | pneumonia | Restriction | Winter season | 389.07 | 535.21 | -27.31 |
| Spain | pneumonia | Restriction | Summer season | 120.50 | 179.06 | -32.70 |
| France | pneumonia | Restriction | Winter season | 653.96 | 1167.31 | -43.98 |
| France | pneumonia | Restriction | Summer season | 141.38 | 315.71 | -55.22 |
| Italy | pneumonia | Restriction | Winter season | 98.82 | 80.41 | 22.90 |
| Italy | pneumonia | Restriction | Summer season | 25.42 | 23.65 | 7.49 |
| Romania | pneumonia | Restriction | Winter season | 841.82 | 1141.70 | -26.27 |
| Romania | pneumonia | Restriction | Summer season | 167.21 | 306.21 | -45.39 |

Table S9. The increases during the relaxation period in the Patient-level and CPRDGOLD data

| Country | Outcome | period | season | observed | prediction | increases |
| --- | --- | --- | --- | --- | --- | --- |
| United Kingdom (CPRDGOLD) | influenza | Relaxation | Winter season | 170.86 | 312.98 | -45.41 |
| United Kingdom (CPRDGOLD) | influenza | Relaxation | Summer season | 29.08 | 39.79 | -26.91 |
| United Kingdom | influenza | Relaxation | Winter season | 114.54 | 337.97 | -66.11 |
| United Kingdom | influenza | Relaxation | Summer season | 26.13 | 54.67 | -52.21 |
| Belgium | influenza | Relaxation | Winter season | 190.82 | 955.21 | -80.02 |
| Belgium | influenza | Relaxation | Summer season | 108.29 | 96.27 | 12.49 |
| Spain | influenza | Relaxation | Winter season | 242.61 | 2055.66 | -88.20 |
| Spain | influenza | Relaxation | Summer season | 63.83 | 131.00 | -51.27 |
| France | influenza | Relaxation | Winter season | 3520.46 | 7095.28 | -50.38 |
| France | influenza | Relaxation | Summer season | 1024.63 | 405.02 | 152.98 |
| Italy | influenza | Relaxation | Winter season | 205.11 | 265.00 | -22.60 |
| Italy | influenza | Relaxation | Summer season | 33.71 | 16.00 | 110.68 |
| Romania | influenza | Relaxation | Winter season | 58.50 | 110.90 | -47.25 |
| Romania | influenza | Relaxation | Summer season | 16.17 | 1.96 | 725.53 |
| United Kingdom (CPRDGOLD) | copd | Relaxation | Winter season | 400.86 | 820.80 | -51.16 |
| United Kingdom (CPRDGOLD) | copd | Relaxation | Summer season | 206.08 | 346.58 | -40.54 |
| United Kingdom | copd | Relaxation | Winter season | 129.36 | 871.36 | -85.15 |
| United Kingdom | copd | Relaxation | Summer season | 150.08 | 357.96 | -58.07 |
| Belgium | copd | Relaxation | Winter season | 81.71 | 436.62 | -81.28 |
| Belgium | copd | Relaxation | Summer season | 90.08 | 125.08 | -27.98 |
| Spain | copd | Relaxation | Winter season | 50.21 | 295.71 | -83.02 |
| Spain | copd | Relaxation | Summer season | 31.67 | 123.75 | -74.41 |
| France | copd | Relaxation | Winter season | 174.86 | 563.73 | -68.98 |
| France | copd | Relaxation | Summer season | 160.42 | 200.08 | -19.83 |
| Italy | copd | Relaxation | Winter season | 89.79 | 78.67 | 14.13 |
| Italy | copd | Relaxation | Summer season | 116.75 | 28.88 | 304.33 |
| Romania | copd | Relaxation | Winter season | 76.50 | 363.79 | -78.97 |
| Romania | copd | Relaxation | Summer season | 109.33 | 139.38 | -21.55 |
| United Kingdom (CPRDGOLD) | hypertension | Relaxation | Winter season | 1516.00 | 2272.85 | -33.30 |
| United Kingdom (CPRDGOLD) | hypertension | Relaxation | Summer season | 791.92 | 889.75 | -11.00 |
| United Kingdom | hypertension | Relaxation | Winter season | 520.64 | 2845.04 | -81.70 |
| United Kingdom | hypertension | Relaxation | Summer season | 678.92 | 1149.79 | -40.95 |
| Belgium | hypertension | Relaxation | Winter season | 143.21 | 643.77 | -77.75 |
| Belgium | hypertension | Relaxation | Summer season | 209.25 | 221.58 | -5.57 |
| Spain | hypertension | Relaxation | Winter season | 222.43 | 1259.19 | -82.34 |
| Spain | hypertension | Relaxation | Summer season | 146.33 | 513.42 | -71.50 |
| France | hypertension | Relaxation | Winter season | 1205.43 | 4022.49 | -70.03 |
| France | hypertension | Relaxation | Summer season | 1186.50 | 1433.42 | -17.23 |
| Italy | hypertension | Relaxation | Winter season | 789.79 | 1029.63 | -23.29 |
| Italy | hypertension | Relaxation | Summer season | 1334.58 | 338.71 | 294.02 |
| Romania | hypertension | Relaxation | Winter season | 270.21 | 1157.01 | -76.65 |
| Romania | hypertension | Relaxation | Summer season | 391.33 | 410.08 | -4.57 |
| United Kingdom (CPRDGOLD) | pneumonia | Relaxation | Winter season | 416.93 | 858.65 | -51.44 |
| United Kingdom (CPRDGOLD) | pneumonia | Relaxation | Summer season | 218.58 | 286.83 | -23.79 |
| United Kingdom | pneumonia | Relaxation | Winter season | 163.29 | 898.05 | -81.82 |
| United Kingdom | pneumonia | Relaxation | Summer season | 162.67 | 315.75 | -48.48 |
| Belgium | pneumonia | Relaxation | Winter season | 54.00 | 229.48 | -76.47 |
| Belgium | pneumonia | Relaxation | Summer season | 39.92 | 53.13 | -24.86 |
| Spain | pneumonia | Relaxation | Winter season | 105.86 | 535.21 | -80.22 |
| Spain | pneumonia | Relaxation | Summer season | 48.83 | 203.17 | -75.96 |
| France | pneumonia | Relaxation | Winter season | 377.07 | 1121.49 | -66.38 |
| France | pneumonia | Relaxation | Summer season | 212.50 | 315.71 | -32.69 |
| Italy | pneumonia | Relaxation | Winter season | 47.64 | 80.41 | -40.75 |
| Italy | pneumonia | Relaxation | Summer season | 30.04 | 23.65 | 27.05 |
| Romania | pneumonia | Relaxation | Winter season | 267.86 | 1141.70 | -76.54 |
| Romania | pneumonia | Relaxation | Summer season | 234.33 | 306.21 | -23.47 |


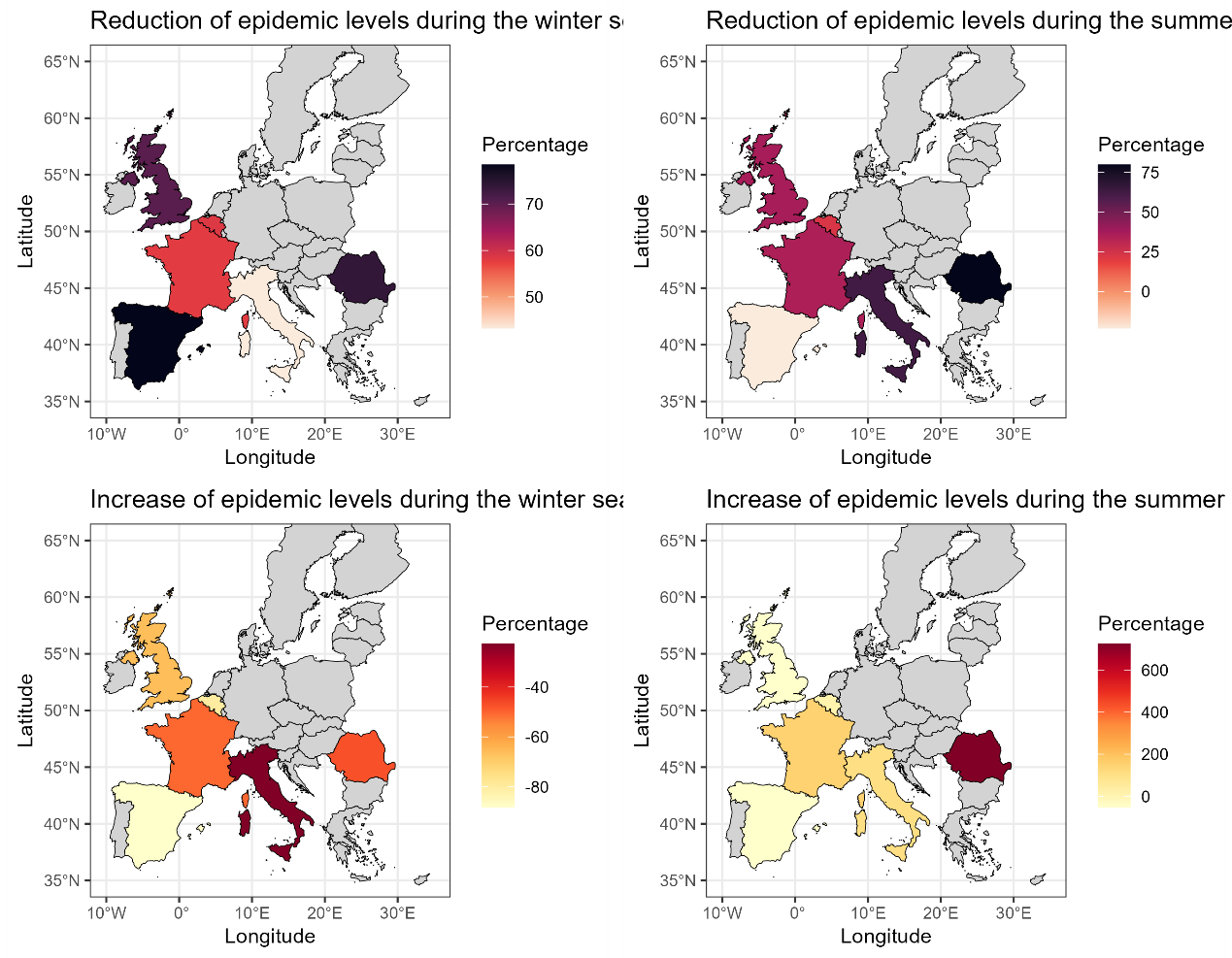


Figure S35: the reduction and increases in Influenza in the Patient-level


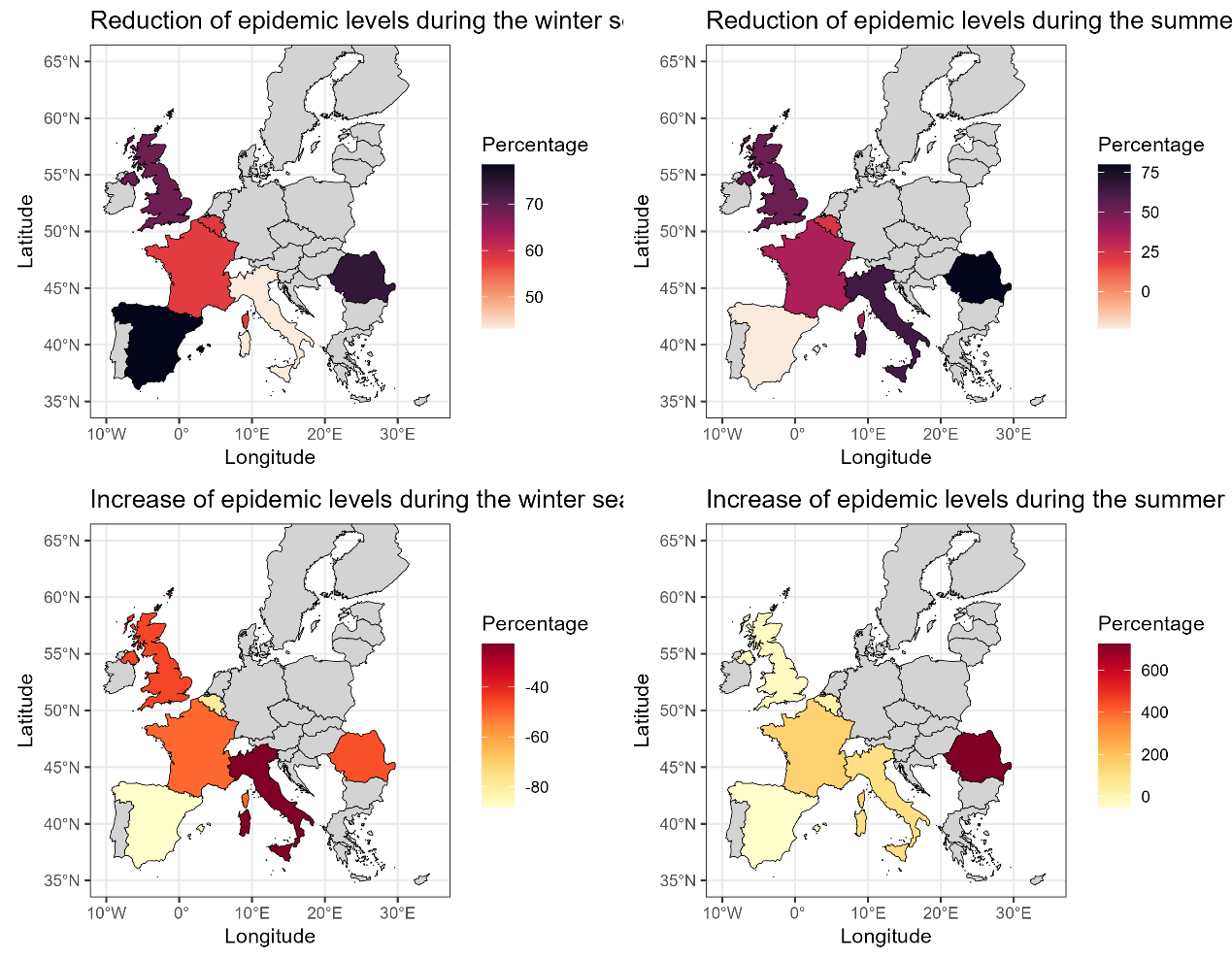


Figure S36: the reduction and increases in Influenza in the Patient-level.


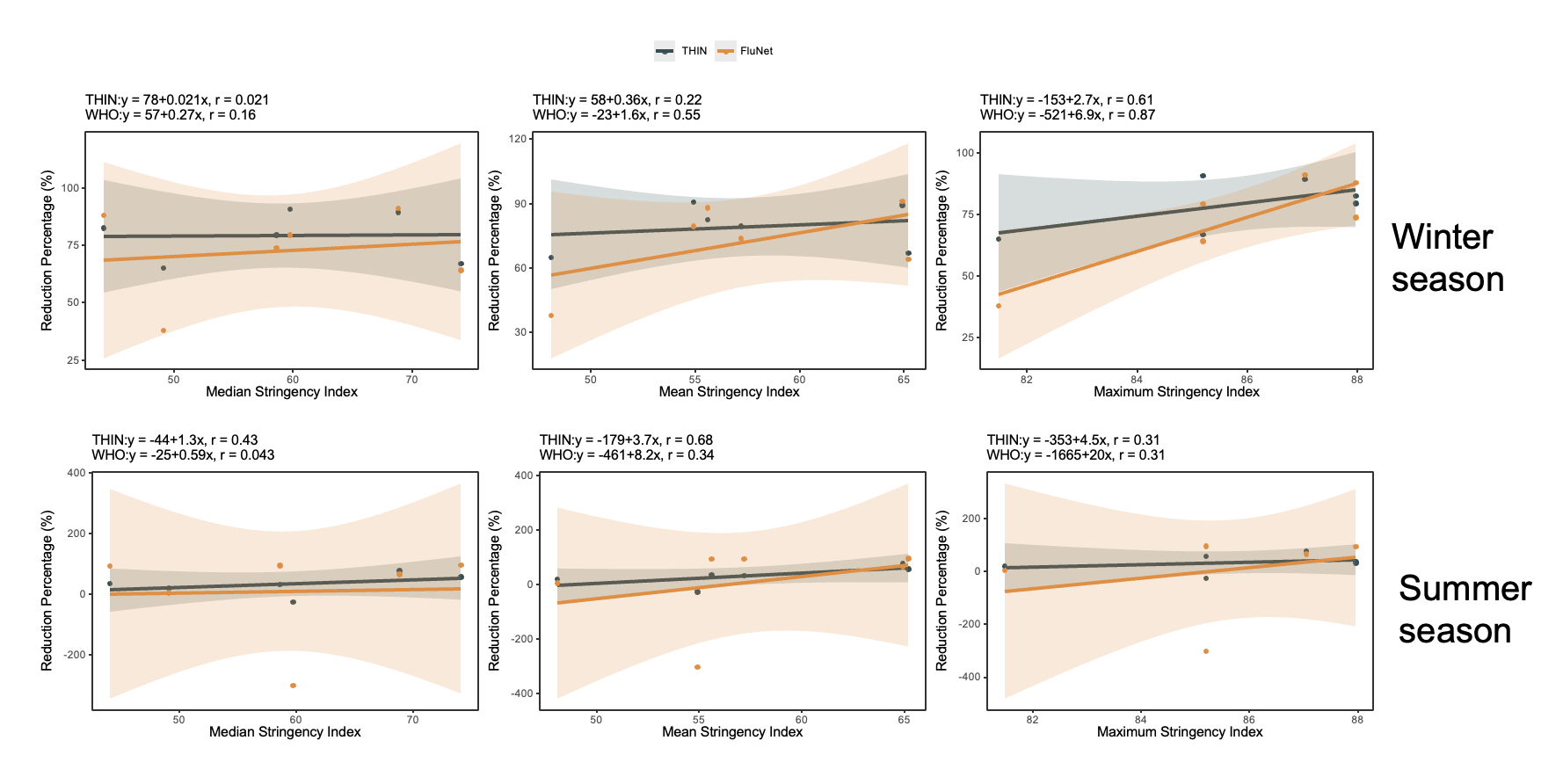


Figure S37: Relationship between Country-Specific reduction in Influenza Cases and Median, Mean, and Maximum Stringency Index During Relaxation Period in the patient-level and FluNet Data


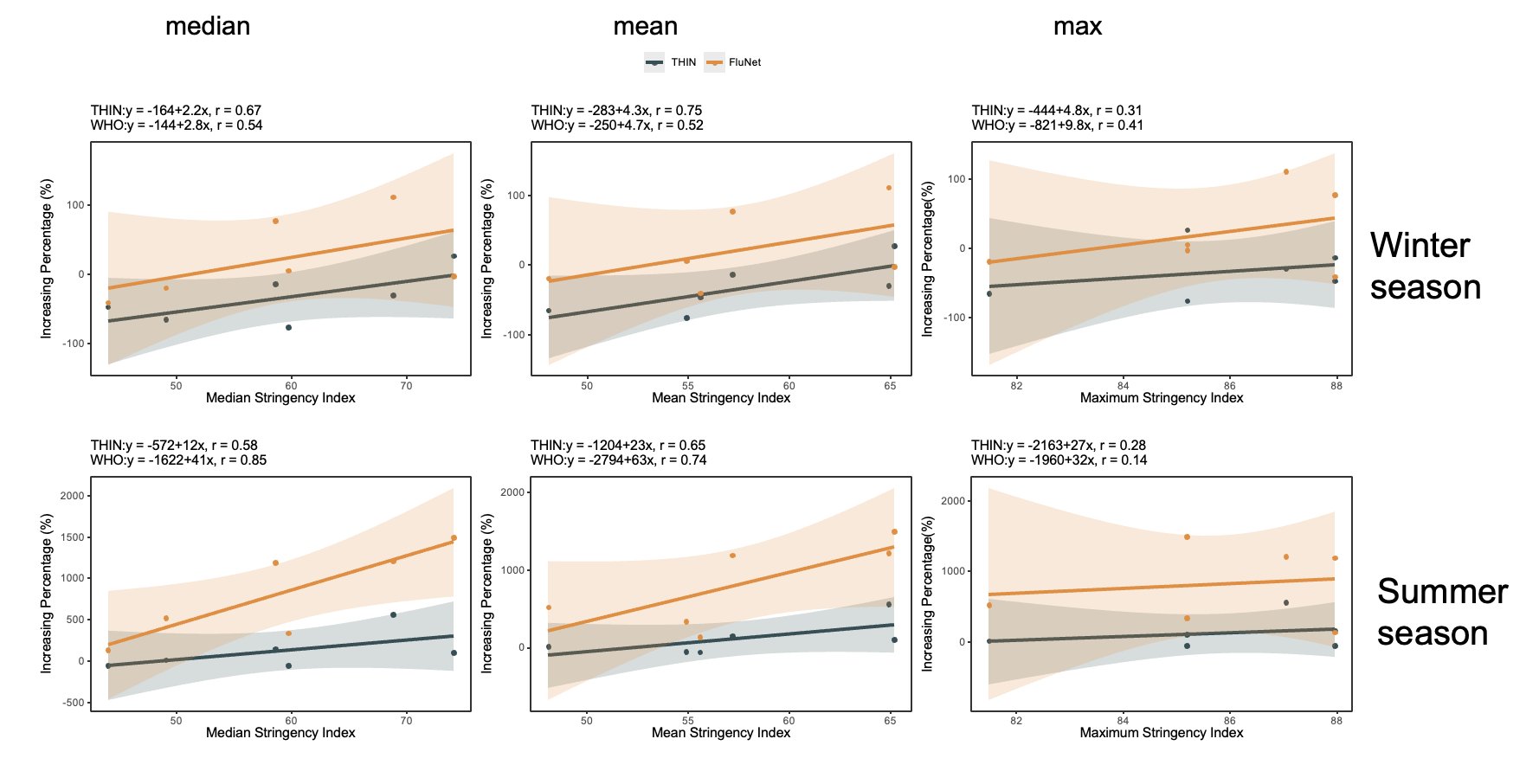


Figure S38: Relationship between Country-Specific Increase in Influenza Cases and Median, Mean, and Maximum Stringency Index During Relaxation Period in the patient-level and FluNet Data


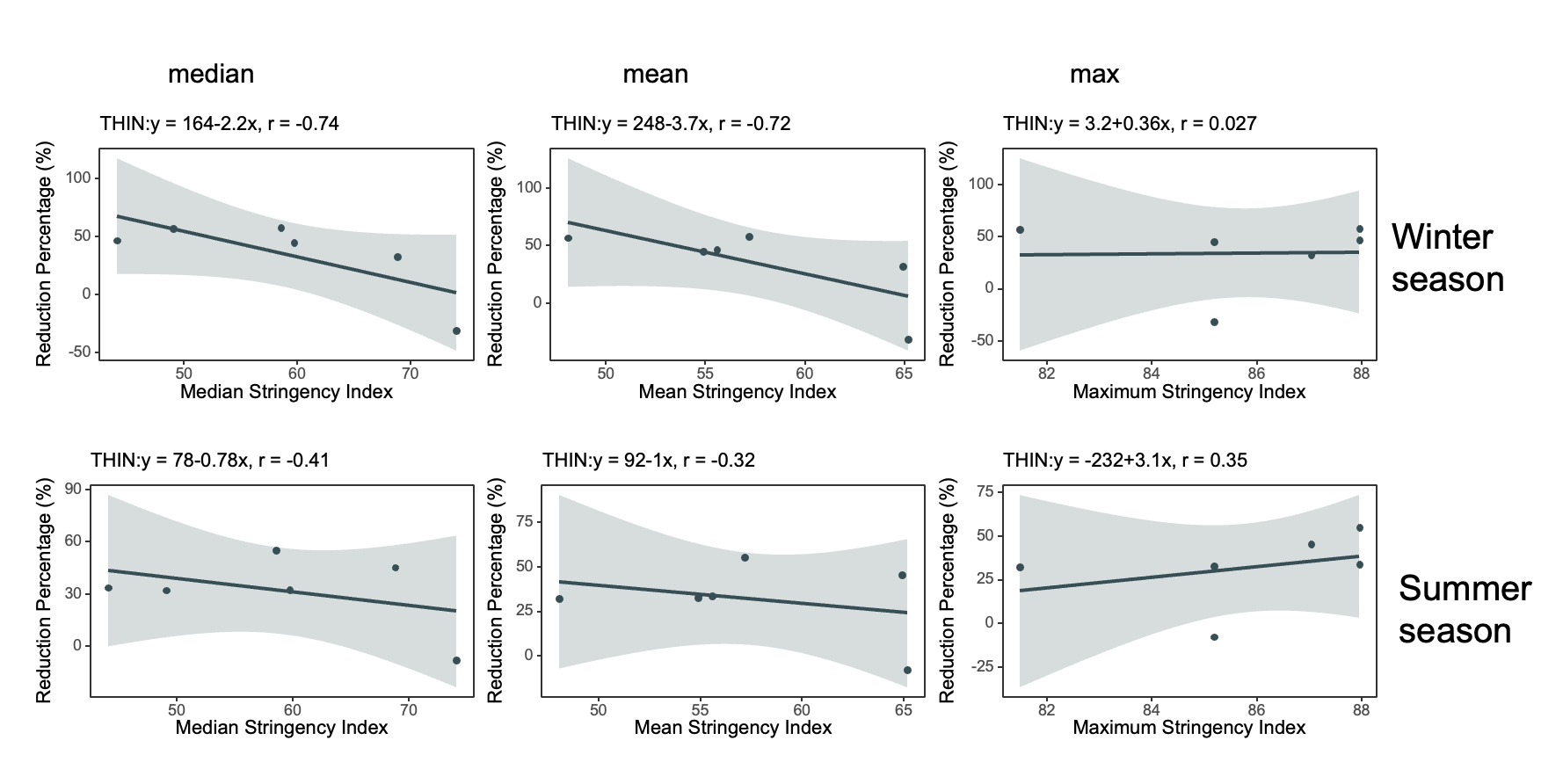


Figure S39: Relationship between Country-Specific reduction in pneumonia Cases and Median, Mean, and Maximum Stringency Index During Relaxation Period in the Patient-level


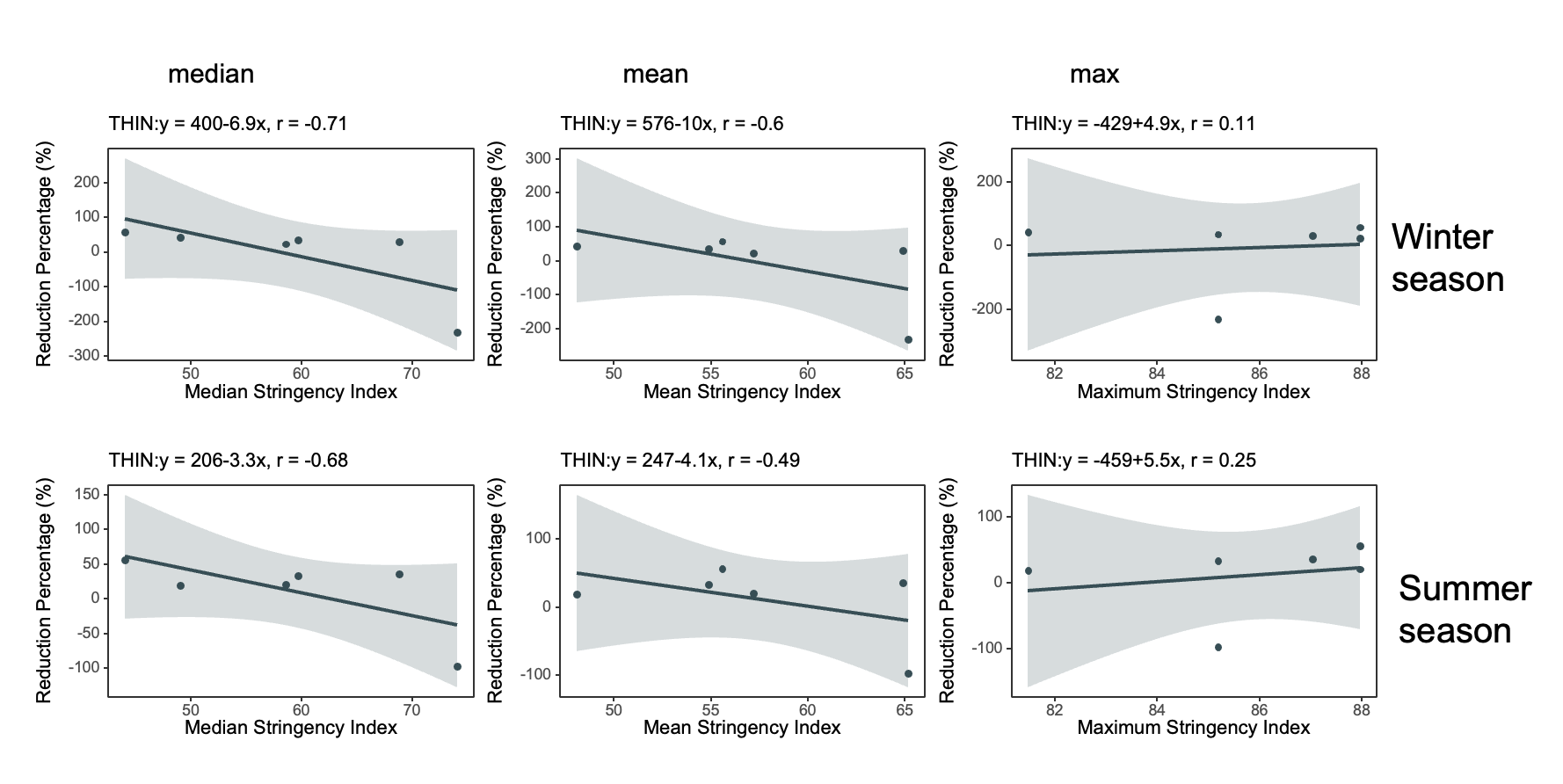


Figure S40: Relationship between Country-Specific reduction in chronic obstructive pulmonary disease (COPD) Cases and Median, Mean, and Maximum Stringency Index During Relaxation Period in the Patient-level.


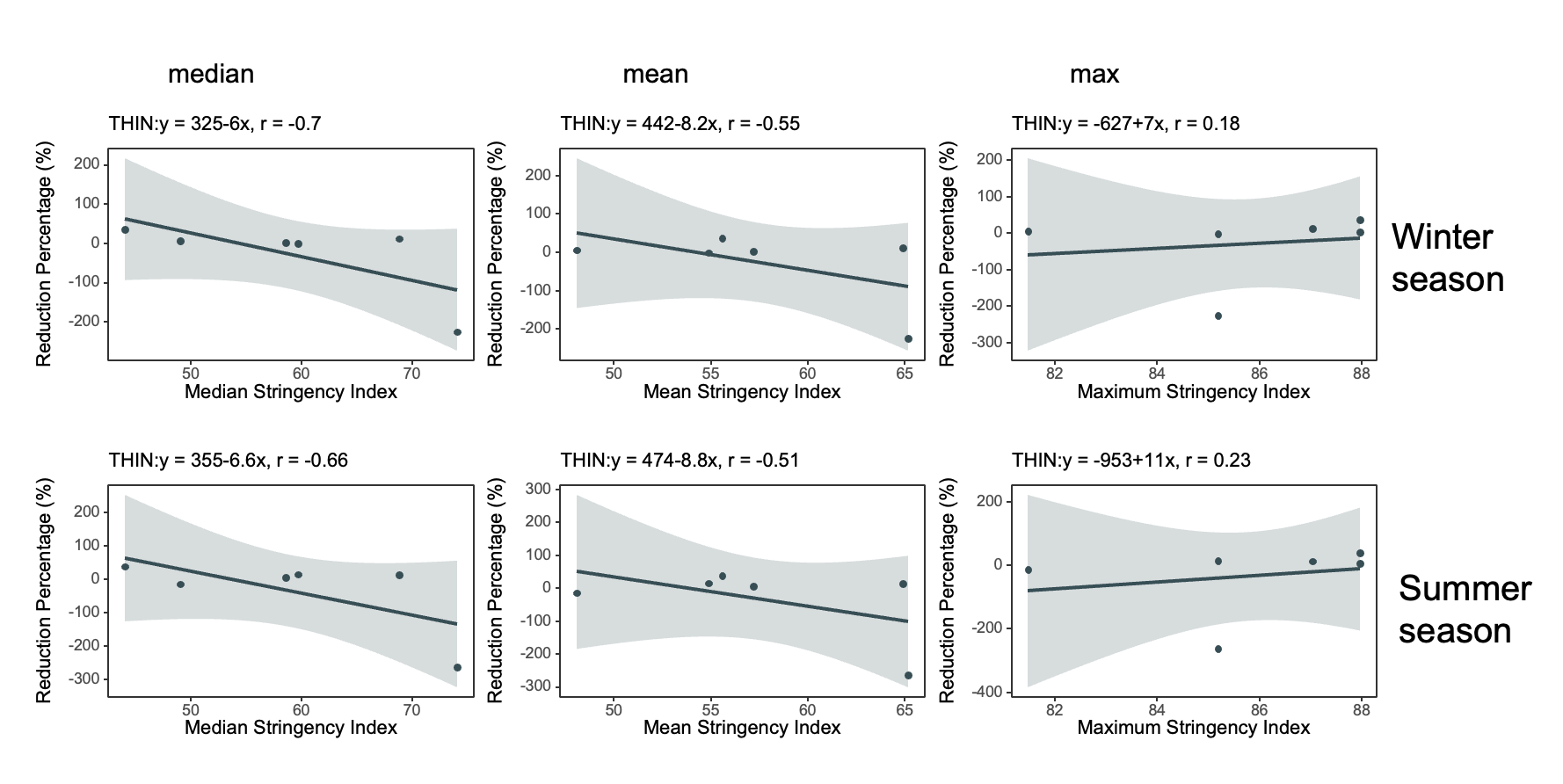


Figure S41: Relationship between Country-Specific reduction in hypertension Cases and Median, Mean, and Maximum Stringency Index During Relaxation Period in the Patient-level.
